# Evolutionary reconstruction, nomenclature and functional meta-analysis of the Kiwellin protein family

**DOI:** 10.3389/fpls.2022.1034708

**Published:** 2022-12-22

**Authors:** Paul Klemm, Marvin Christ, Florian Altegoer, Johannes Freitag, Gert Bange, Marcus Lechner

**Affiliations:** ^1^ Center for Synthetic Microbiology (SYNMIKRO), Philipps-University Marburg, Marburg, Germany; ^2^ Institute of Microbiology, Heinrich Heine University Dusseldorf, Düsseldorf, Germany; ^3^ Department of Biology, Philipps-University Marburg, Marburg, Germany; ^4^ Molecular Physiology of Microbes, Max-Planck Institute for Terrestrial Microbiology, Marburg, Germany

**Keywords:** Kiwellins, plants, interaction, pathogen, symbionts, evolution, classification, nomenclature

## Abstract

Crop diseases caused by pathogens critically affect global food security and plant ecology. Pathogens are well adapted to their host plants and have developed sophisticated mechanisms allowing successful colonization. Plants in turn have taken measures to counteract pathogen attacks resulting in an evolutionary arms race. Recent studies provided mechanistic insights into how two plant Kiwellin proteins from *Zea mays* mitigate the activity of the chorismate mutase Cmu1, a virulence factor secreted by the fungal pathogen *Ustilago maydis* during maize infection. Formerly identified as human allergens in kiwifruit, the biological function of Kiwellins is apparently linked to plant defense. We combined the analysis of proteome data with structural predictions to obtain a holistic overview of the Kiwellin protein family, that is subdivided into proteins with and without a N-terminal kissper domain. We found that Kiwellins are evolutionarily conserved in various plant species. At median five Kiwellin paralogs are encoded in each plant genome. Structural predictions revealed that Barwin-like proteins and Kiwellins cannot be discriminated purely at the sequence level. Our data shows that Kiwellins emerged in land plants (embryophyta) and are not present in fungi as suggested earlier. They evolved *via* three major duplication events that lead to clearly distinguishable subfamilies. We introduce a systematic Kiwellin nomenclature based on a detailed evolutionary reconstruction of this protein family. A meta-analysis of publicly available transcriptome data demonstrated that Kiwellins can be differentially regulated upon the interaction of plants with pathogens but also with symbionts. Furthermore, significant differences in Kiwellin expression levels dependent on tissues and cultivars were observed. In summary, our study sheds light on the evolution and regulation of a large protein family and provides a framework for a more detailed understanding of the molecular functions of Kiwellins.

## Introduction

A better understanding of plant diseases caused by viruses, bacteria, and fungi as well as by oomycetes is critical to improve global food security. In many cases, pathogens employ secreted effector proteins to manipulate the host plant and promote infection (Stergiopoulos and de Wit, 2009; Rocafort et al., 2020). Effectors exhibit a wide range of functions, e.g. they can mask the pathogen, (down)-regulate host defense mechanisms, or target defense enzymes or toxins to render them harmless (Lanver et al., 2018). In turn, plants have evolved various defense mechanisms. Upon pathogen contact, pattern recognition receptors (PRR) in the plant plasma membrane recognize conserved molecules on the surface of the microorganisms, such as flagellin, chitin, or glucans from bacteria, fungi, and oomycetes respectively (Jones and Dangl, 2006; Cook et al., 2015). These microorganism-associated molecular patterns (MAMPs) lead to MAMP-triggered immunity (MTI) (Cook et al., 2015) triggering response mechanisms to restrict damage caused by the pathogen. Plants can furthermore recognize effectors that are secreted or translocated into the plant cytoplasm, resulting in the activation of a second layer of defense, the so-called effector-triggered immunity (ETI) (Du et al., 2016). Both types of responses are tightly interconnected and thus referred to as the plant immune system (Jones and Dangl, 2006; Nguyen et al., 2021). Recently, two studies suggested a crucial role for maize Kiwellins as proteins counteracting pathogen attack (Han et al., 2019; Altegoer et al., 2020).

Two *Z. mays* Kiwellins specifically bind to the secreted chorismate mutase Cmu1 of the smut fungus *U. maydis* and inhibit its enzymatic activity (Han et al., 2019; Altegoer et al., 2020). Cmu1 was shown previously to down-regulate salicylic acid synthesis in the host by diverting its substrate chorismate to the phenylpropanoid pathway, thereby decreasing maize resistance to *U. maydis* (Djamei et al., 2011). Kiwellins were originally identified in kiwifruit (*Actinidia spp.*) in which they account for about 30% of the total protein content (Tamburrini et al., 2005). Kiwifruit can cause allergies in humans (Fine, 1981; Wang et al., 2019). It was shown that Kiwellin proteins contribute to the allergic response and are recognized by immunoglobulin E (Tamburrini et al., 2005; Ciardiello et al., 2009). The crystal structure of a Kiwellin from *Actinidia chinensis* revealed that it is a modular protein formed by an N-terminal 4 kDa kissper domain and a C-terminal core domain (Hamiaux et al., 2014). Pore-forming activity was reported for the kissper domain in synthetic lipid-bilayers while the Kiwellin-core-domain contains a double-psi *β*-barrel fold and a *β* -hairpin (Tuppo et al., 2008). Kiwellins have a high structural similarity to another class of plant defense proteins termed Barwin. Barwin and Barwin-like proteins are pathogenesis-related (PR) proteins belonging to the PR4 family. This family is divided into two classes, Barwin-like proteins with a chitin-binding domain (class I) and without this domain (class II) (Sinha et al., 2014). These proteins are mainly found in plants but also occur in bacteria, algae, and fungi. The functions of the Barwin domain are manifold. They can bind sugars, cleave RNA and DNA depending on divalent cations, and show antifungal activity (Dabravolski and Frenkel, 2021).

Due to the identification of a biological role of Kiwellins as plant defense molecules and their widespread appearance in the kingdom of plants (Bange and Altegoer, 2019; Han et al., 2019) it was tempting to speculate about an evolutionarily conserved role of Kiwellins as regulators of biotic interactions. Therefore, we set out for a systematic phylogenetic and structural investigation of the Kiwellin protein family to provide a framework for further research on this large yet relatively uncharacterized group of proteins. We provide a detailed phylogenetic reconstruction of Kiwellin evolution based on published proteome data and introduce a nomenclature for these proteins. In addition, we show that many proteins annotated as Kiwellin-like are actually Barwin-like proteins and that Kiwellins are probably restricted to land plants. Finally, we reanalyzed 31 publicly available transcriptome data obtained from plants exposed to biotic and abiotic stresses. This uncovered remarkable transcriptional regulation patterns for Kiwellin encoding transcripts upon interaction of plants with microorganisms. These data hence suggest a crucial role of Kiwellin proteins as modulators of plant-microbe interactions.

## Material and methods

### Kiwellin annotation

**Figure 1:**
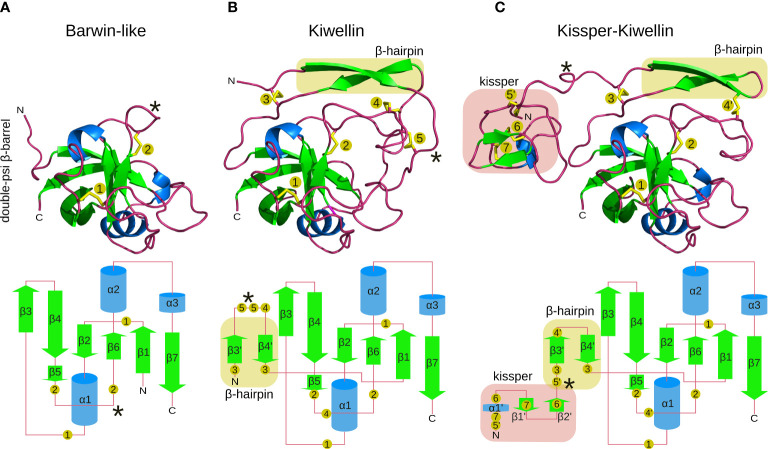
Structure-models of Barwin-like **(A)**, Kiwellin **(B)**, and Kissper-Kiwellin **(C)** proteins based on consensus sequences of all proteins identified for each group (signal peptide removed). Elements visible in the 3D structure on the top are indicated in a planar visualization on the bottom with identical coloring (green: *β*-sheets, blue: α-helices, red: loop regions). Highlighted in yellow is the *β*-hairpin and in red is the kissper domain. Numbered, yellow circles indicate the respective disulfide-boundforming cysteine residues. A loop region with variable length is indicated by *.

Kiwellins were annotated in all complete reference proteomes provided by UniProt, v2022_01 (Consortium, 2019). We conducted an iterative procedure that combines an initial sequence-based model with a structure-based filtering strategy. Initially, published sequences from Han et al. (2019) were aligned using muscle v3.8.1551 (Edgar, 2004). A profile Hidden Markov Model was built from this alignment using HMMer v3.2.1 (Eddy, 1998) and then used to mine the proteomes of all kingdoms of life (adaptive e-value cutoff based on false positives, see **Supplementary Data 1** chapter 4). The resulting proteins were trimmed at the signal peptide cleavage site which was predicted with SignalP v5.0b (Almagro Armenteros et al., 2019) if present within the first half. The region upstream of this site is cleaved *in vivo* and is thus not relevant for structural prediction. We employed AlphaFold2 v2.0.0 (Jumper et al., 2021) to accurately assess structural elements, which were then used to determine the fold type, see **Figure 1**. Predicted structures were superimposed with the Kiwellin crystal structure provided by Han et al. (2019) using PyMOL (DeLano and Bromberg, 2004) and compared based on the presence of structural elements, RMSD and overlap (see **Supplemental Data 1** chapter 4 for details). In this way, false-positive hits, e.g. Barwin-like proteins, could be distinguished from Kiwellins and Kissper-Kiwellins. The process was iterated multiple times, further improving the profile Hidden Markov Model. We trained separate models for Kiwellins and Kissper-Kiwellins. In parallel, we trained a model for false positives e.g. Barwin-like proteins that allowed us to filter out members of this group already at the sequence-based stages.

In total, we identified 915 Kiwellins in 142 land plants (embryophyta) and one fungal species. No Kiwellins were detected in bacteria, archaea, or viruses/phages. A detailed description of the pipeline can be found in **Supplementary Data 1** chapter 4. The implementation of the workflow along with the models generated at the last iteration is available *via* our GitLab repository, see *Data Availability Statement*.

### Evolutionary reconstruction

The evolution of Kiwellins was reconstructed based on a phylogenetic gene tree that was modeled onto the associated species tree. Phylogenetic conflicts that arise in this process are resolved using a maximum likelihood-based approach concerning speciation, duplication, or loss events within the gene lineages.

First, all 915 Kiwellins with trimmed signal peptides were aligned using muscle v3.8.1551 (Edgar, 2004) to reconstruct the gene tree. The best-fit model for amino acid replacement with respect to the Bayesian information criterion (BIC) was determined using IQ-TREE v2.0.3 (Nguyen et al., 2015). The phylogenetic tree was then reconstructed with 100k bootstrap iterations based on the reported general amino-acid exchange rate matrix (WAG) (Whelan and Goldman, 2001) with a FreeRate model (Yang, 1995) of 8 categories of rate heterogeneity across sites, WAG+R8.

Second, the species tree was compiled based on the Open Tree of Life Synthetic Tree v13.4 (OpenTree, 2019a). The relevant subtree was re-rooted and pruned to the species with annotated Kiwellins within the reference proteomes provided by UniProt using ete3 v3.0.0b34 (Huerta-Cepas et al., 2016) based on the open tree taxonomy v3.3 (OpenTree, 2019b). To reduce the complexity, cultivars/subspecies were merged into a single species node representing all strains. As the Synthetic Tree does not encode distances for any species, these were estimated based on their core proteome. Orthologous proteins were determined using Proteinortho v6.1.1 (Lechner et al., 2011) with a fairly high e-value of 10^-20^. The 204 orthologous groups that covered all species were aligned using muscle v3.8.1551 (Edgar, 2004). Alignment columns with more than 90% gap content were dismissed, to reduce complexity. The alignments of all orthologous groups were then concatenated to reconstruct a supertree covering the core proteome. The best-fit model for amino acid replacement with respect to BIC was determined using IQ-TREE v2.0.3 (Nguyen et al., 2015). The reported model was JTT (Jones et al., 1992) with a FreeRate model (Yang, 1995) of 9 categories of rate heterogeneity across the site and empirical base frequencies, JTT+F+R9. It was used to reconstruct the phylogenetic tree, constrained by the topology of the species tree.

Next, GeneRax v2.0.4 (Morel et al., 2020) was used for a species-tree-aware Maximum Likelihood-based gene family tree inference using the UndatedDL reconciliation model (including speciation, duplication and loss events). 20 iterations were performed to reconcile the gene and species trees. This evolutionary reconstruction was then visualized using iTOL v6 (Letunic and Bork, 2021). It forms the foundation of the Kiwellin nomenclature.

### Nomenclature

Based on early duplication events, three major Kiwellin groups were identified Kwl1, Kwl2, and Kwl3. Kwl1 is closest to the lowest common ancestor node (LCA) that was predicted in the analysis and thus represents the primal Kiwellin subfamily. Kwl3 is the most recent subfamily. Within these major groups, the following duplication events with a representative number of species covered were used to further refine subgroups, e.g. Kwl1-1, Kwl1-2, and so on. Paralogs within species are then numbered (ascending by age/distance to LCA) using letters, e.g. Kwl1-1a, Kwl1-1b, and so on. A notable exception is Kwl3-1 which was grouped based on a speciation rather than a duplication event. However, this subfamily is specific to Liliopsida and was therefore threaded separately.

To ease reading and summarize our findings, species were matched to taxonomic groups according to the NCBI taxonomy (Sayers et al., 2021). These groups are referred to by a three-letter code. The abbreviations used here are as follows: Fungi (FUN), Bryophyta (BRY), Lycopodiopsida (LYC), Amborellales (AMB), Liliopsida (LIL), Ranunculales (RAN), Magnoliidae (MAG), Saxifragales (SAX), Rosids (ROS), Caryophyllales (CAR), and Asterids (AST).

### Consensus

Kiwellin sequences with trimmed signal peptides were grouped according to their respective major subfamilies (Kwl1, Kwl2, Kwl3, see ‘Nomenclature’ above). As most but not all Kwl1 Kiwellins contain a kissper domain, this subfamily was further divided in Kissper-Kwl1 and Kwl1 (without kissper). To emphasize the major differences, Barwin-like proteins identified through the Barwin-Model (see ‘Kiwellin annotation’ above) were added as an additional group. Note that this set is biased as it was constructed from false positive Kiwellin annotations to discriminate Barwin-like proteins from Kiwellins already at the sequence level. The groups were aligned using muscle v3.8.1551 (Edgar, 2004). Alignment columns with more than 90% gap content were dismissed, to reduce complexity.

A consensus sequence was calculated for each group and visualized using jalview v2 (Waterhouse et al., 2009). The conservation score of the Kiwellin consensus sequences was calculated following Livingstone and Barton (1993). 1 to 9 indicates property conservation of the alignment column in ascending order. Full property-related conservation is indicated by +, perfect amino acid conservation by *. Physico-chemical properties are highlighted based on the color schema provided by Larkin et al. (2007) (known e.g. from clustalX). The full alignments are available *via* our GitLab repository, see *Data Availability Statement*.

### Transcriptomics

Publicly available RNA-seq data sets in NCBI SRA (Sayers et al., 2021) were identified that were created to study either pathogenic or symbiotic interactions in at least two biological replicates. A complete list is compiled in the **Supplemental Data**. The amino acid sequences of the respective Kiwellins were mapped to the respective transcripts either based on the NCBI transcriptome (Sayers et al., 2021) or, if not available, based on the cDNA sequences provided by Ensembl Plants (Yates et al., 2022) using Proteinortho in autoblast mode (to match translated DNA/RNA with amino acid sequences) with a relaxed minimal sequence coverage of 20% and rather strict minimal percent identity of 90% and e-value threshold of 10^-50^, see **Supplementary Data 3**.

RNA-seq libraries were quality trimmed using trim_glalore v0.4.4 Krueger et al. (2021) and sequencing adapters were removed using cutadapt v2.3 (Martin, 2011). For paired-end sequenced experiments, the mate reads are omitted. Reads were mapped to the transcriptome of the plant under study and its pathogenic or symbiotic partner organism if available using bwa v0.7.17 Li (2013) with default parameters. The average number of mapped transcripts per data set can be found in **Supplementary Data 3**. It was not always possible to assign a read to a single transcript due to close sequence similarity. In these cases reads with multiple hits were accounted proportionate to the targets. However, we conservatively neglected reads that were mapped to a Kiwellin and a non-Kiwellin transcript to avoid linking measured expression of two or more genes between both groups.

Differential gene expression analysis was performed using DESeq2 v1.22.2 (Love et al., 2014). To reduce background noise, transcripts with less than 20 reads combined for all replicates and conditions were neglected. Technical replicates were collapsed using the collapseReplicates routine. For each dataset, we picked relevant replicates and conditions to compare control or mock-treated versus infected or treated in pairwise analyses (Wald test). A detailed listing is provided in the **Supplemental Datas 1, 3**. Transcripts with a baseMean (a proxy for overall expression strength) above 80 were considered highly expressed. Only significantly regulated Kiwellin transcripts with a P-value below 5% and an absolute log2-fold-change of at least 1 were considered for further evaluation.

## Results

### Structural characteristics of Kiwellins, Kissper-Kiwellins and Barwin-like proteins

To get insights into the Kiwellin family of proteins we first worked out the structural characteristics. Approaches used to identify Kiwellins so far did not include this parameter to separate this protein family from Barwin-like proteins. **Figure 1** highlights distinct structural features. Over 90% of the identified Kiwellins contain a signal peptide and thus can be secreted from the cell *via* the conventional pathway. The core of a Kiwellin protein is about 110 aa long. It consists of three *α*-helices and six parallel and antiparallel connected *β*-strands, that form a so-called double-psi *β*-barrel. This type of fold is also characteristic for the superfamily of Barwin-like proteins (**Figure 1B**). Two disulfide bonds between the two smallest *β*-strands *β*5 and *β*6 provide additional stability. The long flexible loop connecting *α*1 and *β*3 is fixed to the barrel by another disulfide bridge, which is anchored between *β*1 and *β*2.

In contrast to Barwin-like proteins, Kiwellins have an additional N-terminal extension of about 25 to 45 aa (**Figure 1B**). This domain consists of two *β*-strands connected by a loop. The loop region between *β3’* and *β4’* is highly variable. It is stabilized by disulfide bridges at both ends of the sheets that allow linkage of the extension with external loops of the *β*-barrel. Another disulfide bridge connects the loop located between the two *β*-strands that form the *β*-hairpin to the loop between *β*5 and *β*6, likely to connect this flexible and long loop to the core of the protein. We will refer to this N-terminal region as the Kiwellin-extension (compared to Barwin-like proteins).

The second class of Kiwellins, the so-called Kissper-Kiwellins (Ciardiello et al., 2008) include one further N-terminal extension of about 40 aa (**Figure 1C**). This domain is enriched in disulfide bridges and short regions of secondary structure elements (Ciardiello et al., 2008; Hamiaux et al., 2014). Notably, the loop connecting *β*3’and *β4’* is significantly shorter in Kissper-Kiwellins, it decreases from 15 aa in Kiwellins to only 2 aa in Kissper-Kiwellins. This changes the arrangement of disulfide bridges in the Kiwellin-extension. The shorter loop provokes the absence of disulfide bridge 5 found in other Kiwellins and influences anchoring of disulfide bridge 4 in Kiwellins (compare the disulfide-bound forming cysteine residues 4, 4’, and 5 in **Figures 1B** **, C**). Three disulfide bridges are formed in the Kiwellin extension to stabilize the small fold (compare cysteine residues 5’, 6, and 7).

### The evolution of Kiwellins

**Figure 2:**
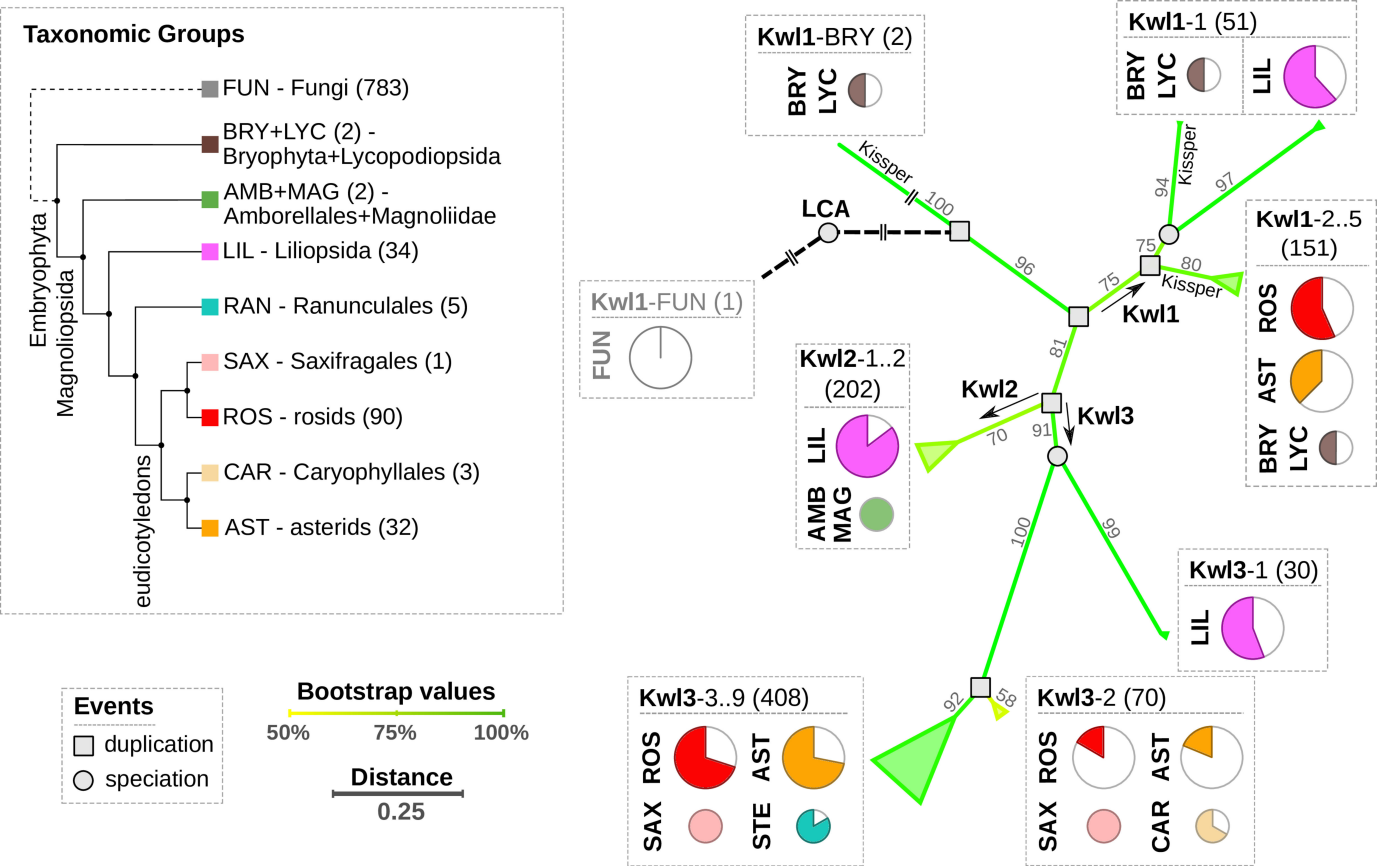
Left panel: Cladogram of the relevant taxonomic groups. The number of respective species in our data set is indicated in brackets. Right panel: Summary of the phylogenetically reconciled Kiwellin tree. The edge color and numbers refer to bootstrap percentages. Evolutionary events are indicated by a circle (speciation) or a square (duplication). Pie charts visualize the species coverage. Groups that mostly contain Kissper-Kiwellins are indicated (Kissper).

We reconstructed the phylogeny of Kiwellins and reconciled this data in the respective species tree to estimate duplication, speciation, and loss events along the evolution of this protein family. A summarized illustration is shown in **Figure 2**. The complete phylogenetic reconciliation including an annotation of duplication, speciation, and loss events can be found in **Supplemental Data 2**. The root of the tree was automatically estimated. It is located between the evolutionary oldest species in Bryophyta and Lycopodiopsida and the putative fungal prediction which we show here for the sake of completeness. We propose that Kiwellins close to the root represent the primal instances of this protein family.

Our analysis revealed three initial duplication events and we, thus, distinguish three major Kiwellin groups: Kwl1, Kwl2, and Kwl3 (see *Materials and methods* for details). Kwl1 is the most ancient group probably representing the original Kiwellin subfamily. It is present in most taxonomic groups and is enriched in evolutionary older groups. It is found in younger groups as well e.g. in rosids and asterids but was frequently lost. Out of 205 Kwl1 proteins, 137 contain a kissper domain. This domain is restricted to the Kwl1 subfamily. The enrichment of this additional domain in a specific group of plants was not observed. Notably, Kissper-containing Kiwellins are completely missing in LIL. Kiwellins with and without kissper domain are phylogenetically grouped next to each other. An alternative phylogenetic analysis based on Kiwellins with truncated kissper domains resulted in a comparable phylogeny (**Figure S1**), indicating that these longer sequences did not alter the phylogenetic reconstruction significantly.

The Kwl1 group spans 87 species including the oldest embryophyta *Physcomitrium patens* and *Selaginella moellendorffii* and five taxonomic groups BRY, LYC, LIL, ROS, and, AST. The Kwl2 subfamily contains 202 proteins and is restricted to a limited number of taxons. This subfamily is probably derived from a specific duplication found only in LIL, AMB, and MAG. Compared to the other groups LIL species predominantly contain Kwl2. Kwl3 is the largest group, comprising 508 Kiwellins in 115 species covering the six taxonomic groups ROS, AST, RAN, SAX, CAR, and LIL. For all taxonomic groups except LIL, Kwl3 is the overall youngest but also the most abundant subfamily.

### Sequence and structure conservation of Kiwellin subfamilies

**Figure 3:**
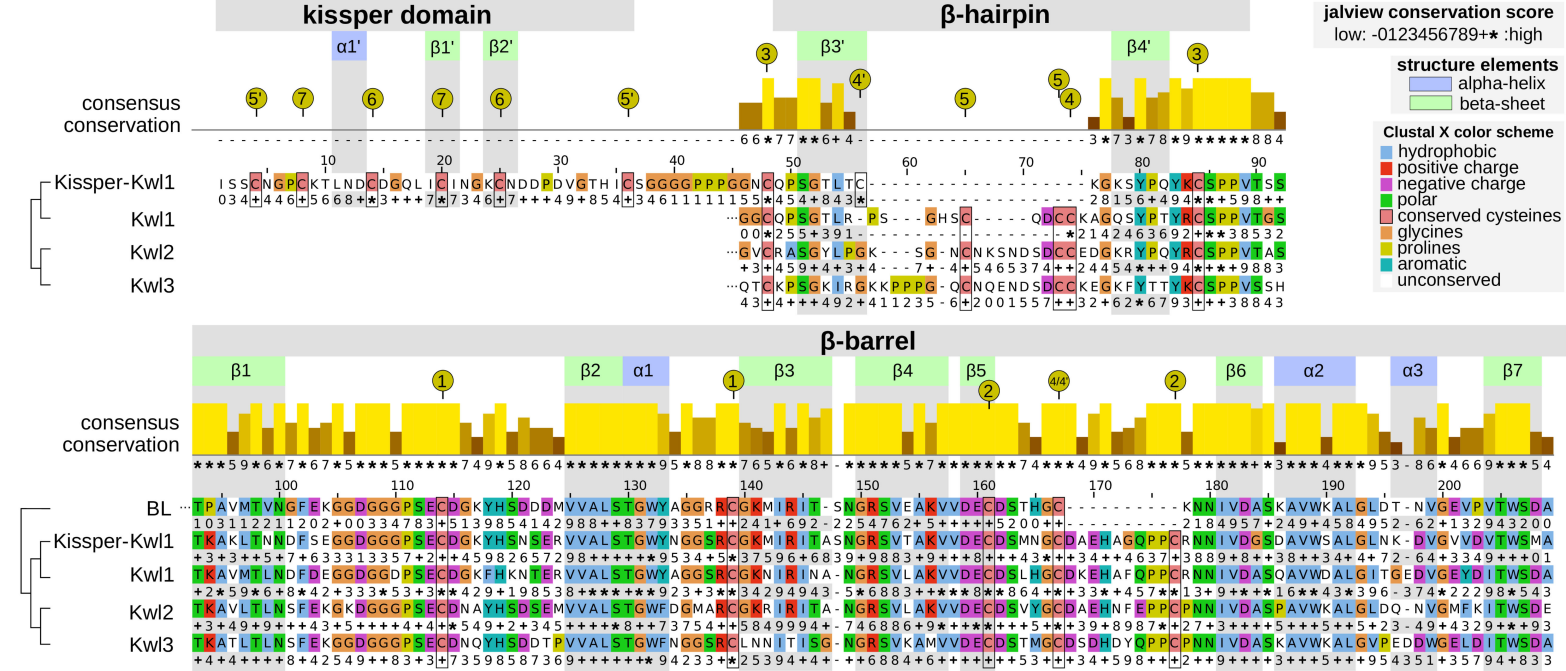
Aligned consensus sequences (without signal peptide) with secondary structure information of the Kiwellin subfamilies and a set of 391 BL proteins for reference. The conservation score of the consensus alignment for all Kiwellins is indicated above. The family-specific conservation is shown below the respective sequence: − (mostly gaps), 0…9, + (property conservation, ascending), ∗ (perfect conservation). Amino acids are colored according to their physicochemical properties. Positions and secondary structure elements were drawn corresponding to Kissper-Kwl1. Green represents β-sheets, blue α-helices. Numbered, yellow circles indicate the cysteine residues forming disulfide bounds.

**Figure 4:**
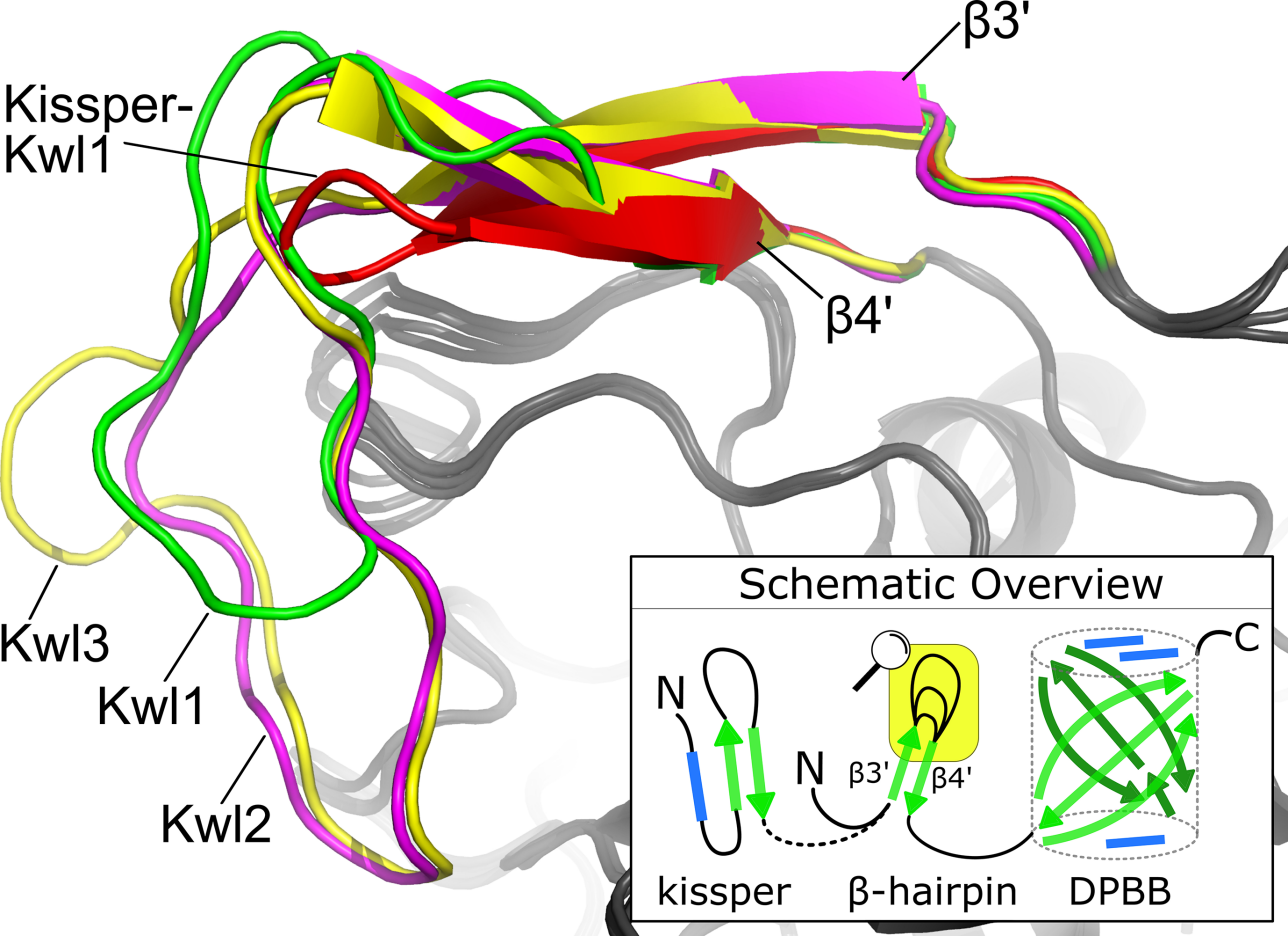
The consensus structure of the loop connecting β3′ and β4′ in the β-hairpin. The Kiwellin-groups are highlighted by different colors: red: Kissper-Kwl1, green: Kwl1, magenta: Kwl2, yellow: Kwl3. The bottom right: a schematic overview of (Kissper-)Kiwellins. Green arrow: β-sheet, blue rectangle: α-helix, yellow box: zoomed region.

The evolutionary reconciliation of Kiwellins identified three major subfamilies, Kwl1, Kwl2, and Kwl3. Kwl1 can be divided into two subgroups: one with and another without the kissper domain. **Figure 3** shows a sequence-based alignment based on consensus sequences. While Kiwellin sequences are highly conserved, the subfamilies can be discriminated by the length of the variable loop region between *β3’* and 4’ (**Figure 4**). In particular, this loop is short in Kissper-Kiwellins (median of only two amino acids). For Kwl1 this loop has a median length of 14, Kwl2 17, and Kwl3 20 amino acids. While cysteines are highly conserved, the disulfide bridge pattern differs between Kiwellins with and without the kissper domain since the loop region in the Kissper-Kiwellins is shortened and thus contains only one cysteine. For Kiwellins without the kissper domain, the loop is extended. Hence, a total of six cysteines are found that form three additional disulfide bridges. Overall, the loop appears to be a modular region with the lowest overall sequence conservation, e.g. significantly lower than the barrel-giving *β*-sheets (one-sided Wilcoxon rank-sum test, *p*<0.02). Notably, this region is not present at all in BLs. Similarly, the loop between *β5* and *β*6 is usually shorter in BLs. Both features make it possible to distinguish BL and Kiwellins. While this is sufficient in most cases, about one-third of the BLs contain a loop similar to Kiwellins (**Figure S2**). Notably, neither the length nor the sequence composition of this region did impact the structure predictions.

The amino acid sequences in the remaining secondary structure elements are strongly conserved. In particular, the *β-*sheets and the α-helices located in the barrel *β1–β*7 and *α1–α*3 are significantly conserved relative to the adjacent unstructured regions (one-sided Wilcoxon rank-sum test, *p*<0.05). Most of the amino acid positions in the secondary structure elements of the barrel are entirely conserved (*) especially *β*2, *α*1, *β*5 and *β*6. Less conservation was observed for other elements in the barrel. For example, *α*3, *β*3 and *β*7 have some positions with low conservation scores compared to the overall consensus, as well as in the individual subfamilies (e.g. position 1, 2 in *α*3 or 5 in *β*7).

Of interest, we identified 61 additional proteins containing a kiwellin domain as part of a larger protein distributed over 33 species with no clear taxonomic limitation. About half (30) of those proteins are considerably larger (three to four times), still, no further domain could be identified. 26 hits are duplications of the kiwellin domain (**Figures S3A, B**). In two cases, triplications were found (**Figure S3C**). Similar domain duplications were also found for Kissper-Kiwellins (**Figure S3D**). All identified fusion proteins are listed in **Supplementary Data 3**.

### Dissemination

**Figure 5:**
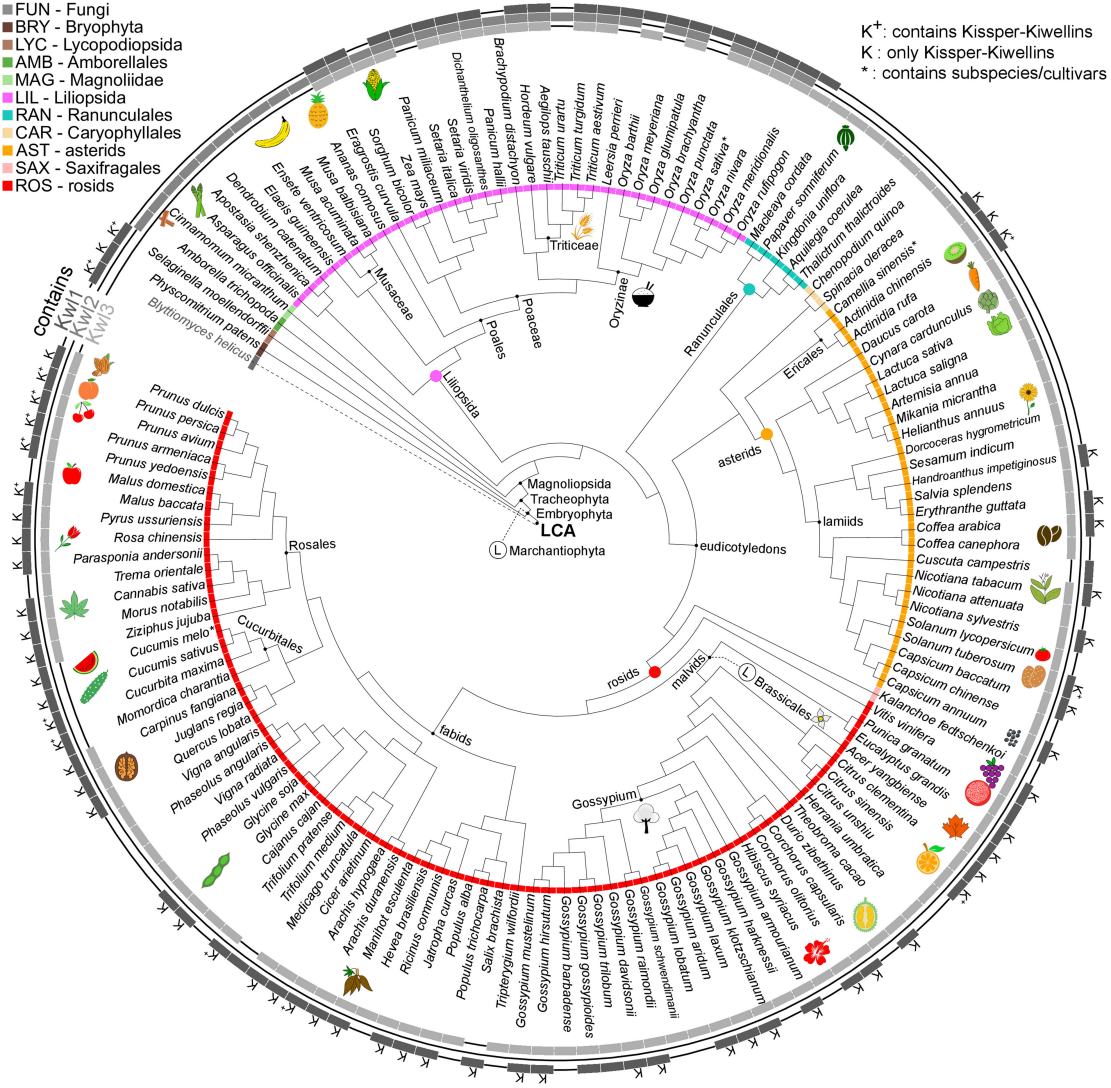
Species tree cladogram. The inner circle encodes taxonomic groups. The outer circle indicates if a species is found in the Kiwellin group Kwl1, Kwl2 or Kwl3. K^+^: Kwl1 contains Kissper-Kiwellins, K: only Kissper-Kwl1, *: contains subspecies/cultivars, Ⓛ: putative loss event.

A species-wise view of Kiwellin subfamilies (**Figure 5**) shows that Kwl2 is exclusively found in LIL where it is typically present, apart from some *Oriza* cultivars. LIL species on the other hand do not encode any Kissper-Kiwellins. We observed two major Kiwellin loss events coinciding with a loss of BLs in the order of Brassicales (e.g. *A. thaliana*) as well as the division of Marchantiophyta. The latter is only represented by two species in our data set. Thus, a general conclusion cannot be drawn at this stage. Brassicales are represented by thirteen species. Kiwellins are present in species sharing a common ancestor with this group (e.g. *E. grandis*). Therefore, a loss event is likely and in line with a reported whole genome triplication in the group, followed by many loss events in members of Brassicaceae (Moghe et al., 2014).

The Kiwellin subfamily Kwl2 is lost in the younger taxonomic groups of ROS and AST, while the evolutionary older Kwl1 including the Kissper-Kwl1 is still present. Thus, Kwl2 was probably lost in the intermediate group of LIL. A loss of the otherwise predominant Kiwellin subfamily Kwl3 is found in the order of Cucurbitales (ROS).

We observed a median of five Kiwellins in the larger taxonomic groups LIL, ROS, and AST, however, with a significant difference in numbers at the genus level and, strikingly, already at the level of breeds and cultivars. This can be explained by the degree of genome expansion. One measure for this feature is the unreplicated haploid nuclear genome amount also known as 1C-value (Soltis et al., 2013). Minor genome expansion is reported in CAR, SAX, and AST while large expanded genomes are found in some clades, especially within LIL which is found to show an exceptionally large range of 1C-values compared to the other taxonomic groups (Leitch et al., 1998). The reported 1C ranges of AST and ROS are similar (AST: 0.3-24.8pg, ROS: 0.1-16.5pg). This coincides with the number of Kiwellins occurring in these groups (AST: 1-24 and ROS: 1-17). SAX and CAR are reported to exhibit lower 1C ranges and as well contain a below-median number of Kiwellins.

The allotetraploid pasta wheat *T. turgidum*, one of the oldest domesticated crops, is known for its potential to obtain resistance to biotic and abiotic stresses. It encodes the second-highest number of Kiwellins (28). The bread wheat *T. aestivum* (LIL) has the highest number of Kiwellins observed in our data set (52). Most belong to the Kwl2 subfamily. Notably, *T. aestivum* is an allohexaploid composed of the three species *T. urartu*, *A. tauschii* and an unknown close relative to *A. speltoides* (not in this analysis) (Feldman and Levy, 2012). *T. urartu* and *A. tauschii* contain Kiwellins above the median and predominantly of type Kwl2. It is thus reasonable to assume that *T. aestivum* kept most of the Kiwellins from the donor species. In contrast, *T. urartu* harbors a diploid genome (Liu et al., 2017) and codes for nine Kiwellins.

### Screening and consolidation

**Figure 6:**
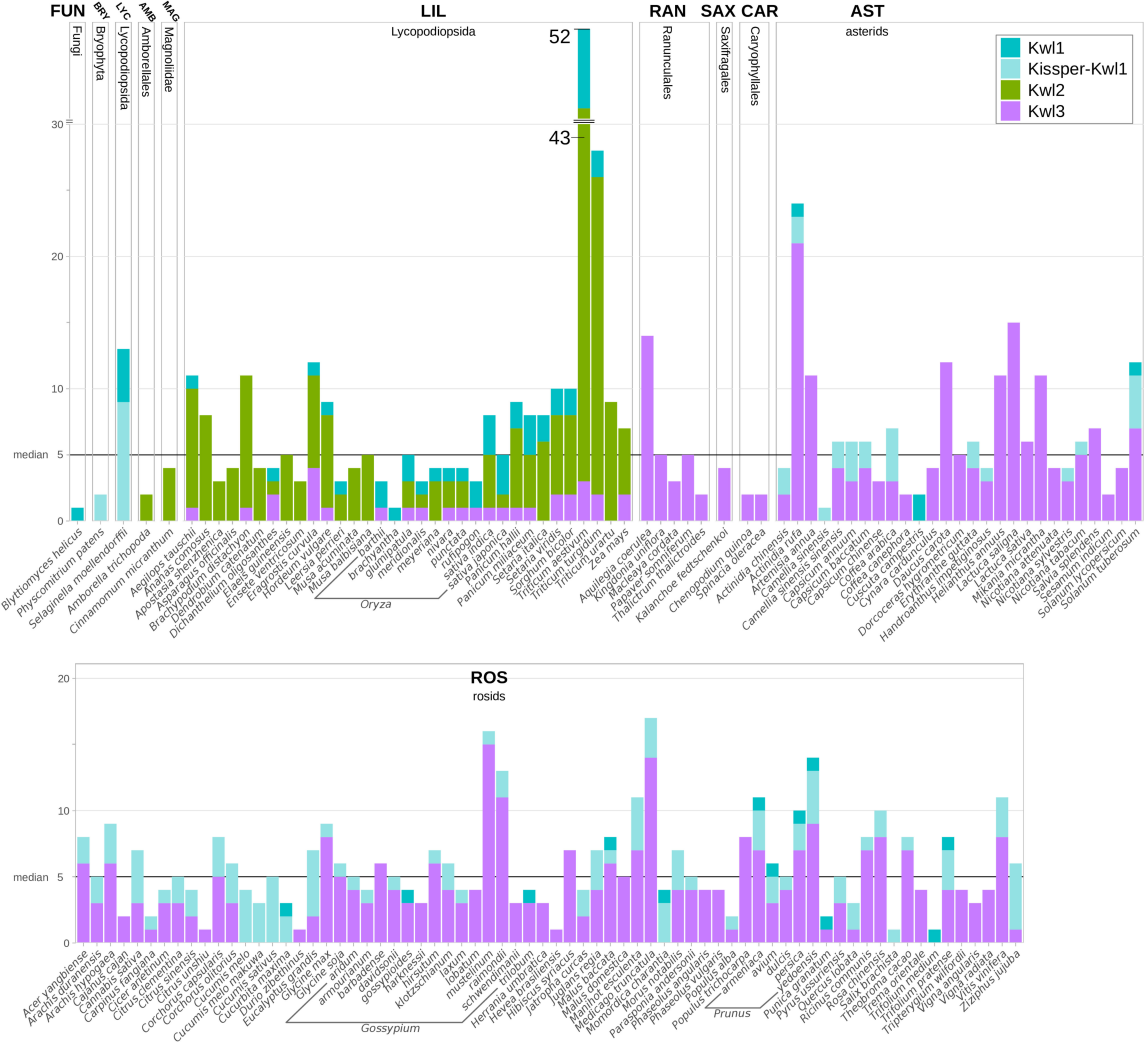
Kiwellins detected among specified species. The black horizontal line indicates the overall median of five Kiwellins per proteome. Colors indicate Kiwellin groups. Dark-turquoise: Kwl1, light-turquoise: Kissper-Kwl1, green: Kwl2, magenta: Kwl3.

A total of 20,630 full proteomes was screened for Kiwellins (see *Materials and methods* for details). None of the sequence-based predictions could be structurally verified in Bacteria, Archaea, or Viruses/Phages. Except for a single instance, no Kiwellins were found in the 783 fungal species in our data set. The single hit detected in Fungi is from *Blyttiomyces helicus* (A0A4P9WPM3), a saprophyte, which grows on pollen and cannot be cultured so far (Ahrendt et al., 2018). Given the phylogenetic position of this gene in our reconciliation, horizontal gene transfer from a plant or contamination is unlikely. The remaining 915 Kiwellins were identified in 142 land plants (embryophyta) (**Figure 6**).

The set of ‘Kiwellin-like’ proteins provided by InterPro (IPR039271) contains 2,362 entries that overlap to a large extent with our source data set of full proteomes (Blum et al., 2021). While it covers all Kiwellin entries identified here, it also contains many BL proteins and several unrelated proteins that we could not verify as Kiwellins. In total, we estimate only about half of the set to represent canonical Kiwellins. The published Kiwellin structure from *Actinidia chinensis* (PDB: 4PMK/Uniprot: P85261) corresponds to Kwl1-2a according to our nomenclature (Hamiaux et al., 2014). The crystal structures of *Zm*KWL1a (PDB: 6FPG/Uniprot: A0A1D6GNR3) and *Zm*KWL1b (PDB: 6TI2/Uniprot: K7U7F7) from *Zea mays* correspond to Kwl3-1b and Kwl2-2d (Han et al., 2019; Altegoer et al., 2020). *Zm*KWL4 (Uniprot: A0A1D6GNR6) corresponds to Kwl2-2e and *Zm*KWL6 as well as *Zm*KWL12 are identified as BL proteins. Similarly, a fungal rust effector protein that suppresses cell death in plants was found to be a BL protein as well (Jaswal et al., 2021). The structure from *Actinidia deliciosa* (PDB: 4X9U/Uniprot: P84527) was not recovered as the species is not part of the data set (Offermann et al., 2015). Nevertheless we identified this protein as a Kissper-Kwl1, orthologous to Kwl1-2a from *Actinidia chinensis*.

### Meta-analysis of Kiwellin expression in biotic and abiotic interactions of plants

Kiwellins have been identified as relevant defense proteins against the pathogenic fungus *U. maydis* (Han et al., 2019). Moreover, it has also been suggested that Kiwellins show tissue-specific expression patterns (Altegoer et al., 2020). To gain a broader view of how Kiwellin proteins are expressed in various situations, we performed a meta-analysis of publicly available transcriptome datasets from NCBI SRA. We focused on experiments in which Kiwellin-containing land plants were exposed to either pathogens or symbionts. Moreover, we only selected datasets, which were documented by a publication, comprising at least two biological replicates, contained unambiguous sample and experimental descriptions, and produced reliable FastQC scores (further details in **Supplementary Data 1** chapter 5). A total of 31 data sets (out of 70 initially selected) met these criteria (**Table 1**). 14 out of the 31 data sets showed strong expression levels, as well as significant changes in Kiwellin mRNA levels in response to interactions with symbiotic or pathogenic species. A detailed listing of individual findings and full experimental descriptions as well as the full workflow description is provided in **Supplement Data 1** chapter 5. Overall, our meta-analysis revealed that strongly expressed Kiwellins are present in each of the three subfamilies Kwl1, Kwl2, and Kwl3. About half of these Kiwellins show a significant response upon the interaction of the analyzed plant species with the respective interaction partner, being either a pathogen or a symbiont (**Table 1**). Specifically, we detected strong and significant Kiwellin responses in 7 out of the 21 data sets analyzing a plant-pathogen interaction (see **Figure S7**). For the interaction of a plant with its symbiont, we have also detected strong expression levels and regulation of Kiwellin transcripts, however, only in 5 out of 15 experiments (see **Figure S7**). These findings might indicate a role of the Kiwellin in the interaction of plants with the cognate pathogens and symbionts as well. Taken together, our meta-analysis supports the idea that Kiwellins represent a regulatory layer in the plant-microbe interactions, although additional experiments are required to further consolidate this notion. We would also like to note that we observed a tissue-specific expression difference in three distinct species *M. truncatula*, *M. acuminata* and *T. aestivum* in roots compared to leaves and one in nodules compared to roots. Notably, in wheat Kwl2 and Kwl3 are enriched in roots, while Kwl1 seems to be more prevalent in leaves. These findings are in line with the previously recognized tissue-specific expression patterns of the maize Kiwellins (Altegoer et al., 2020). Altogether, our observations might suggest that the differential expression of Kiwellins in tissues is a general feature of land plants. Moreover, we also observed that Kiwellins can be upregulated during water limitation, which might suggest that also abiotic factors are able to induce a Kiwellin response (Riahi et al., 2019).

**Table 1:** Overview of strongly expressed and significantly regulated Kiwellin groups among all species with RNA-seq data sets.

Taxonomic Group		Species	Kwl1^K^	Kwl1	Kwl2	Kwl3	#/total
Bryophyta (BRY)		*P. patens*	P↑*	–	–	–	1*/1
Liliopsida (LIL)		*M. acuminata*	–	–	S↑ T	–	2/3
		*Z. mays*	–	–	P↓*	P↓* P↑ S↑	2(+1*)/ 5
		*T. aestivum*	–	P↓ S↓ T	P↕ S↕ T	S↑ T	3/4
		*O. sativa*	–	P↕	Ø	Ø	1/2
asterids (AST)		*S. lycopersicum*	–	–	–	S↑	1/3
		*S. tuberosum*	Ø	Ø		Ø	0/1
		*A. chinensis*	P↑*	–	–	Ø	1*/1
Caryophyllales (CAR)		*C. quinoa*	–	–	–	Ø	0/1
rosids (ROS)		*G. max*	P↑ S↕^*^ T*	–	–	Ø	1(+1*)/5
		*C. sativus*	S↑ P↑	–	–	–	2/2
		*M. truncatula*	Ø	–	–	S↑ A↑ T	2/2
		*C. melo*	Ø	–	–	–	0/1
							Σ 14 (+4*)/31

Kwl1^K^: Kissper-Kwl1, P: pathogenic interaction, S: symbiotic interaction, T: tissue-specific, A: abiotic stress. −: Kiwellin group not present in this species, ∅: no Kiwellin found with significant regulation, ↑: up-regulated, ↓: down-regulated, ↕: up and down-regulated (compared to the respective control), *: weakly expressed but with significant differences, #: number of independently collected data sets with significantly regulated and strongly expressed Kiwellins.

## Discussion

Plants have developed numerous strategies to cope with abiotic and biotic stresses caused by e.g. drought or viral, bacterial, fungal, or herbivore pathogens (Draffehn et al., 2013; Quintana-Camargo et al., 2015; Mosquera et al., 2016). Kiwellin encoding genes have been shown to be regulated upon these challenges e.g. (Huang et al., 2017; Fiorilli et al., 2018; Lanver et al., 2018). Recent studies demonstrated that two Kiwellins from maize specifically target a secreted effector protein from *Ustilago maydis* (Han et al., 2019; Altegoer et al., 2020) making this protein family an prominent new candidate to better understand plant-microbe interactions.

Our study introduces a unified nomenclature and Kiwellin phylogeny to guide future research on Kiwellin proteins. Combining structural predictions and sequence-based analysis we can clearly distinguish between Kiwellins and BL proteins. Interestingly, Kiwellins appear to be a unique invention of land plants despite one singular hit found in the fungal kingdom. Further investigation will be required to understand the evolution of this putative Kiwellin. Our structural comparison suggests that Kiwellins are derivatives of BL proteins. Starting from the BL fold, Kiwellins may have evolved, for example, by extending the N-terminus, which may serve as a surface extension of the protein to perform specific functions.

Another feature present in many Kiwellin proteins is the kissper domain. Out of 915 Kiwellins identified in our study, 143 proteins harbor a kissper domain. It was shown in experiments that the *Actinida deliciosa* Kissper-Kiwellin can be cleaved into two domains kissper and kiwellin by actinidain, a cysteine protease highly abundant in kiwifruits *in vitro* (Tuppo et al., 2008). Structural comparisons have shown that the short kissper peptide has high similarities to cysteine-rich motifs such as the epidermal-growth-factor-like motif, or toxins from animals (Ciardiello et al., 2008). This region, which is only 40 amino acids long, contains 6 cysteines and can form 3 disulfide bridges. Remarkably, the kissper peptide exhibits pH-dependent and voltage-gated ion channel-forming activity in synthetic lipid bilayers (Ciardiello et al., 2008; Meleleo et al., 2012; Ciacci et al., 2014). The biological role of the kissper domain has not been elucidated. Suitable model systems to study the potential role of Kissper-Kiwellins for the interaction of plants with pathogenic or symbiotic microbes could be *Cucumis sativus* and *Glycine max*. Both are established model systems.

All three Kiwellin groups show significant responses as well as strong expression upon the interaction of plants with microbes. Therefore, we speculate that members of all Kiwellin classes may function as modulators of biotic interactions. Hence, manipulation of Kiwellins or Kiwellin expression might provide a novel means to develop new disease-resistant plants or plants with improved symbiotic capabilities e.g. for nitrogen-fixing bacteria. Suezawa et al. (2017) investigated the performance (a.o. photosynthetic rate, fruit quality, crop yield) of *A. chinensis* under poor drainage grafted on rootstocks of different *Actinidia* species. The best results were observed with *A. rufa* rootstocks, in which Kiwellins are highly abundant. Furthermore, Kisaki et al. (2019) showed increased tolerance to bacterial blossom blight in a hybrid breed of *A. chinensis* and *A. rufa*. This coincides with the difference in Kiwellins abundance between *A. rufa* (24 proteins) and *A. chinensis* (4 proteins).

## Conclusion

Kiwellins have distinct structural characteristics that need to be addressed when annotating new proteins of this family. Otherwise especially BL proteins are likely to be missannotated as Kiwellins as shown in examples from literature and a protein database. In addition, we identified three evolutionary distinct subfamilies that can be distinguished a.o. based on the length of the Kiwellin loop at the *β-*hairpin. We hypothesize that Kiwellins are evolutionarily derived from BL proteins that belong to the pathogen-related family 4. They may have additional functions in plant immune response due to the N-terminal extensions. The provided nomenclature and grouping of Kiwellins along with evidence from transcriptomic data indicating Kiwellin proteins as mediators of plant-microbe interactions will aid to guide further research in the fields of plant-pathogen and -symbiont interactions.

## Data availability statement

Publicly available datasets were analyzed in this study. This data can be found here: https://www.ncbi.nlm.nih.gov/sra IDS: PRJEB4211, PRJNA10698, PRJNA116, PRJNA122, PRJNA14007, PRJNA17973, PRJNA182750, PRJNA190909, PRJNA20061, PRJNA20263, PRJNA207554, PRJNA225997, PRJNA225998, PRJNA232045, PRJNA232125, PRJNA238126, PRJNA240798, PRJNA241430, PRJNA243, PRJNA245122, PRJNA246165, PRJNA257217, PRJNA261643, PRJNA262552, PRJNA262907, PRJNA263939, PRJNA268357, PRJNA268358, PRJNA28131, PRJNA282644, PRJNA285087, PRJNA29019, PRJNA293435, PRJNA29797, PRJNA301363, PRJNA315994, PRJNA316327, PRJNA319578, PRJNA319678, PRJNA326436, PRJNA328963, PRJNA33471, PRJNA341501, PRJNA342685, PRJNA342701, PRJNA34677, PRJNA350852, PRJNA355166, PRJNA371634, PRJNA376605, PRJNA376608, PRJNA38691, PRJNA389730, PRJNA394209, PRJNA394242, PRJNA394253, PRJNA395588, PRJNA396054, PRJNA396063, PRJNA397875, PRJNA407962, PRJNA418295, PRJNA432228, PRJNA438537, PRJNA453230, PRJNA453787, PRJNA471752, PRJNA476953, PRJNA482138, PRJNA48389, PRJNA492326, PRJNA49677, PRJNA50439, PRJNA506972, PRJNA524157, PRJNA525136, PRJNA534520, PRJNA560384, PRJNA574457, PRJNA576248, PRJNA580467, PRJNA631757, PRJNA633601, PRJNA638679, PRJNA655717, PRJNA66163, PRJNA673911, PRJNA689611, PRJNA691360, PRJNA698663, PRJNA702515, PRJNA702529, PRJNA713846, PRJNA737421, PRJNA74771, PRJNA764258, PRJNA774802, PRJNA796348. The tools and data sets (tables, sequences, structure predictions) generated for this study can be found in the uni marburg gitlab repository (https://gitlab.uni-marburg.de/synmikro/ag-lechner/kiwellins).

## Author contributions

GB conceived the study. ML supervised the project and drafted the manuscript. PK carried out the bioinformatic analyses and collected public RNA-seq data sets. PK, MC, and FA evaluated and verified structures. GB, ML, JF, and FA revised the manuscript. All authors wrote, read, and approved the final manuscript.

## Supplementary material

Supplementary Data Sheet 1 Additional figures and detailed algorithmic description (PDF). chapter 1, S1 Gene tree with and without truncated Kissper-Kiwellins. chapter 2, S2 Weblogo of consensus sequences. chapter 3, S3 Examples of kiwellin domain duplications and triplications. chapter 4 Detailed description of the kiwellin identification pipeline. S4 RMSDPMAS examples. S5 Range of descriptors used in find_kwl. S6 Kiwellin identification pipeline. chapter 5 Detailed RNA-seq results. S7 Workflow for re-evaluated RNA-seq data.

Supplementary Data Sheet 2 Fully reconciled Kiwellin evolution (PDF).

Supplementary Data Sheet 3 Listings, e.g. proteomes, RNA-seq sources, Kiwellin annotations (XLSX).
