# Supplementary material for: Evolutionary reconstruction, nomenclature and functional meta-analysis of the Kiwellin protein family
Source: Front Plant Sci. 2022 Dec 22;13:1034708. doi: 10.3389/fpls.2022.1034708 (PMC9813671; doi:10.3389/fpls.2022.1034708)
Supplement: Supplementary Data Sheet 2 — Fully reconciled Kiwellin evolution (PDF). [file DataSheet_1.pdf]

Supplementary Data

1 GENE TREE WITH AND WITHOUT TRUNCATED KISSPER-KIWELLINS

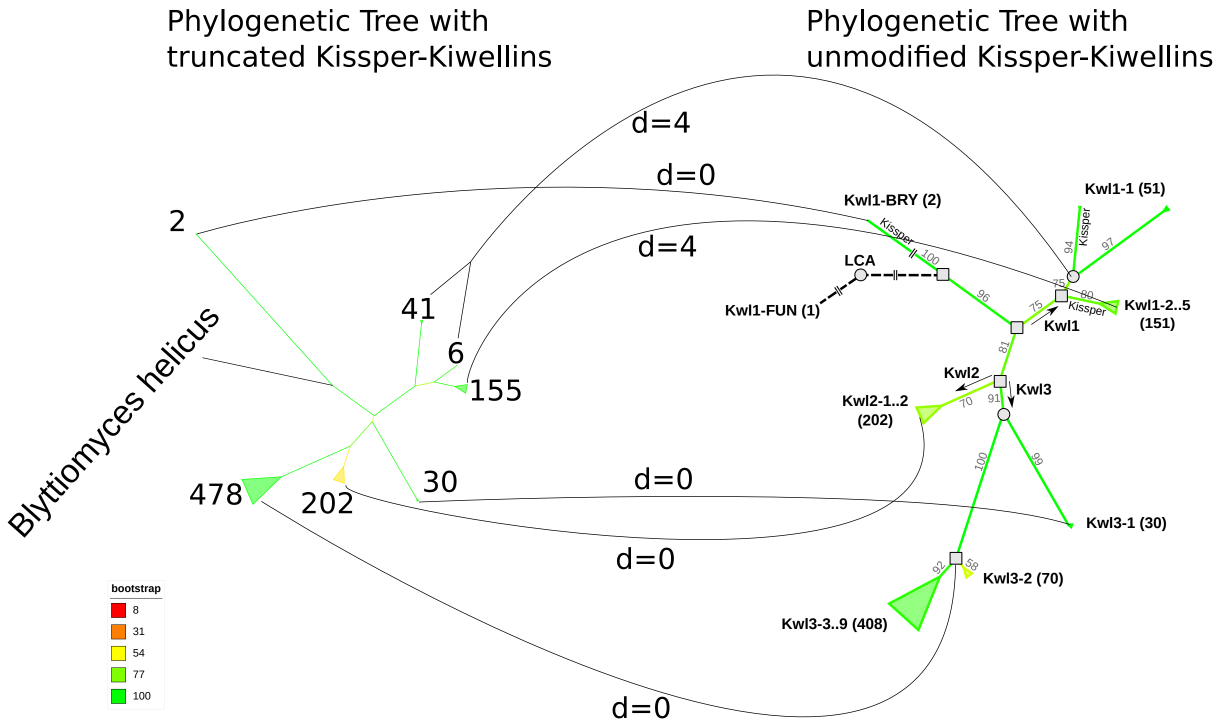

Figure S1: Left: phylogenetic tree with truncated Kissper-domains. The text on the collapsed branches indicates the number of proteins. Right: Kiwellin phylogenetic tree of Fig. 2. The text above the black lines indicates the number of differences between the connected sub-trees.

2 WEBLOGO OF CONSENSUS SEQUENCES

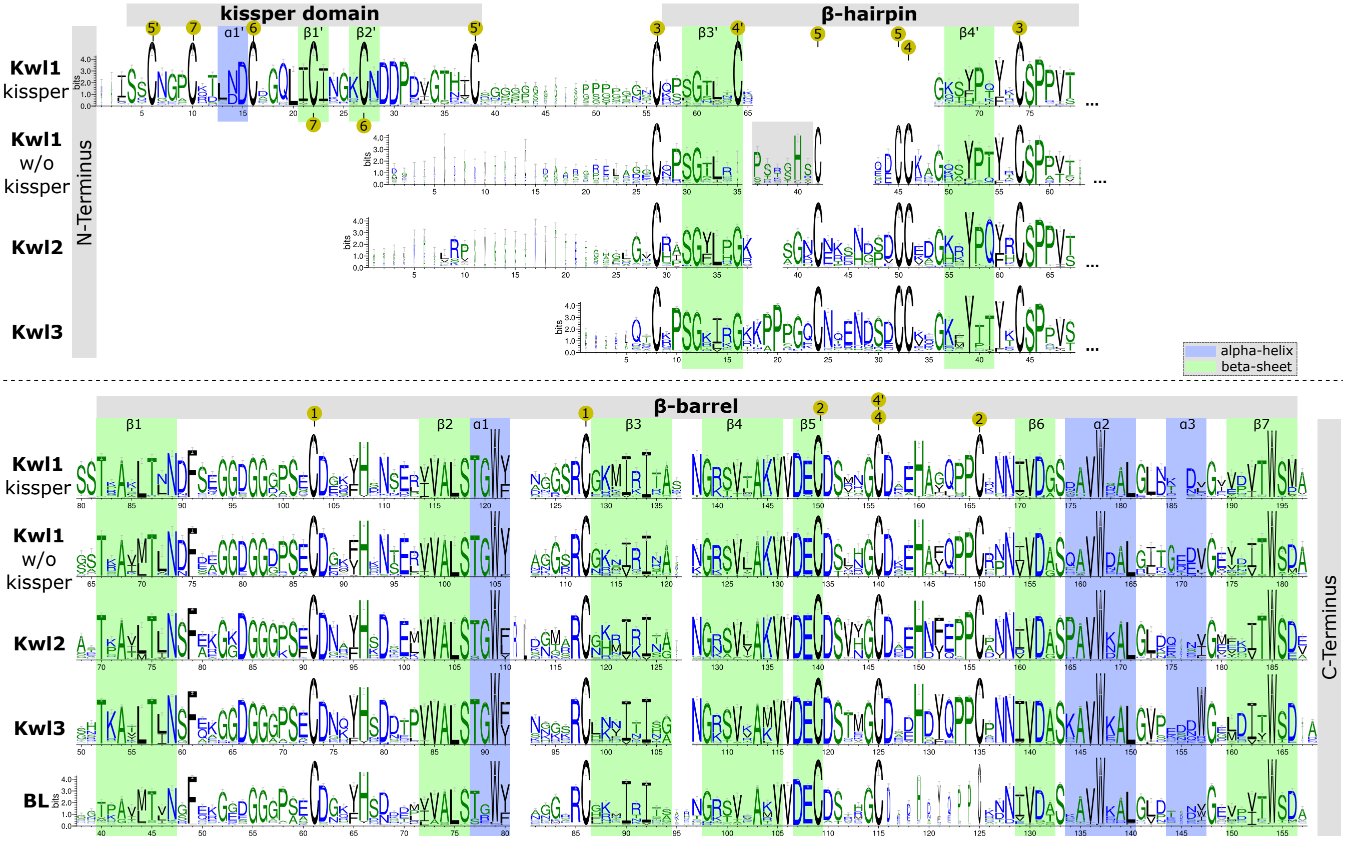

Figure S2: Weblogo of aligned consensus sequences (signal peptide trimmed) with secondary structure information of the Kiwellin groups Kissper-Kwl1, Kwl1 (Kiwellins without Kissper domain), Kwl2, and Kwl3 and a set of 391 BL proteins for reference. Green represents beta-sheets and blue alpha-helices. Numbered, yellow circles specify the cysteine residues forming disulfide bounds.

3 EXAMPLES OF KIWELLIN-DOMAIN-DUPLICATIONS AND -TRIPLICATIONS

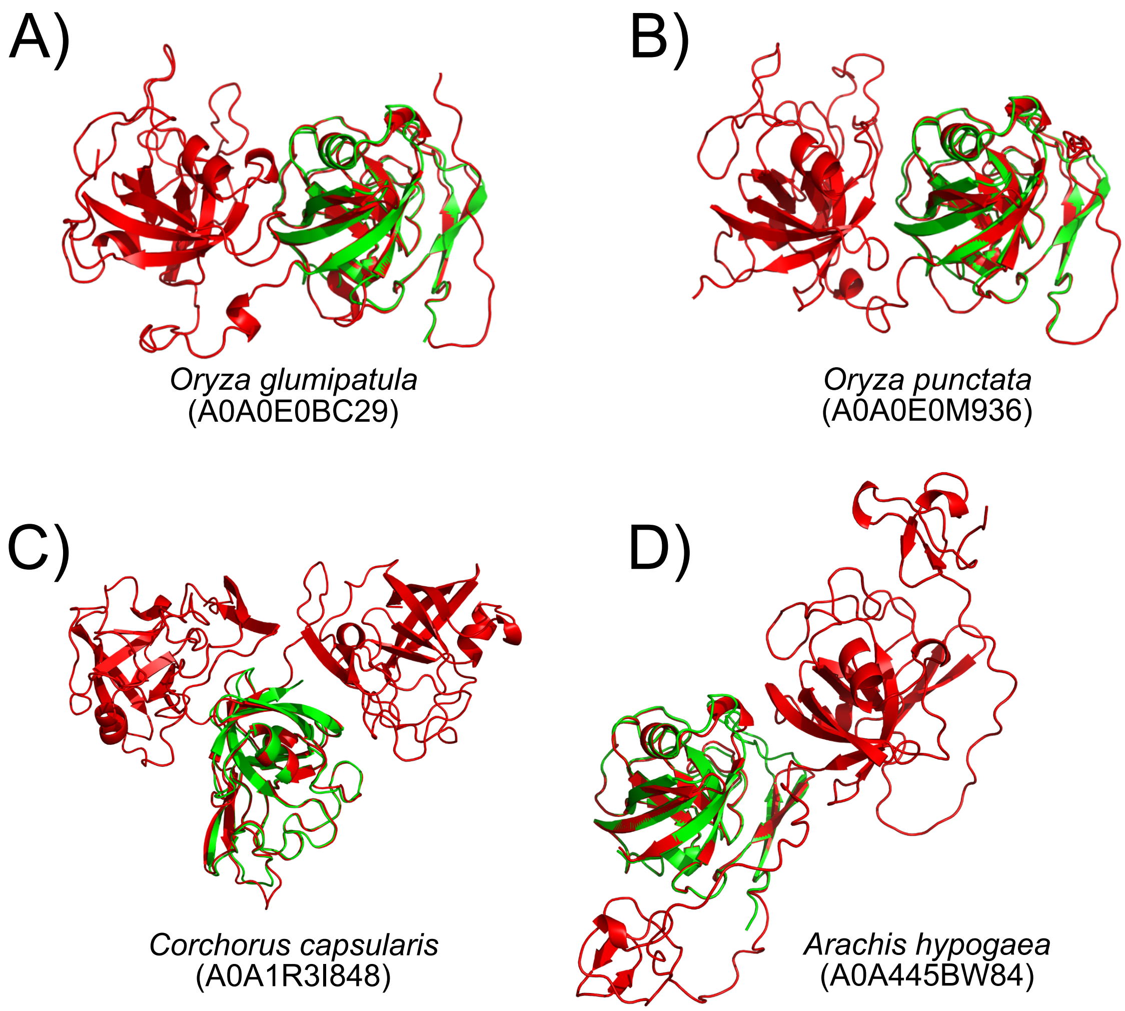

Figure S3: Examples of proteins were found for Kiwellins using the algorithm with relaxed length parameters. A and B show Kiwellin-domain-duplications, C shows a triplication, and D shows a domain-duplication of Kissper-Kiwellins.

4 DETAILED DESCRIPTION OF THE KIWELLIN IDENTIFICATION PIPELINE

4.1 Re-identification ($\texttt{round}_1$)

Han et al. (2019) published 620 putative Kiwellin proteins in plants and fungi in a total of 61 species covering seven taxonomic groups, namely non-seed plants (NSP), gymnosperms (GYM), monocots (MON), stem eudicots (STE), asterids (AST), rosids (ROS) and fungi (FUN). These sequences were used as queries for the `UniProt` reference proteomes set release `Reference_Proteomes_2022_01` (Consortium, 2019). To cover all taxonomic groups, incomplete proteomes were added in accordance with the species covered in Han et al. (2019) (a total of 20.9k proteins were added).

All proteins of (Han et al., 2019) were initially mapped to the respective `UniProt` proteome using `Proteinortho` v6.1.1 (Lechner et al., 2011) with an E-Value threshold of 10^{-50}. Ultimately 411

of the initial 620 proteins could be re-identified in the current release of `UniProt`. Next `SignalP` v5.0b (Almagro Armenteros et al., 2019) was used to predict and trim a leading signal peptide. If no signal peptide was found the leading residues were removed one by one until either a signal peptide was found or less than 50% of the original protein is left. If no signal peptide was found the protein is left unmodified. `Alphafold` v2.0.0 (Jumper et al., 2020) was used to predict the 3D structure using the database bfd_database bfd_metaclust_clu_complete_id30_c90_final_seq, mgnify_database mgy_clusters_2018_12 and references pdb70, uniclust30_2018_08 and uniref90. Finally `pyMOL` v2.5.2 (DeLano and Bromberg, 2004) was used for manual inspection and visualization.

In the following, we refer to the manually verified set of the 235 Kiwellins, 117 BL, and 59 unrelated proteins as $\texttt{round}_1$ and e.g. with $\texttt{round}_1^{\text{kissper}}$ the set of Kissper-Kiwellins of round 1.

4.2 Advanced search ($\texttt{round}_2$)

In the following, we frequently investigated if a certain descriptor falls into a range defined by $\texttt{round}_1$. To allow more atypical values, we introduced a tolerance parameter of 25% to soften cutoffs, which is used throughout this pipeline.

Building on the knowledge from $\texttt{round}_1$ we scanned the UniProt database once more with a sophisticated pipeline named `find_kwl`. This tool can be subdivided into 3 main steps:

1. Pre-filtering and Pre-processing
2. Collect descriptors
3. `hmmsearch` and filtering

4.2.1 Pre-filtering and Pre-processing

To reduce the search space and save computation time we filtered proteins first by sequence length. The signal peptide trimmed Kiwellins identified in $\texttt{round}_1$ contain between 150 and 227 amino acids (Kiwellins with and without Kissper domain) and at least 3 cysteine residues (including the BL proteins). To account for a possible signal peptide the length limit is extended by 90. Therefore, only proteins of lengths $150 - 317 \pm 25\%$ and at least 3 cysteine residues were initially considered. Those sequences were then trimmed using `SignalP` as described for the sequences of $\texttt{round}_1$. The trimmed sequences were filtered again by length: $150 - 227 \pm 25\%$.

4.2.2 Collect descriptors

Besides the sequence length and the number of cysteine residues, we wanted to evaluate the 3D structure. Using `AlphaFold` 3D structure predictions were generated for all proteins passing the pre-filter. As `AlphaFold` predictions do not include secondary structure, the `dssp` routine of the R package `bio3d` v2.4-1.9 (Grant et al., 2006) was used to define the number of continuous region of β-sheets (`b_regions`). E.g. the Kiwellin 3-1b (A0A1D6GNR3):

primary: `FPYRSLLQTCQPSGSIQGRSGNCNTENGSECCKNGRRYTTYGCSPPVTGSTRAVLTL`
secondary: `-------------BBBB--------AAA--------BBBB----------BBBBBBB`
primary: `NSFAEGGDGGGAAACTGKFYDDSKKVVALSTGWYNGGSRCRKHIMIHAGNGNSVSAL`
secondary: `------------------------BBBBBAAAA--------BBBBBB----BBBBB`
primary: `VVDECDSTVGCDKDHNFEPPCRNNIVDGSPAVWDALGLNKDDGQAQITWSDE`
secondary: `BBBBB-------------------BBBB-AAAAAAA---AAA-BBBBBBBB-`
removed signal peptide precursor: `MATVGGNRALYAVVALPLLATLLHGPMRLSHA`
`B`:β-sheet, `A`:α-helix, `-`:unstructured, $\texttt{b\_regions}:8$

Furthermore, we rated the 3D structure with a special focus on the different domains (barrel, kissper, clamp) of the putative Kiwellins. Thus, a set of reference structures from different species of $\texttt{round}_1$ was hand curated, i.e. multiple reference structures were used to combat a possible underfitting:

- 4 kissper domains: Kwl1-2b (A0A2R6PEY1), Kwl0-1a (A0A2K1KL29), Kwl1-5b (A0A251NR03), Kwl1-2a (A0A067F280)
- 5 clamp domains Kwl3-8b (A0A2R6RCR6), Kwl1-3c (M1AEA5), Kwl2-2i (T1MCJ8), Kwl2-2a (A0A1Z5RFC7), Kwl1-1a (D8S9G1) and
- 4 barrel domains: Kwl3-4d (M0ZG50), Kwl3-4e (M0ZG49), Kwl3-1b (A0A1D6GNR3), Kwl3-6a (A0A0R0KPS2)
- *Zm*KWL1a (A0A1D6GNR3) crystal structure from Han et al. (2019)

The extracted structures for the kissper and clamp domain were reduced to the first 60 residues and the one for the barrel domain to the 6 β-sheets $\beta 1, ...\beta 6$.

The structure prediction was superimposed using `PyMOL` with the set of references to calculate the `RMSD` (the lower the better) and the number of matching atoms (`MA`, the higher the better). The `RMSD` can be arbitrarily small even for unrelated proteins with short overlaps (low `MA`) and a low `RMSD` does not necessarily follow from a high number of matching atoms. Therefore we combined both values and define

$$\texttt{RMSDPMAS} := \frac{\texttt{RMSD}}{\texttt{MA}^2}$$

The lower `RMSDPMAS` the better the superimposition as shown in Fig. S4. With that, the smallest `RMSDPMAS` was determined for each set of reference domains (later referred to as kissper, clamp, and barrel `RMSDPMAS`). To ensure comparability with the kissper and clamp domain only the leading 60 residues of a protein were compared to the reference structures. As shown in Fig. S5 this leads to a reliable identification of the Kiwellins with and without a kissper domain using the kissper `RMSDPMAS` and the clamp `RMSDPMAS` respectively. Additionally, the barrel and *Zm*KWL1a `RMSDPMAS` were used to exclude the unrelated proteins.

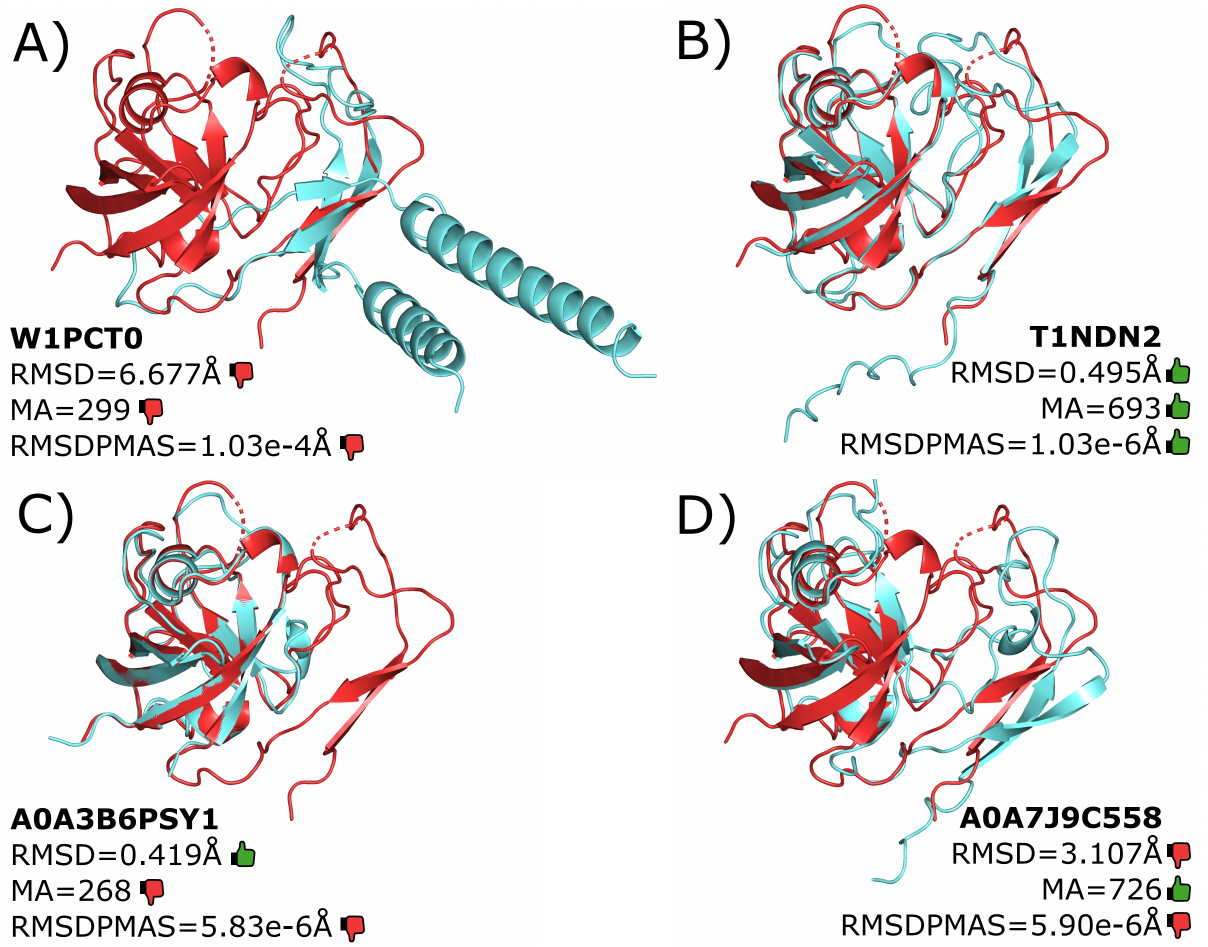

Figure S4: Superimposition of Kwl3-1b (A0A1D6GNR3, crystal structure of Han et al. (2019)) in red and blue the proteins A) W1PCT0, B) Kwl2-2c (T1NDN2), C) A0A3B6PSY1 and D) A0A7J9C558. A) shows an alignment with a high `RMSD` and low `MA` values resulting in a high `RMSDPMAS`. In contrast that the very similar structures of B) result in a low `RMSDPMAS`. In comparison the non-optimal alignments of C (short overlap with high similarity) and D (long overlap with low similarity) with similar `RMSD` or `MA` values, respectively both result in a higher `RMSDPMAS`.

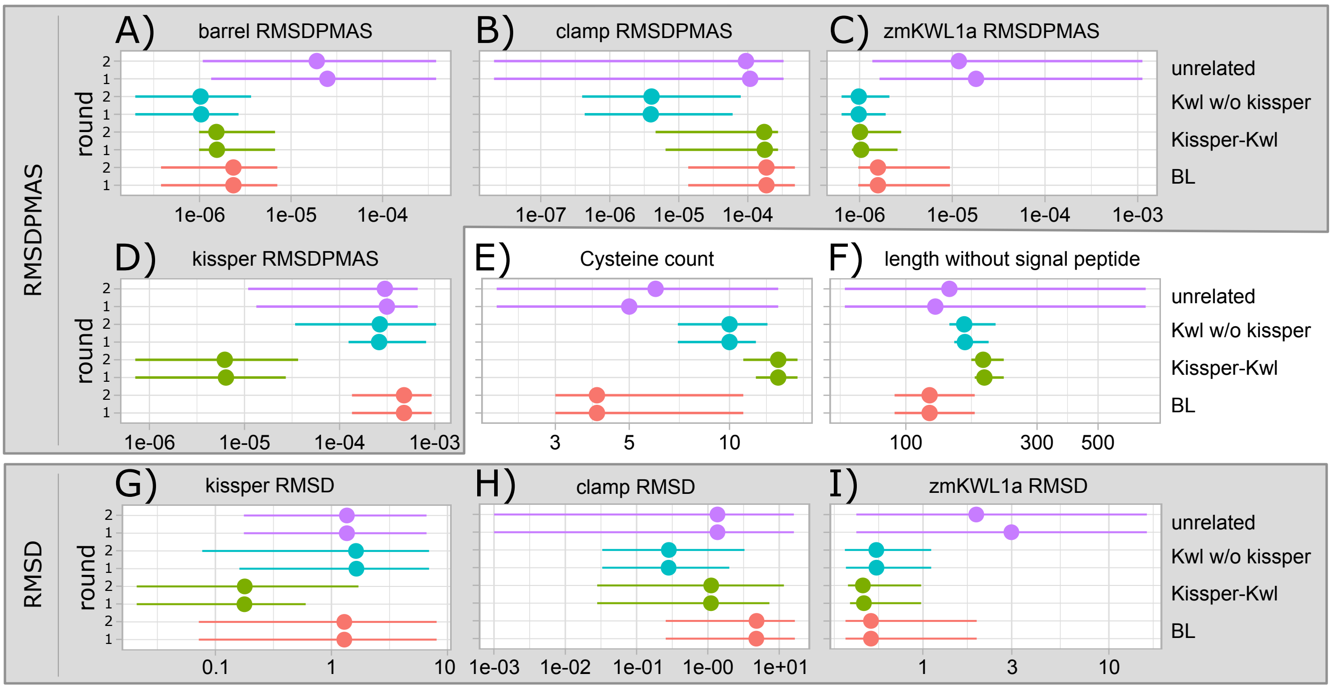

Figure S5: Min-Max range of the different descriptors used in `find_kwl` (A-F) as described in 4.2.2. Dots indicate the median value. G-I show some raw `RMSD` used to compute the `RMSDPMAS` of B-D. *Zm*KWL1a refers to the published crystal structure of A0A1D6GNR3 (Han et al., 2019).

Although the Kissper-Kiwellins contain a clamp domain the clamp `RMSDPMAS` is usually orders of magnitudes worse than the Kiwellins without the kissper domain since the first 60 residues do usually not contain the clamp.

To summarize, we collect the following descriptors for any protein that passes the pre-filter checks of 4.2.1:

- length of sequence after signalp trimming
- number of cysteine residues
- the number of continuous regions of β-sheets `b_regions`
- the structure scores `RMSDPMAS` against the set of reference structures

4.2.3 `hmmsearch` and filter

For each of the curated sets of $\texttt{round}_1$ (Kiwellins, Kissper-Kiwellins, and BLs) first an alignment was generated using a `muscle`. Next, those columns were removed that almost only consisted of gaps ($> 90\%$), and a hidden Markov model (HMM) was assembled using HMMer v3.2.1 (Eddy, 1998). `hmmsearch` was used to query the 3 models against all reference proteomes of UniProt. The resulting E-value of a match (m, p) between a model m and a protein p is denoted by $E_{(m,p)}$. To determine a suitable E-value cutoff for each model the 59 as unrelated identified proteins of $\texttt{round}_1$ were used as a negative control set:

$$c_m := \min_{up:\texttt{unrelated protein}} E_{(m,up)} = \begin{cases} 5.2 \cdot 10^{-42}, m = \texttt{BL} \\ 3.8 \cdot 10^{-54}, m = \texttt{kissper} \\ 3.2 \cdot 10^{-56}, m = \texttt{kiwelllin} \end{cases}$$

Next, we wanted to assess if a descriptor (e.g. number of cysteine residues) is untypical compared to the values of $\texttt{round}_1$ with a predefined tolerance parameter of $t = 25\%$. For that we will say that a descriptor *is part of* $\texttt{round}_1$ if the value v lies in the range $[\mathrm{mi}, \mathrm{ma}]$ of values of $\texttt{round}_1$ extended by the tolerance parameter t:

$$\overbrace{\mathrm{mi} - (\mathrm{ma} - \mathrm{mi}) \cdot t \leq v}^{\textit{at least}} \underbrace{\leq \mathrm{ma} + (\mathrm{ma} - \mathrm{mi}) \cdot t}_{\textit{below}}$$

On the same note we defined that a descriptor is *at least* or *below* $\texttt{round}_1$ for only the left or right inequality respectively.

Furthermore, we defined a set of filtered matches as the subset of all reported matches (`hmmsearch`) that fulfill the following set of rules:

- the E-value below the defined model specific cutoff: $E_{(m,p)} \leq c_m$
- the number of cysteine residues *at least* $\texttt{round}_1^m$
- the sequence length is *part of* $\texttt{round}_1^m$
- for $m =$ Kiwellin (without kissper domain):
  - the clamp `RMSDPMAS` is smaller than the kissper `RMSDPMAS` up to the tolerance $t = 25\%$
  - the clamp, barrel and *Zm*KWL1a `RMSDPMAS` is *below* $\texttt{round}_1^m$
  - if the protein length is below the midpoint of the lengths of $\texttt{round}_1^m$ then $\texttt{b\_regions} \geq 2$
- for $m =$ kissper (Kiwellin with kissper domain):
  - the kissper `RMSDPMAS` is smaller than the clamp `RMSDPMAS` up to the tolerance t
  - the kissper, barrel and *Zm*KWL1a `RMSDPMAS` is *below* $\texttt{round}_1^m$
- for $m =$ BL (Barwin-like):
  - the barrel and *Zm*KWL1a `RMSDPMAS` is *below* $\texttt{round}_1^m$

For each protein among the set of filtered hits, we report the model with minimal E-value as the best match for that protein.

In total 683 new Kiwellins were found, i.e. 59 with and 589 without a kissper domain. The steps $2 - 3$ of the pipeline were repeated with the new extended set of Kiwellins as the input (alignment, HMM model, descriptor ranges), and 15 further Kiwellins were identified. In a final step, this set of Kiwellins was checked by hand again and almost all entries (98.2%) could be verified as correctly classified. We removed only 17 entries. Most of which were faulty Kiwellins (missing $\beta 7$). A final set of 915 Kiwellins (62 with and 772 without a kissper domain) were reported and are denoted as $\texttt{round}_2$ in Fig. S5.

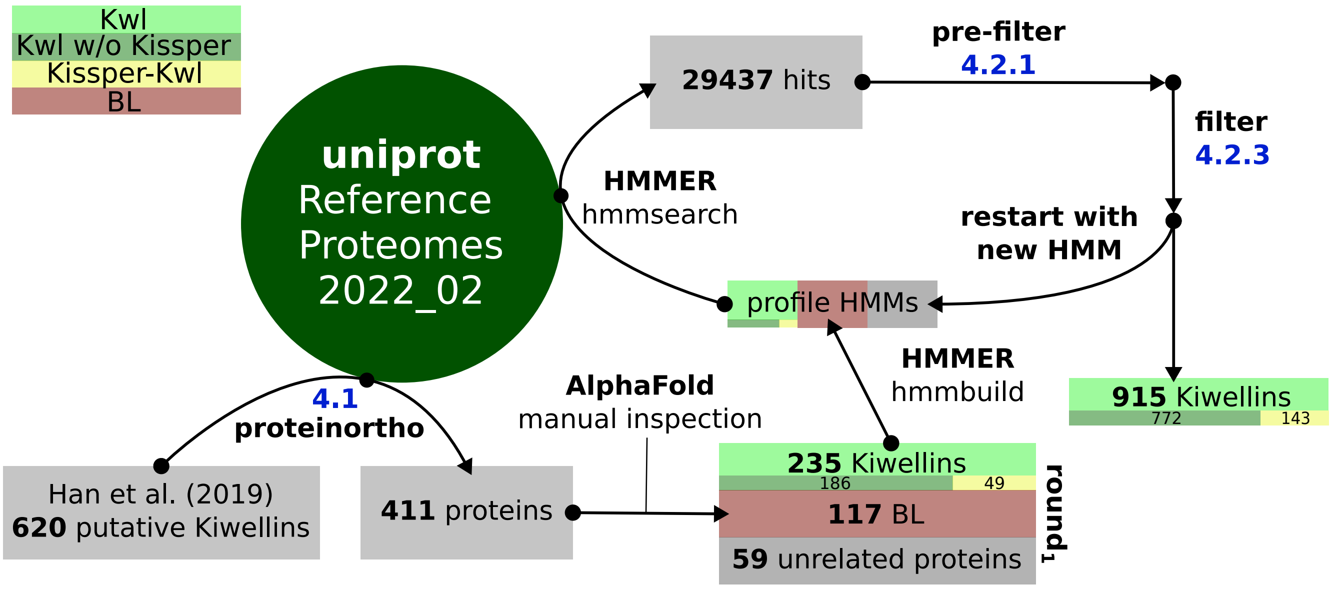

Figure S6: Flow chart of the Kiwellin identification pipeline. More details on the blue-marked processing steps are described in the respective chapters.

5 DETAILED RNA-SEQ RESULTS

To get an idea of the functions of Kiwellins we re-analyzed publicly available RNA-seq data sets from the NCBI SRA. 70 experiments were obtained using the following filter characteristics: RNAseq, RNA, stress keywords (pathogenic, symbiotic, water, ...), and the scientific name of the plants. The 70 data sets were checked on quality parameters of the raw data using fastQC (Andrews et al., 2010) and on data integrity (at least 2 replicates, associated publication, unambiguous sample naming, and experimental descriptions) resulting in 31 data sets. To determine if a Kiwellin was significantly regulated an FDR threshold of 5% was used. We consider an entry to be differentially expressed if the absolute log2 transformed fold changes (L2FC) is above 1 and the P-value is below the above FDR threshold. Furthermore, we define a Kiwellin group as strongly expressed if the baseMean (baseM) is at least 80 (a proxy for the overall expression strength; $\log_{10}(80)\approx 1.9$). Finally, we grouped the experiments by experimental parameters (pathogenic, symbiotic, abiotic, tissue-specific responses).

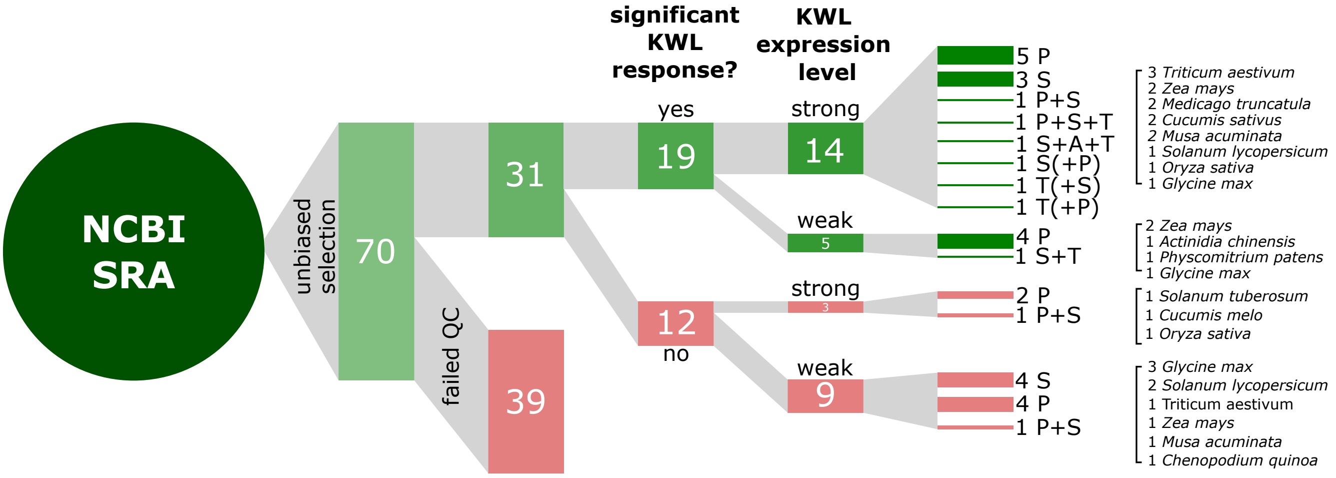

Figure S7: Sankey diagramm illustrates from left to right the workflow for re-evaluated RNA-seq data. P: pathogenic, S: symbiotic, A: abiotic, T: tissue specific responses. QC: Quality check. More information can be found in the chapter 5. The number of analysed experiments per species are given on the right side. Brackets indicate non signifiant interactions not significant (e.g. S(+P): significant response only to the symbiotic and not to pathogenic partner).

In the following chapters, we shortly describe the 31 analyzed case studies and examine the regulation of the Kiwellins groups. Groups were formed from indistinguishable Kiwellins concerning the associated transcript(s) as described in the Material and Methods. A Kiwellin group can include multiple proteins as well as transcripts. For example lets consider the kiwellin group 'Kwl2-2t,2v,2s' of *T. aestivum* shown in PRJNA743515. This group includes the three almost identical Kiwellin proteins Kwl2-2t, Kwl2-2v, Kwl2-2, that share 188/197 identical residues. With the help of proteinortho two similar transcripts (XM_044541154.1, XM_044500936.1) were identified from the respective transcriptome. Since the proteins as well as the transcripts are almost indistinguishable, we combine the results of this group into one entry. All identified groups of transcripts are listed in the table "Nomenclature-KWL" and the used transcriptomes in "transcriptome sources" of Supplementary Data 3 (proteinortho transcripts).

For each experiment, a heatmap is shown to visualize the L2FC on the left panel from blue (down-regulated) to red (up-regulated). Gray is shown if the comparison does not exhibit significant changes. The

middle panel specifies the log10 transformed baseM. A * symbol in the name indicates that the Kiwellin group surpasses the baseMean threshold of 80 and thus is considered to be strongly expressed. More details can be found in the Material and Methods. The right panel shows average normalized counts as well as standard deviations between the replicates of all conditions.

The results of the 31 analyzed experiments were divided into sections according to Fig. S7:

5.1 Significant response and strong expression 11
- 5.1.1 Pathogenic response: P 11
- 5.1.2 Symbiotic response: S 14
- 5.1.3 Pathogenic but no symbiotic response: P+S 16
- 5.1.4 Pathogenic, symbiotic and tissue specific effect: P+S+T 17
- 5.1.5 Symbiotic and abiotic response and tissue-specific effects: S+A+T 17
- 5.1.6 Symbiotic but no pathogenic response: S(+P) 18
- 5.1.7 Tissue specific but no symbiotic response: T(+S) 19
- 5.1.8 Tissue specific but no pathogenic response: T(+P) 19

5.2 Significant response and weak expression 20
- 5.2.1 Pathogenic response: P 20
- 5.2.2 Pathogenic and tissue specific response: S+T 22

5.3 Strong expression but no significant response 22
- 5.3.1 Pathogenic response: P 22
- 5.3.2 Pathogenic and symbiotic response: P+S 23

5.4 Weak expression and no significant response 24
- 5.4.1 Symbiotic response: S 24
- 5.4.2 Pathogenic response: P 26
- 5.4.3 Pathogenic and symbiotic response: P+S 28

5.1 Significant response and strong expression

5.1.1 Pathogenic response: P

Triticum aestivum (PRJNA743515)

Bipolaris sorokiniana is a hemibiotrophic fungus responsible for several plant diseases. The study Zhang et al. (2022) aimed to investigate how genes are regulated when *Triticum aestivum* is infected by pathogenic fungus (TAB). Uninfected plants (TA) served as control groups. Plants were soil-inoculated and samples of root and basal stems were harvested 5 and 15 days after infection. RNA was isolated from the samples and sequenced.

We found 4 strongly expressed groups (*-prefix), three from Kwl1 and one from Kwl3, and 6 further weak expressed groups of Kwl2. We found that one group of the weakly expressed Kwl2 to be differentially regulated 5 dpi (3 L2FC). One group of Kwl1 showed a slight down-regulation late in the infection stage (-1.2 L2FC) and remained unchanged at 5 dpi.

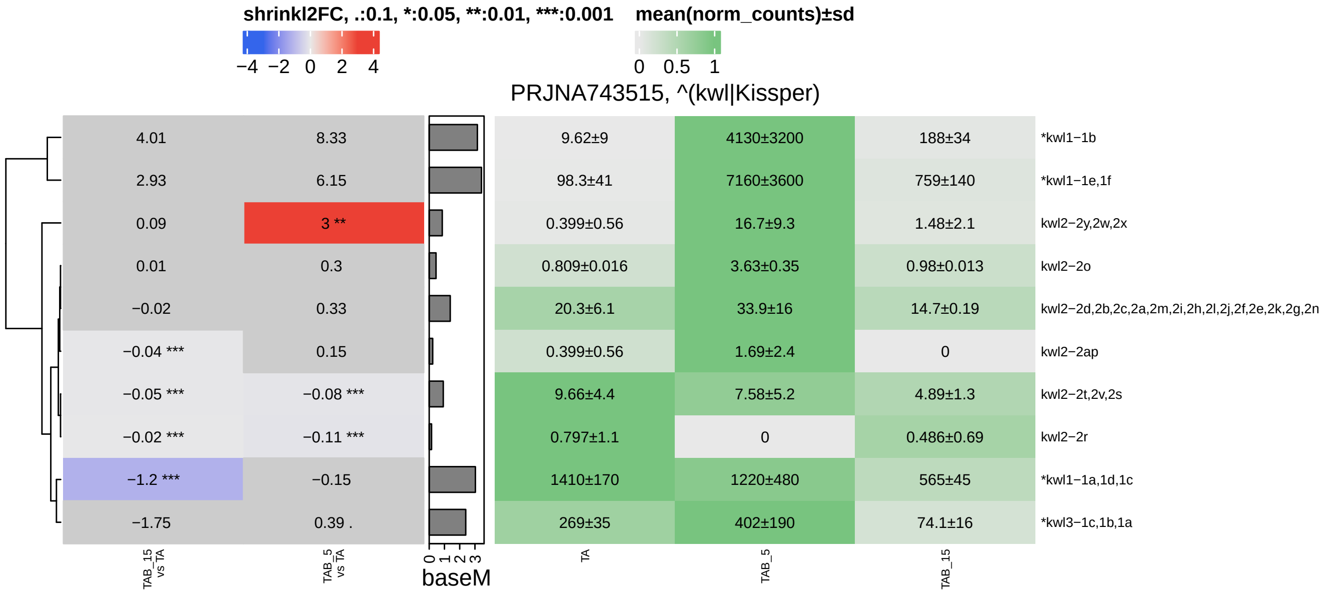

Zea mays (PRJNA407369)

Zea mays was syringe-infected with *Ustilago maydis*. In Lanver et al. (2018) infected plant material was harvested at different time points ($\frac{1}{2}$, 1, 2, 4, 6, 8, 12 dpi) and mRNA was analyzed. Axenic *U. maydis* culture and water-inoculated plants (mock) served as controls or comparison groups.

We found 2 Kiwellins of Kwl3 and Kwl2, of which Kwl3-1b is strongly expressed (*-prefix). Furthermore, Kwl3-1b showed a strong up-regulation ($\approx 5 - 7$ L2FC) among all time points compared to the mock-inoculated control. The weakly expressed Kwl2 protein showed a late response with a slight up-regulation ($\approx 1 - 3$ L2FC) starting at 6 dpi.

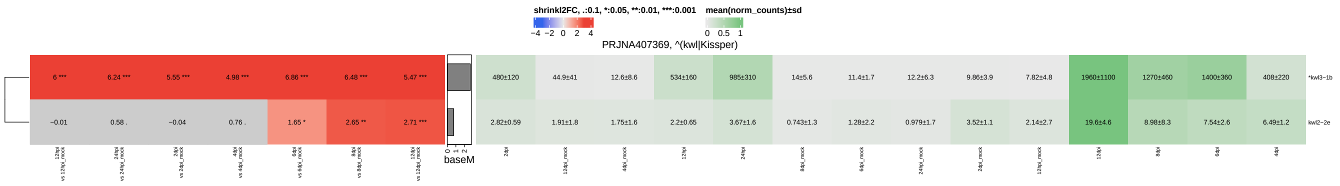

Oryza sativa (PRJNA325291)

In Huang et al. (2017), rice was inoculated with the fungus *Magnaporthe oryzae* (with=Guy, without=Before). It is known that nitrogen fertilization increases the effects of many diseases. The authors studies whether the external addition or omission of nitrogen led to differentially expressed genes during infection in both species to explain Nitrogen-Induced Susceptibility (NIS). For this purpose, rice plants were infected with water or the fungus and 0 dpi or 2 dpi shoot tissue of the plants were harvested. Nitrogen was omitted from the fertilizer in one series of experiments (0N) and added in the form of ammonium nitrate in another (1N). Subsequently, mRNA was isolated from the obtained tissue and analyzed.

We found 2 groups of two Kwl1 and one Kwl3 to be highly expressed. The kwl3 group shows no regulation in response to the infection. One Kwl1 group was down-regulated (4 L2FC), and in the second group, a slight up-regulation upon infection under nitrate treatment was found. No ammonium nitrate-specific response was observed among all groups.

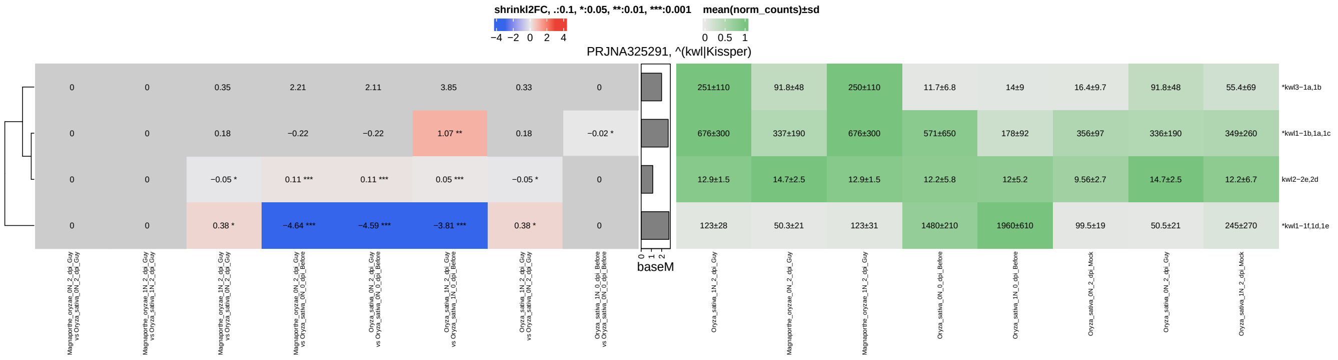

Cucumis sativus (PRJNA285071)

In Burkhardt and Day (2016), a resistant strain (PI197088) and an susceptible strain (Vlaspik) of *Cucumis sativus* were infected with the fungus *Pseudoperonospora cubensis* and water (mock), respectively. Leaves of the plant were harvested 1, 2, 3, 4, and 6 dpi and mRNA levels were determined in each case.

We found a group of Kissper-Kiwellins (Kwl1) to be strongly expressed in the susceptible strain and moderately in the resistant strain. Furthermore, we found a strong up-regulation upon infection ($\approx 4-6$ L2FC) in the susceptible strain throughout infection, while the resistant strain showed a slight dampening of the differential response ($\approx 3-4$ L2FC). Furthermore, the response vanishes at 6 hpi for the resistant strain.

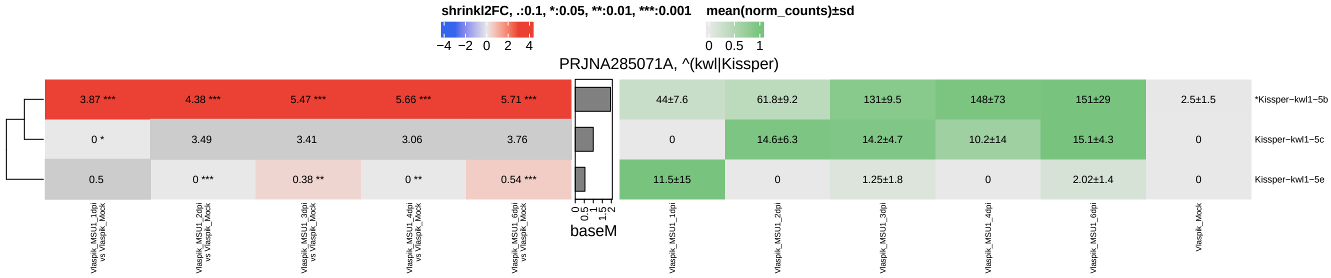

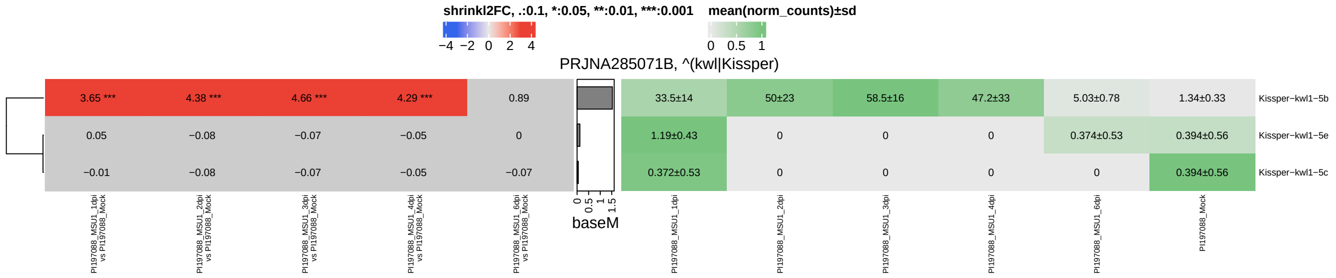

Glycine max (PRJNA412201)

It is known that silicon can protect plants from biotrophic and hemibiotrophic pathogens. To better understand this mechanism, Glycine max was infected with *Phytophthora sojae* in Rasoolizadeh et al. (2018). Silicon was added in one case (SiPlus) and omitted (SiMinus) in the other plants. After 21 days of infection, root samples were collected and mRNA was isolated and sequenced.

We found one group of Kissper-Kiwellins (Kwl1) to be highly expressed with a differential response to the infection ($\approx 1-3$ L2FC). The silicon treatment slightly reduced effect (≈ 1 L2FC less).

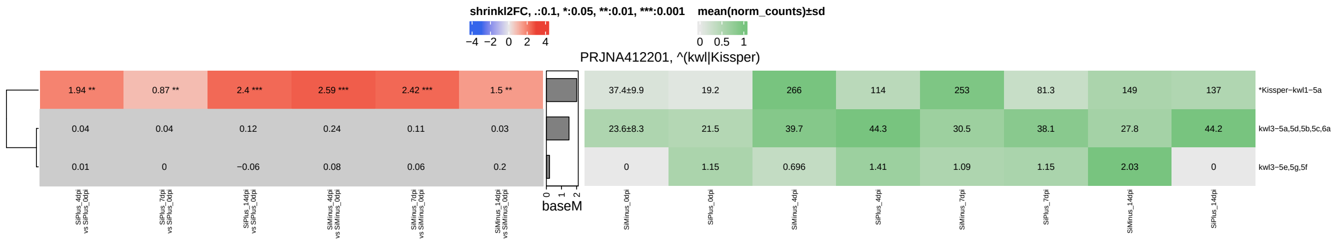

5.1.2 Symbiotic response: S

Musa acuminata (PRJNA319058)

In Gamez et al. (2019) seedlings of Musa acuminata were inoculated with two species of growth-promoting rhizobacteria: *Bacillus amyloliquefaciens* (Ba) and *Pseudomonas fluorescens* (Pf). 1 hpi, 2 dpi and 4 dpi whole seedlings were collected, and the mRNAs were isolated. These data sets were compared with water-inoculated seedlings.

We detected three Kiwellins groups of Kwl2. Kwl2-1b was the only strong expressed group and showed a weak up-regulation upon infection with Pf after 1 hpi and remains inconspicuous otherwise. The other two Kiwellins showed no differential response to the infection.

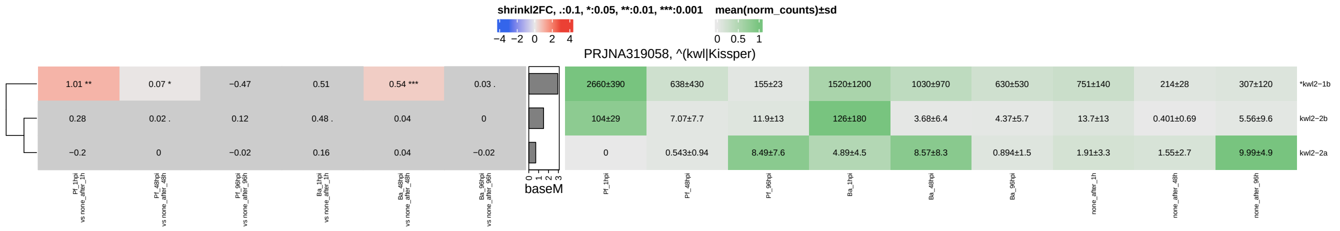

Triticum aestivum (PRJNA529884)

In Li et al. (2018a) *Triticum aestivum* was infected with the arbuscular mycorrhizal fungus *Rhizophagus irregularis*. After 42 days of infection, shoot tissues of the plants were harvested and mRNA was extracted and analyzed. This data set was compared with non-infected plants.

Our analysis revealed 4 groups (Kwl1, Kwl3, and 2 Kwl2 variants) of highly expressed Kiwellins. Members of Kwl2 and Kwl3 showed a strong up-regulation upon infection ($\approx 3 - 8$ L2FC) and Kwl1 a down-regulation (≈ 2 L2FC).

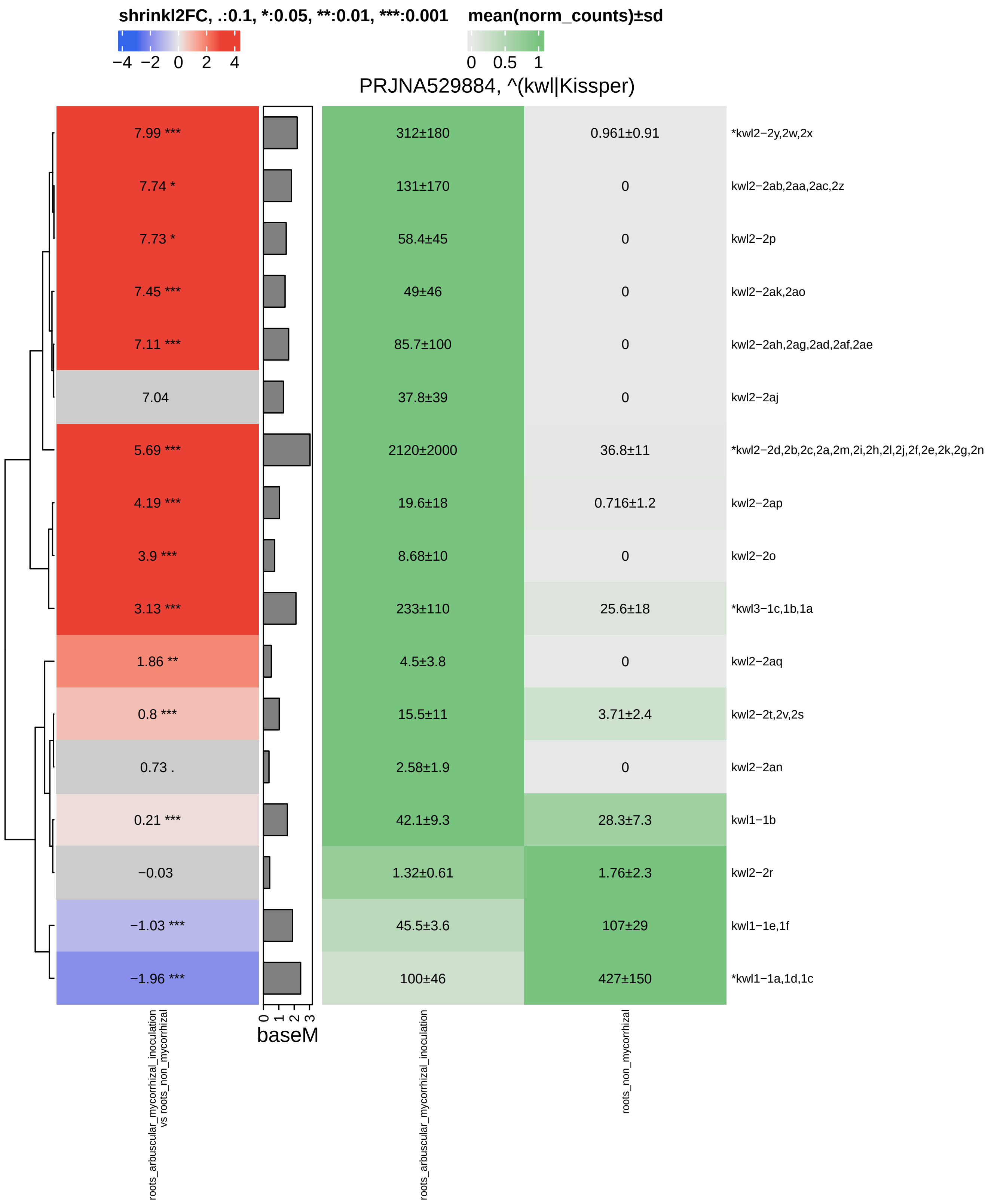

Zea mays (PRJNA506746)

In Shen et al. (2020), the cadmium tolerance of *Zea mays* roots was investigated, which previously treated with the endophyte *Exophiala pisciphila*. Roots of three-day-old maize seedlings were first inoculated with

the fungus (with=DSE, without=nDSE). 10 days later, plants were fertilized with cadmium for 31 days (with=Cd, without=nCd). Plants not treated with cadmium and/or the fungus served as the control. Finally, roots were harvested and mRNA was extracted and analyzed.

We found one group of Kwl3 to be strongly expressed. In the case where the fungi were absent, this group show a strong down-regulation with cadmium (4 L2FC) but no change upon fungal treatment was detectable. If cadmium is absent, the infection does not significantly impact expression but if cadmium is introduced into the system we see an up-regulation (3.5 L2FC) in infected plants.

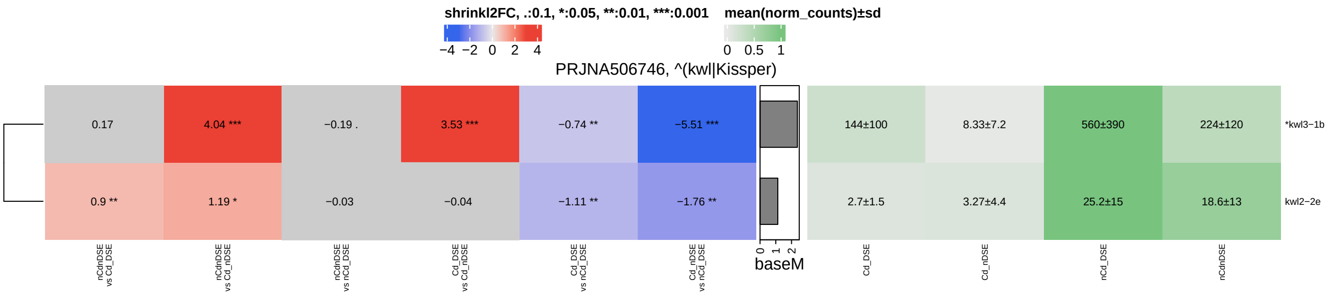

5.1.3 Pathogenic but no symbiotic response: P+S

Cucumis sativus (PRJNA445328)

To better understand *Trichoderma*-induced plant resistance to many plant pathogens, cucumber plants were infected with *Botrytis cinerea* in the presence or absence of *Trichoderma* in Yuan et al. (2019). At the three-leaf stage, plants were inoculated with *Trichoderma* and 24 hours later *B. cinerea* was injected into the leaves. Samples of the leaves were harvested 96 hours later and examined for differential gene expression.

We detected 3 groups of kwl1 Kissper-Kiwellins. One group was highly expressed and showed a significant up-regulation in response to the symbiont and pathogen (≈ 2.5 L2FC).

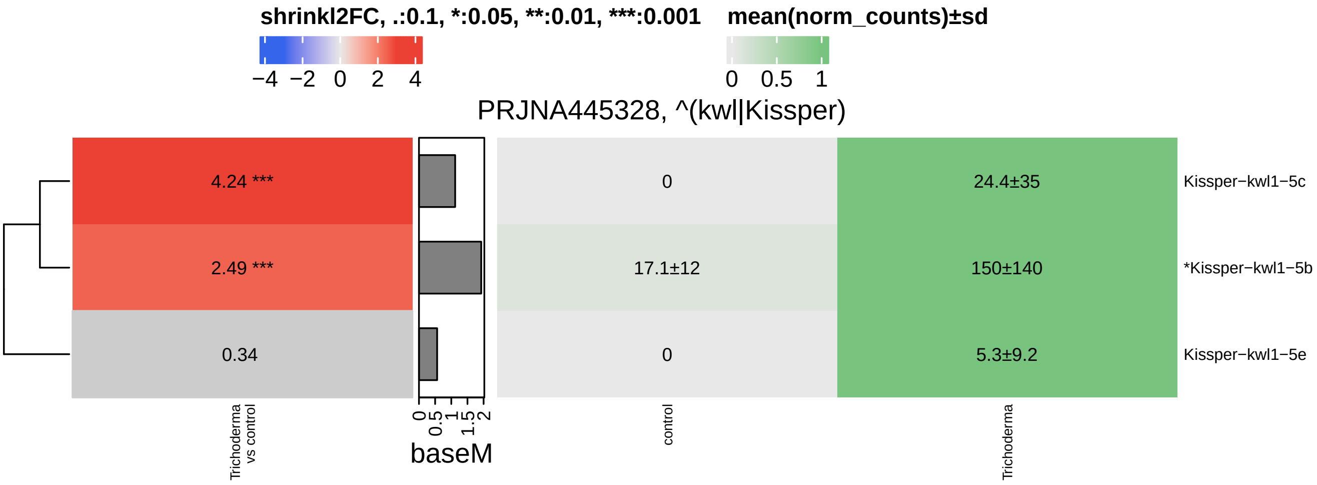

5.1.4 Pathogenic, symbiotic and tissue specific effect: P+S+T

Triticum aestivum (PRJEB21874)

Triticum aestivum was infected with the bacterial pathogenic *Xanthomonas translucens* in Fiorilli et al. (2018). It was tested whether the mycorhizal fungus *Funneliformis mossae* influenced the infection. After plants were colonized by mycorrhiza for 49 days, plants were inoculated with the pathogenic bacterium. One day after infection, samples of roots and leaves were isolated and the mRNA levels of the three species were examined.

We found 8 highly expressed Kiwellin groups most of which belong to Kwl2 but and to Kwl3. The Kwl3 group showed no significant response and the results for Kwl2 were mixed. In roots, we observed one group of Kwl2 to be down-regulated (≈ 2 L2FC) and one group to be up-regulated (1 L2FC) in response to the pathogen and symbiont. Differences between roots and leaves can be observed for Kwl1, Kwl2, and Kwl3. Overall the expression strength in roots was observed to be higher compared to leaves.

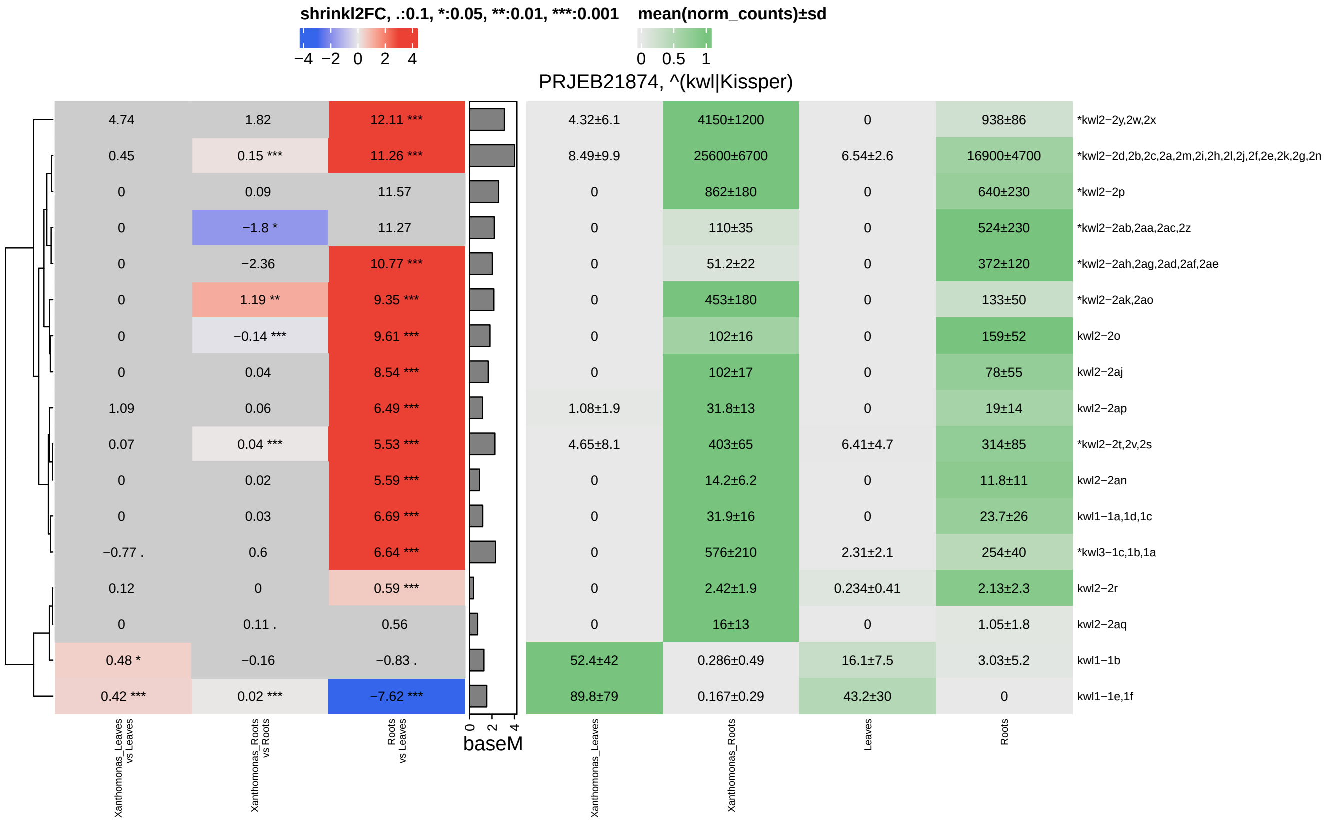

5.1.5 Symbiotic and abiotic response and tissue-specific effects: S+A+T

Medicago truncatula (PRJNA524006)

In Sańko-Sawczenko et al. (2019), the Fabaceae *Medicago truncatula* was evaluated for their response to water stress when the roots were inoculated by nitrogen-fixing bacteria *Sinorhizobium meliloti*. After the roots were successfully colonized by the bacteria, the plants were subjected to water stress. For this, the plants were not watered for up to 4 days after colonization. At the end of the four days, root nodules were harvested from watered and non-watered plants. Uninfected plants served as control, here the roots were harvested. The mRNA was isolated from the collected samples and analyzed.

We found one highly expressed group of Kwl3 that shows a strong up-regulation after 4 days of water withdrawal (2 L2FC).

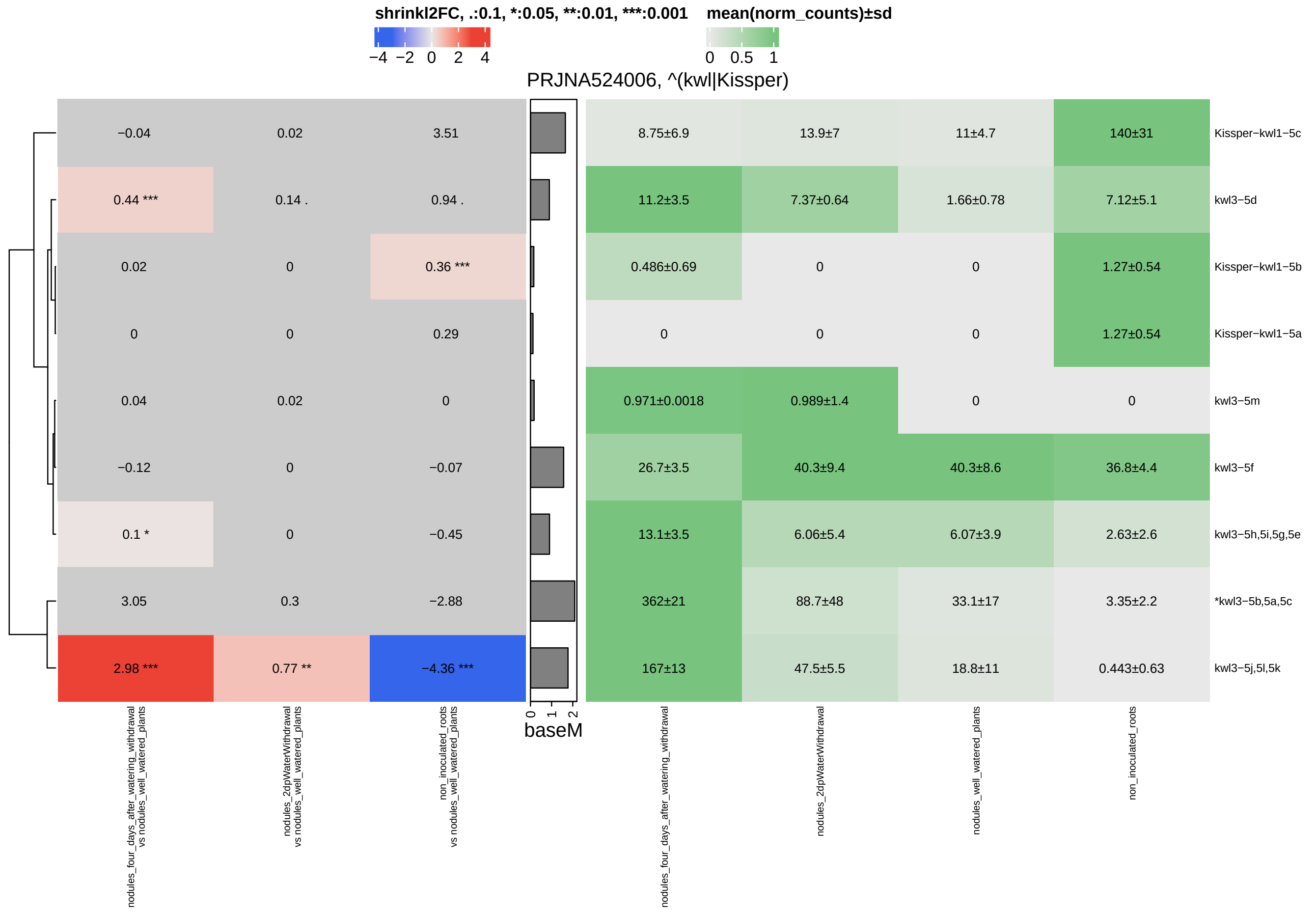

5.1.6 Symbiotic but no pathogenic response: S(+P)

Solanum lycopersicum (PRJNA795851)

Biological control agents (BCA) play a major role to combat plant pathogens. Singh et al. (2021) aimed to investigate the transcriptonal response to treat with the BCA fungus *Chaetomium globosum* (Cg) on plants infected with the pathogenic fungi *Alternaria solani* (As). First, 21-day-old tomato plants were inoculated with the BCA. Another 24 hours later, the plants were spray-inoculated with the pathogen. After five days of infection, infected leaves were harvested, and RNA was isolated and sequenced. In total, four data sets resulted from this experiment: plants not infected (CONTROL), plants infected with both fungi (Cg_As_inoculated), and plants infected with only one fungus each (Cg_inoculated, As_inoculated).

We found one group of Kwl3 to be highly expressed. An up-regulation could be observed in case of infection with the BCA fungus *C. globosum* ($\approx$ 4 L2FC). Furthermore, a slight down-regulation (below 1 L2FC) was observed upon infection with the pathogen *A. solani*. In response to combinatorical treatment with the pathogen, a slight up-regulation (below 1 L2FC) was observed.

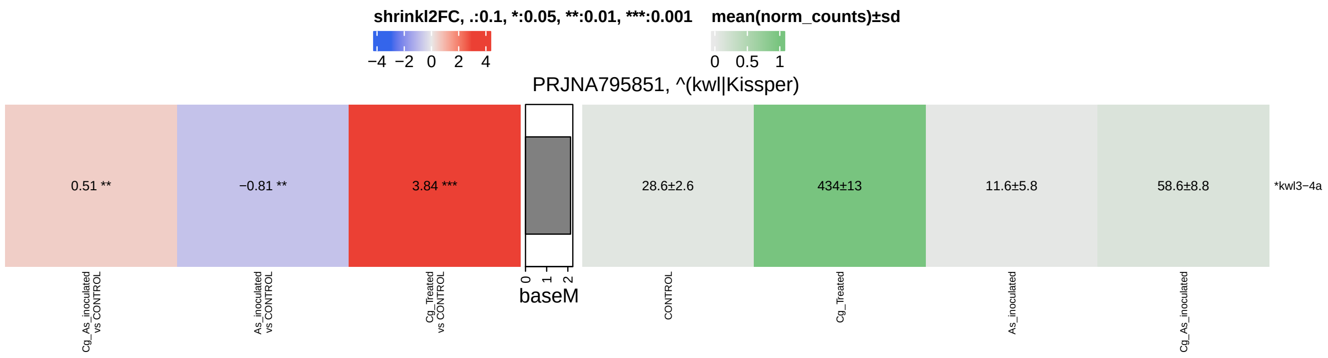

5.1.7 Tissue specific but no symbiotic response: T(+S)

Medicago truncatula (PRJNA79233)

In Boscari et al. (2013), the transcription of developing nodules on *Medicago truncatula* was investigated. For this purpose, plants were infected with their symbiont *Sinorhizobium meliloti* and samples were taken from different stages of the nodules/roots and the mRNA was isolated and sequenced. Roots of the plant that were not infected were collected 4 days after infection (developing nodules), and 12 days after infection (matured nodules) were examined. Furthermore, a nitric oxide scavenger (cPTIO) was added to an infected plant and the effect on nodules was studied. Thus, a total of four data sets were obtained and compared: unified roots (MtRoots), infected roots (MtInoc), nodules (MtNod), and infected roots treated with cPTIO (MtInocCPTIO). All samples except the nodule sample were collected and analyzed 4 days after infection or mock infection.

We found 2 groups of Kwl3 to be strongly expressed but no difference between infected and non-infected roots (cPTIO independent) was observed. Remarkably, Kiwellins were almost exclusively found in nodules.

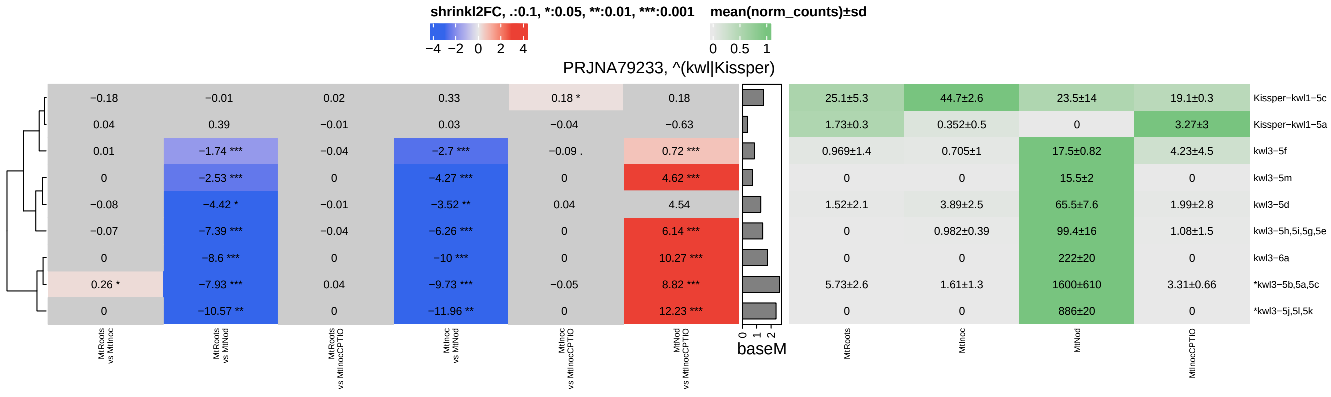

5.1.8 Tissue specific but no pathogenic response: T(+P)

Musa acuminata (PRJNA417328)

Benzothiadiazole (BTH) is an inducer of plant resitance that stimulates the defense response in bananas and protects against pathogen infection. In Cheng et al. (2018), via RNA-seq, the effect of BTH was investigated at the gene expression level by spraying young plants with a BTH solution. For this purpose, plant samples from roots (RT) and leaves (LF) 1 and 3 days post-infection with the fungal pathogen *Fusarium oxysporum* were compared with their respective controls (0 dpi). We found one group of highly

expressed Kwl2 members (in roots but almost absent in leaves). Furthermore, no significant changes were observed in response to BTH. Kiwellins were almost exclusively found in roots (compared to leaves).

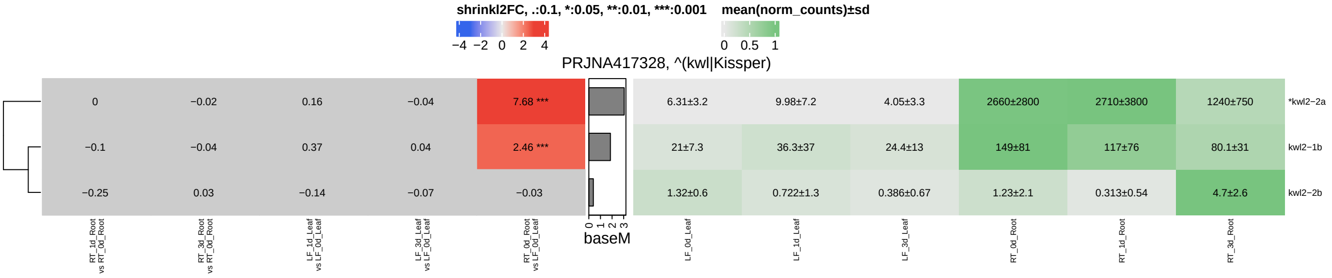

5.2 Significant response and weak expression

5.2.1 Pathogenic response: P

Actinidia chinensis (PRJNA436459)

Michelotti et al. (2018) investigated the effect of acibenzolar-A-methyl (ASM, a bactericidal component) on the course of infection of *Pseudomonas syringae pv. actinidiae* on its host the kiwifruit plant (*Actinidia chinensis*). For this purpose, plants were treated with or without ASM and inoculated with the bacterium or buffer. 3, 24, and 48 hpi samples were obtained, and the mRNA was isolated from ground tissue and analyzed.

We found 4 groups of Kiwellins of which 2 are Kissper-Kiwellins (Kwl1) and two belong to Kwl3. The groups of kwl1 are moderately expressed in one group and we see an ASM-specific up-regulation of $\approx 1 - 2$ L2FC. In all comparisons without ASM, no effects are were observed throughout the infection.

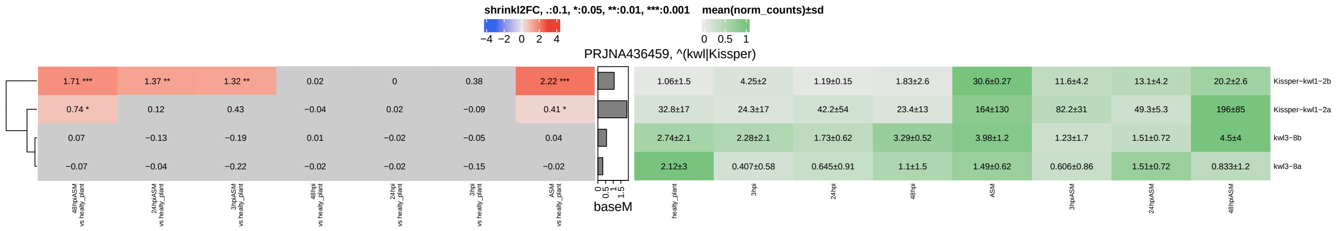

Physcomitrium patens (PRJNA751102)

Otero-Blanca et al. (2021) investigated the defense mechanisms of *Physcomitrium patens* against *Colletotrichum gloeosporioides*. For this purpose, plants were spray-inoculated with the pathogen. Samples were harvested and analyzed 8 and 24 hours after infection and uninfected plants served as controls.

We found a group of two Kissper-Kiwellins from Kwl1 (here called kissper-kwl0nsp). Although this group was not strongly expressed we found a response to the infection at both time points ($2 - 3$ L2FC).

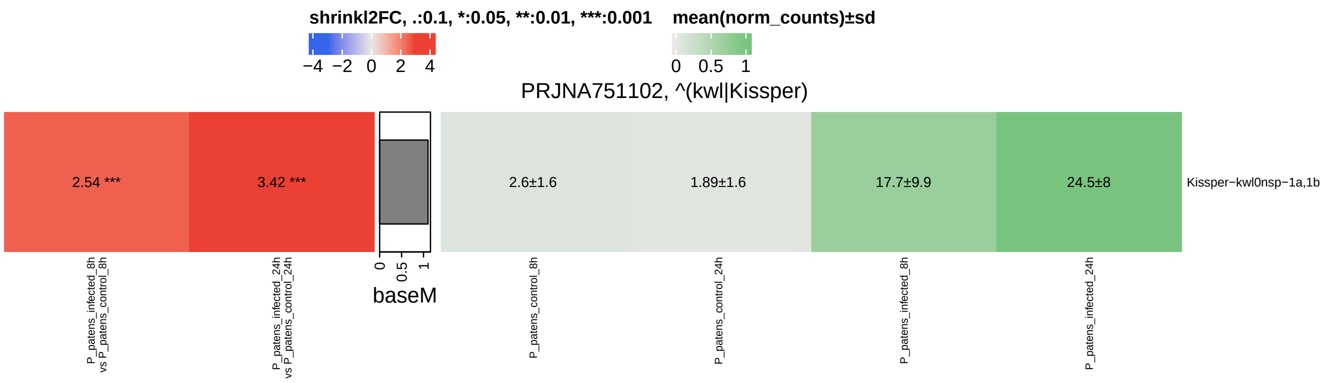

Zea mays (PRJNA529541)

Garcia-Ceron et al. (2021) infected *Zea mays* with *Fusarium graminearum* and examined the change in gene expression. For this purpose, the leaves of the plants were injured and disk-infected with the fungus. Leaf tissue was collected after 3, 5, and, 7 days respectively, and the mRNA was examined. The Comparison was made with uninfected plants and fungi grown in axenic culture. No Kiwellin was found to be highly expressed but a down-regulation for a group of Kwl2 and a group of Kwl3 was observed upon infection ($\approx 2-4$ L2FC).

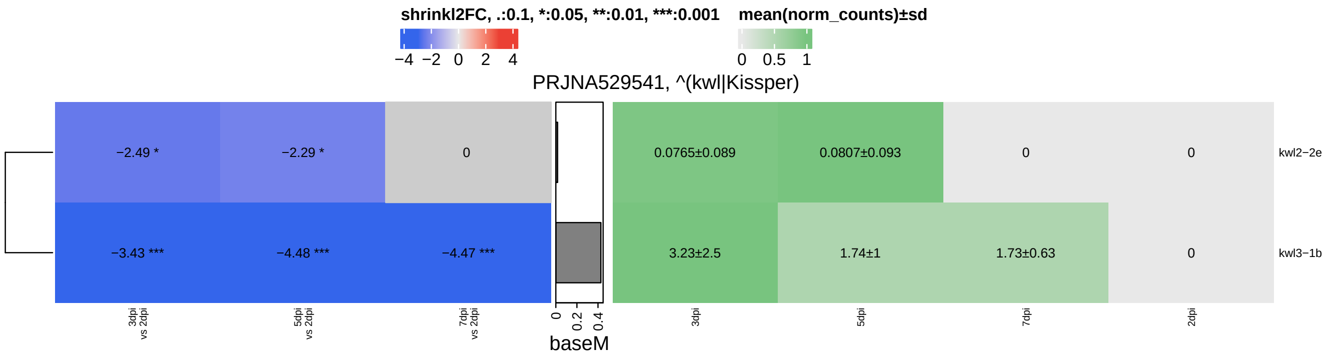

Zea mays (PRJNA415355)

In Li et al. (2018b), mRNA levels were examined organ-specifically during tumor development of *Ustilago maydis* on *Zea mays*. For this purpose, data sets of bundle sheaths and the mesophyll of fungus-infected plants were compared with water-inoculated plants (mock). We found no strongly expressed Kiwellin groups but an up-regulation ($\approx 2-4$ L2FC) of Kwl3 in infected bundle sheath and mesophyll tissue.

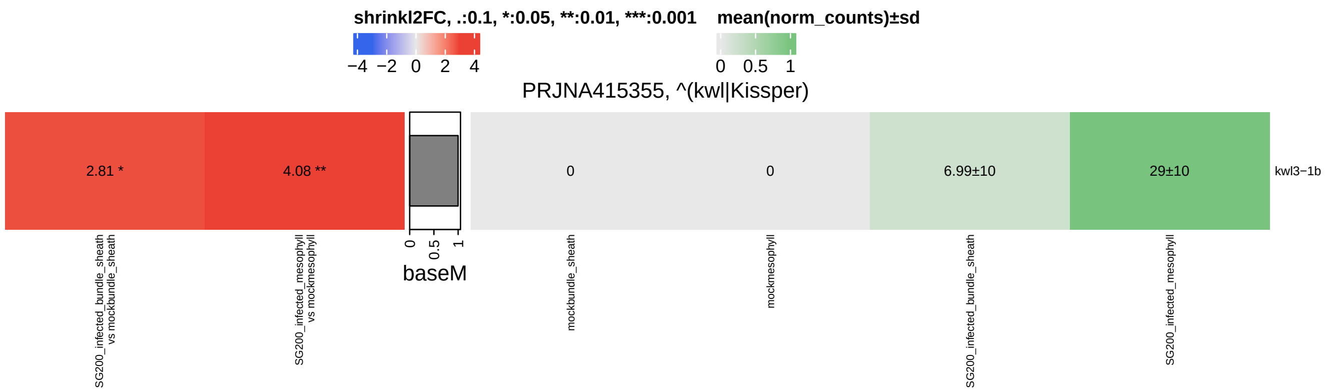

5.2.2 Pathogenic and tissue specific response: S+T

Glycine max (PRJNA531615)

Adhikari et al. (2019) investigated the influence of nodulation on the roots of the soybean plant. For this purpose, *Glycine max* was infected with the bacterium *Bradyrhizobium diazoefficiens*. The infected root tissue on which nodules were formed was harvested at $5-7$ days after infection (emerging nodules) or $14-16$ days after infection (mature nodules). Root tissue was collected above and/or below the nodules as control groups.

We found no strongly expressed Kiwellin group but differences were found e.g. between emerging and mature nodules as well as between mature nodules and uninfected roots (NA_root).

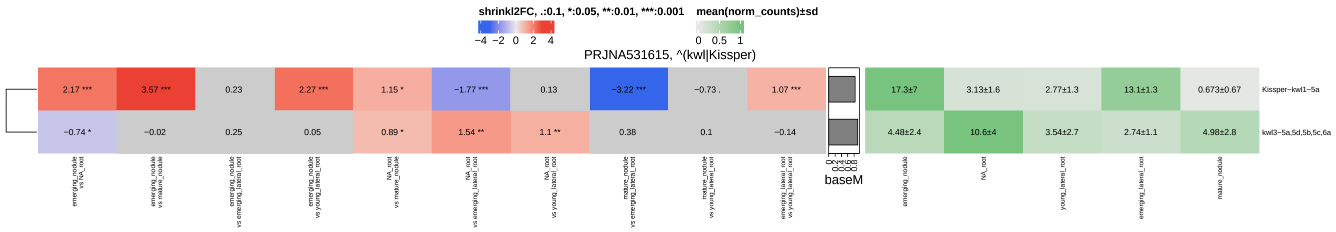

5.3 Strong expression but no significant response

5.3.1 Pathogenic response: P

Cucumis melo (PRJEB15551)

Two strains of *Cucumis melo* were infected with *Fusarium oxysporum f. sp. melonis* Snyd. & Hans race 1.2 (FOM1.2) in Silvia Sebastiani et al. (2017). One of the melon lines is the NAD strain, which is capable of early recognition of pathogens and developing resistances. The second melon genotype Charentais (CHT) is susceptible to the fungus. Plantlets of the two strains were infected with the fungus and 1 and 2 days. Stems of the small plants were harvested and mRNA levels were determined and compared.

In our reanalysis, we found one highly expressed group of Kwl1 (Kissper-Kiwellin). None of the groups are significantly differentially expressed.

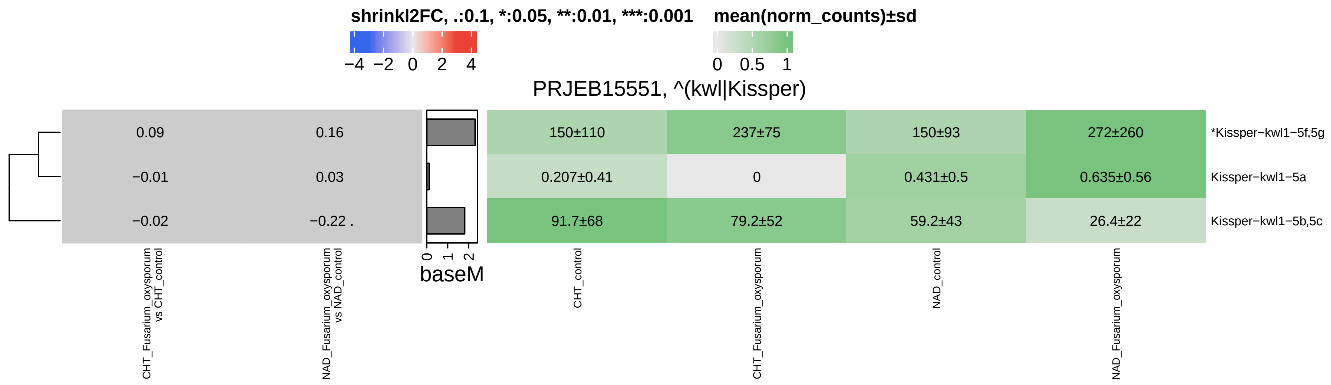

Solanum tuberosum (PRJNA755645)

Alternaria solani is a necrotrophic fungus that infects potatoes and other crops. The aim of Brouwer et al. (2021) was to investigate how the transcriptome of *Solanum tuberosum* changes during infection with this fungus. For this purpose, the leaves of six-week-old potato plants were infected with the pathogen. Samples of infected leaves were collected 1, 6, 12, 24, and 48 hours after infection, and the mRNA was analyzed and sequenced. Uninfected plants served as the control group (0 hpi).

Our reanalysis was able to identify Kiwellins 3 groups of Kissper-Kwl1. One group can be considered as strongly expressed but no response to the infection was observed.

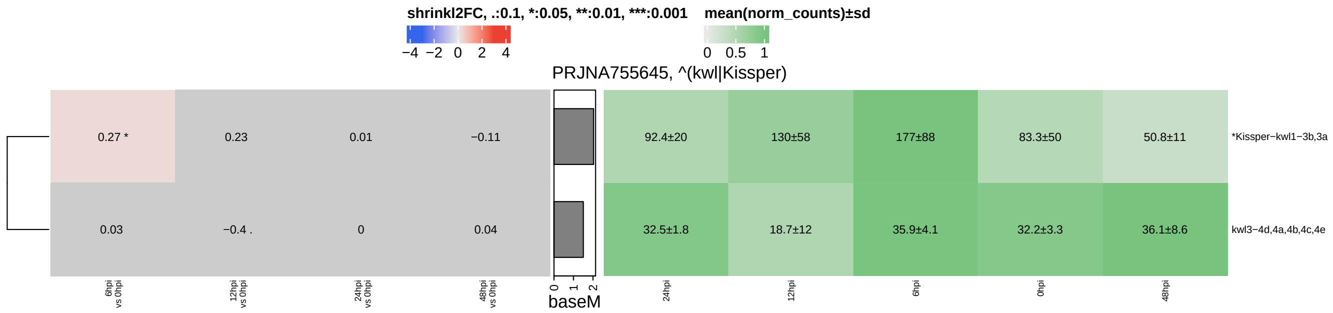

5.3.2 Pathogenic and symbiotic response: P+S

Oryza rufipogon and *Oryza sativa* (PRJNA476551)

Tian et al. (2019) investigated the differences between wild rice (*Oryza rufipogon*) and cultivated rice (*Oryza sativa*) inoculated with the arbuscular mycorrhizal fungus *Rhizoglomus intraradices* upon infection with the pathogen *Magnaporthe oryzae*. For this purpose, ten-day-old rice plants were first inoculated with the mycorrhizal fungus, and after another 45 days, the leaves of the plants were spray-inoculated with the pathogen. After another seven days, the roots of the plants were harvested and the RNA was isolated and analyzed.

For both *Oryza* species we found a group of Kwl3 to be highly expressed but no significant differential changes were observed.

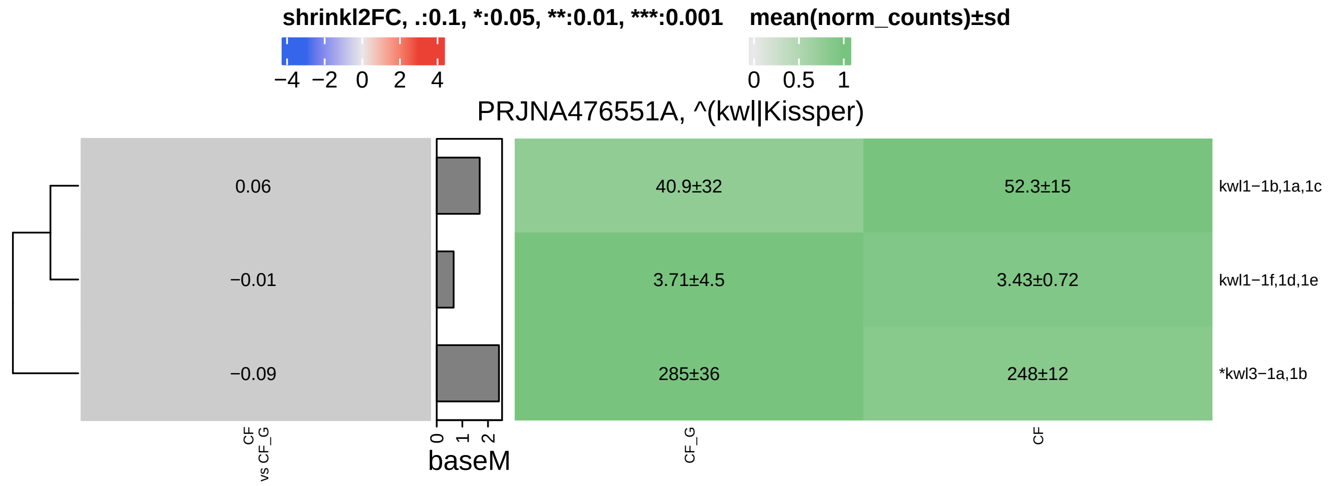

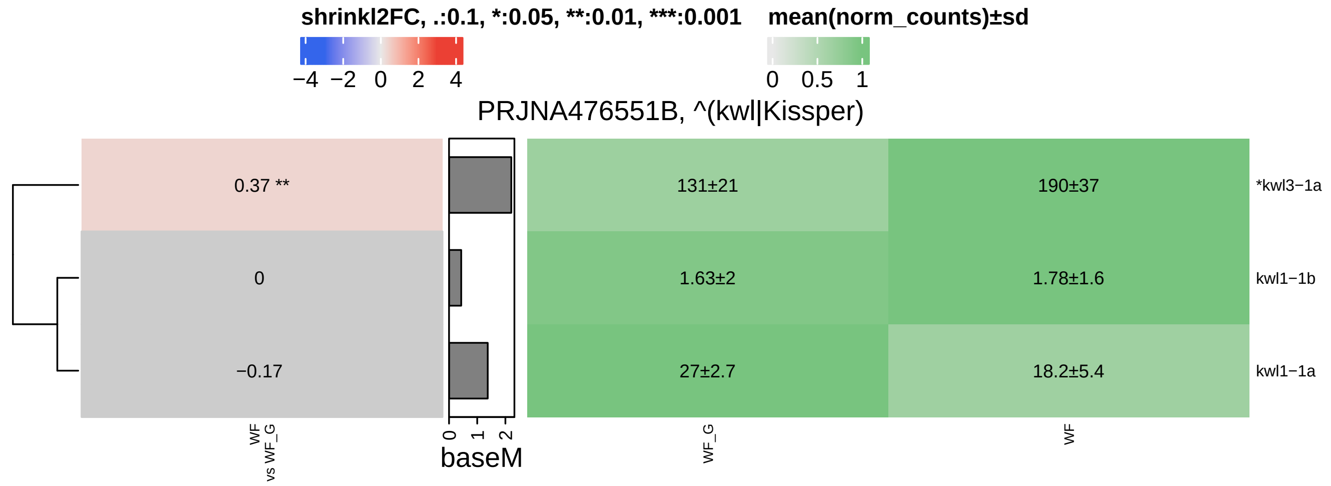

5.4 Weak expression and no significant response

5.4.1 Symbiotic response: S

***Glycine max* (PRJDB9752)**

Roots of *Glycine max* strain EN1282 (nfr1-mutant - a strain lacking in a Nod factor receptor) was infected with the symbiotic bacterium *Bradyrhizobium elkanii* USDA61 in Ratu et al. (2021). The wild-type strain of the bacterium was compared with a T3SS (Type 3 Secretion system) deletion strain. The roots of the seedlings were harvested 30 days after infection and mRNA levels of the bacterium and the plant were measured.

We found 3 groups of Kiwellins of which 2 belong to Kwl3 and one Kissper-Kiwellins to Kwl1. The Kwl3 groups were weakly expressed and Kissper-Kiwellin showed a moderate expression. Furthermore, all groups in this experiment showed no significant response to the infection.

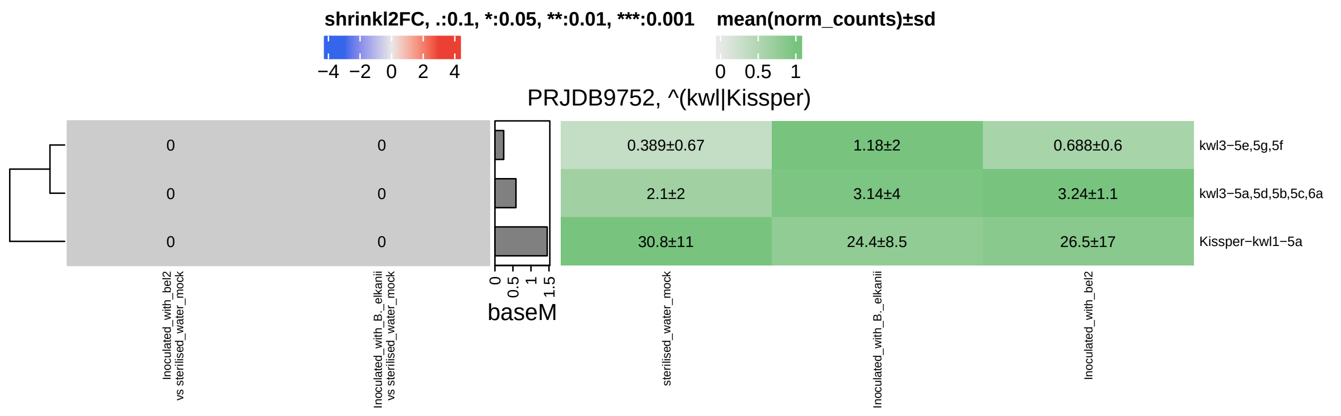

Glycine max (PRJNA396797)

To investigate transcriptional changes associated with nodule formation genes in soybean, *Glycine max* roots were infected with *Bradyrhizobium japonicum* in Hayashi et al. (2012). Two strains of the bacterium were used and compared: a wild-type strain and a NodC strain that cannot synthesize Nod factors. The infected roots were harvested at 2 dpi, the mRNA was analyzed and compared.

We found a Kwl1 (Kissper-Kiwellins) and one Kwl3 group. No group was strongly expressed or differentially regulated in this experimental setup.

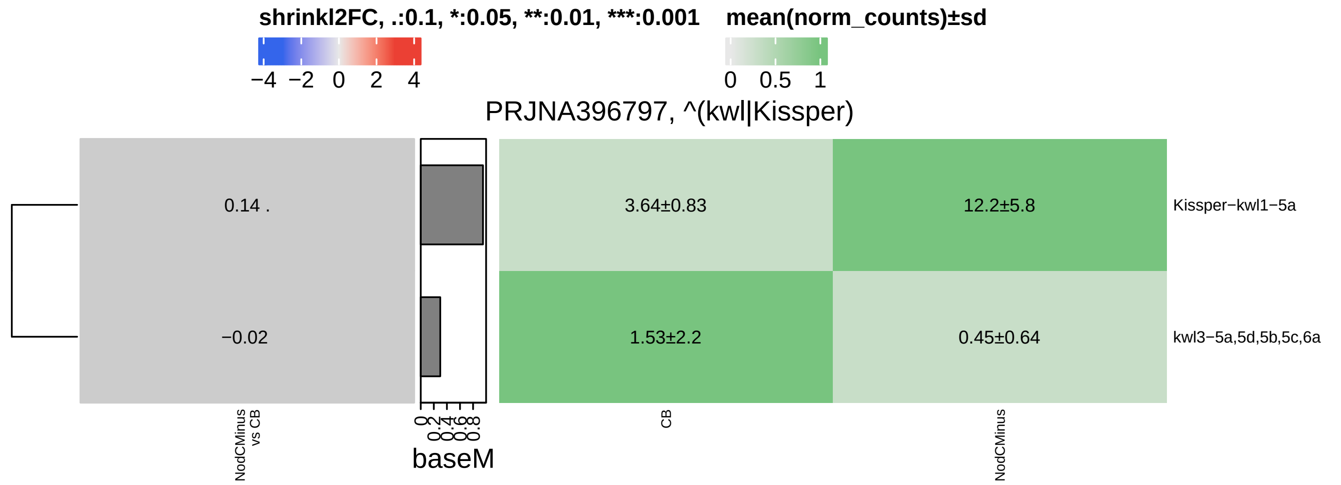

Glycine max (PRJNA579169)

Using the *Rj2* allele, soybean plants can exclude poorly nitrogen-fixing or less useful rhizobia such as *B. japonicum* USDA122 or *Rhizobium fredii* USDA257 from a symbiotic relationship. Host immunity is mediated by the secretory rhizobium type-III-protein NopP and the previously described host resistance protein Rj2. In Shine et al. (2019) transcriptional changes in leaves of *Rj2* virus-silenced plants, each infected with buffer, or one of the two rhizobacteria, will be used to better understand the mechanism of systemic resistance induced by incompatible rhizobia. For this purpose, infected roots were harvested and the mRNA was isolated and analyzed.

We found 2 groups of weakly expressed Kiwellins (Kissper-Kiwellins of Kwl1 and Kwl3). But in all comparisons, no differential regulation was detected.

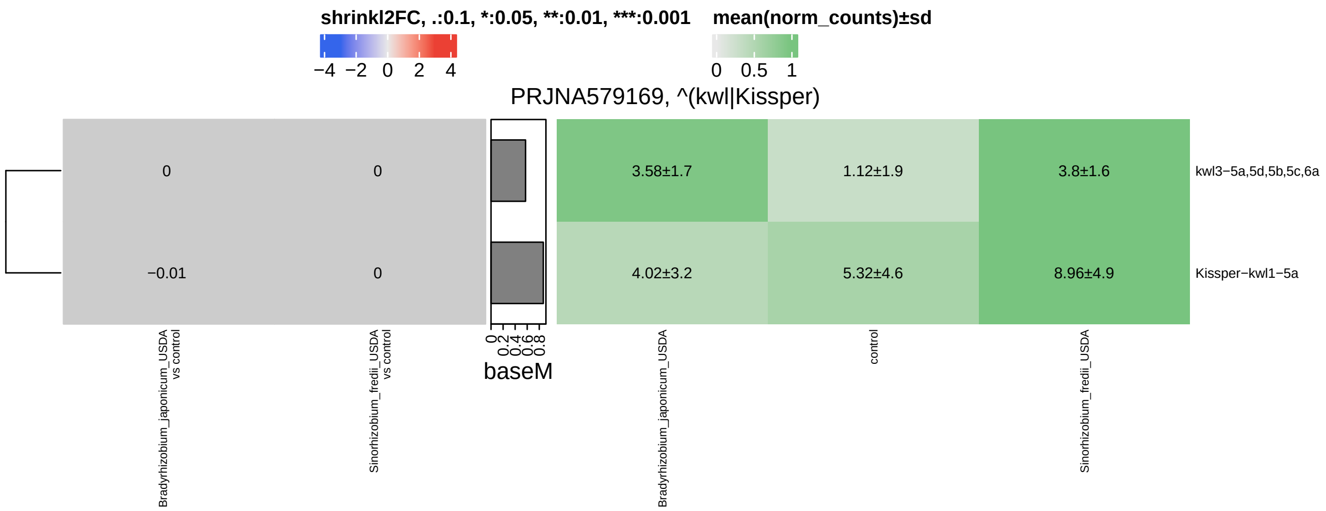

Solanum lycopersicum (PRJNA531604)

In Li et al. (2018a) the influence of the endophyte *Pochonia chlamydosporia* on the response of *Solanum lycopersicum* was investigated. For this purpose, plants were infected with the fungus, and samples of the roots were harvested 4, 7, and 21 days after infection. From these tissue samples, mRNA was isolated and analyzed.

No Kiwellin was found to be highly expressed and no differential regulation was observed in response to the fungi.

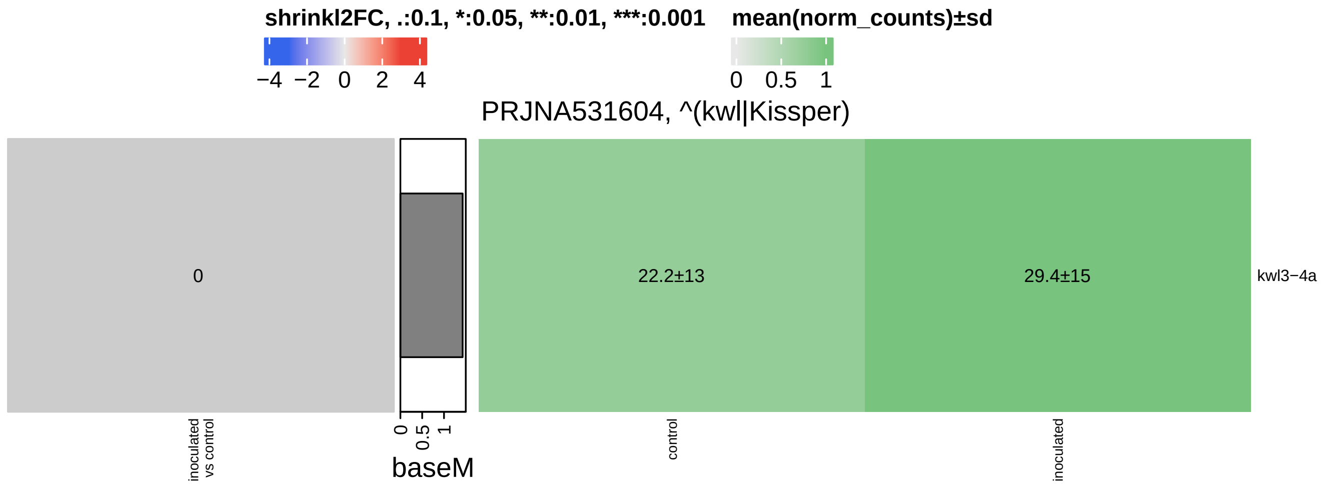

5.4.2 Pathogenic response: P

Musa acuminata (PRJNA287860)

Roots of two-month-old banana seedlings were infected with *Fusarium oxysporum* Race 4 (FocR4)-C1 HIR in Munusamy and Zaidi (2021). Infected root samples were harvested at 2, 48, and 96 hours. The 2 hpi root sample represents the control with which the other two samples were compared.

Our reanalysis found 3 weakly to moderately expressed Kiwellins belonging to Kwl2. Neither of these groups shows differential regulation in this experiment.

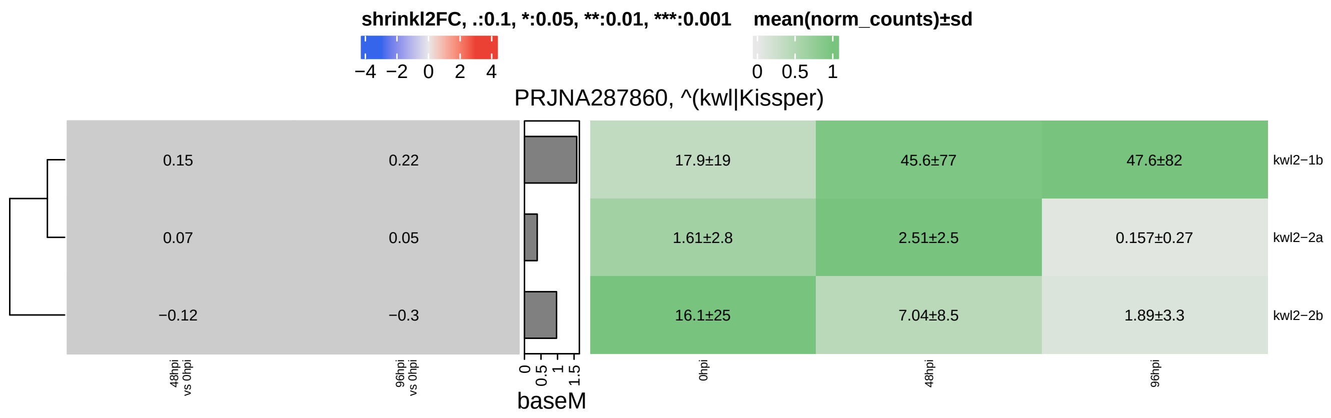

Zea mays (PRJNA551023)

Pantoea stewartii is the causal agent of Stewart's bacterial wilt of corn and is investigated in Doblas-Ibáñez et al. (2019). With the help of a mutation in the *pan1* gene, it is possible to create resistant corn plants to bacterial disease. Consequently, heterozygous and homozygous (related to the *pan1* gene) maize lines were created by crosses and infected with the bacterium. Subsequently, infected material was harvested one day post-infection, mRNA was isolated, and differences in transcription levels between the different maize lines infected or mock-infected were analyzed.

We found Kwl3-1a and Kwl2-2e but both were neither strongly expressed nor showed any differential response.

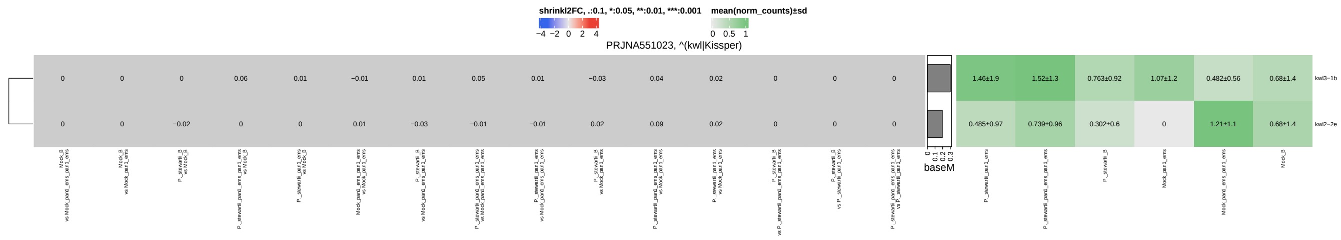

Solanum lycopersicum (PRJNA487149)

In Fawke et al. (2019), the influence of glycerol-3-phosphate acyltransferases on the resistance of *Solanum lycopersicum* to its host *Phytophthora infestans* was investigated. For this purpose, tomato wild-type plants and plants with a loss-of-function mutation in the *gpat6* gene were infected with the fungus. Three days after infection, the leaves were harvested, the RNA isolated, reverse transcribed and the data analyzed.

We found one member of the Kwl3 group that was neither strongly expressed nor showed a differential response.

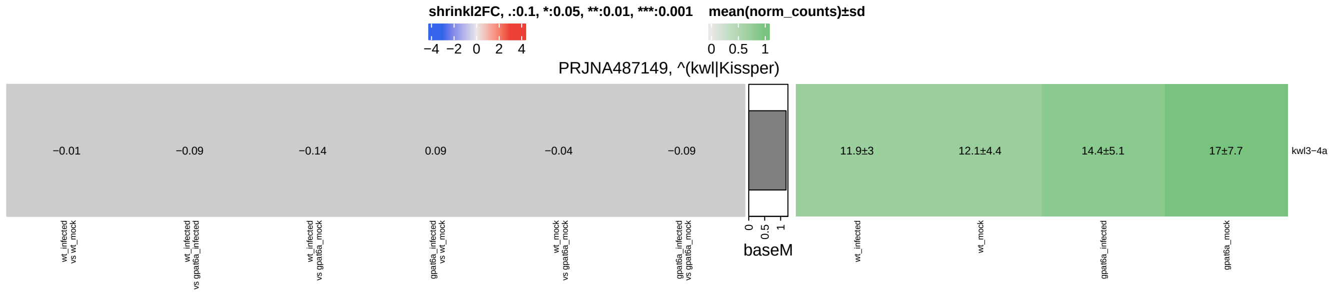

5.4.3 Pathogenic and symbiotic response: P+S

Chenopodium quinoa (PRJNA720675)

In Rollano-Peñaloza et al. (2021), the authors aimed to investigate the influence of *Trichoderma* on *Chenopodium quinoa*. For this purpose, two strains each of the fungi *Trichoderma afroharzianum* (T22) and *Trichoderma harzianum* (BOL-12) and the plant (*Chenopodium quinoa* Kurmi and *Chenopodium quinoa* Real) were co-cultured with each other. Subsequently, RNA was extracted from the roots and sequenced to determine the differentially regulated genes in the 4 strains.

We found 2 Kiwellin groups of Kwl3 but no group was found to be highly expressed and no differential regulation was observed.

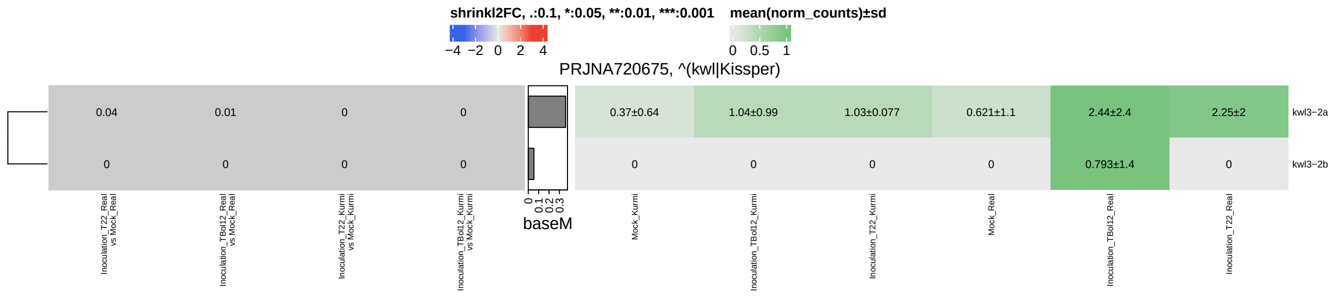

Triticum aestivum (PRJEB8798)

In Rudd et al. (2015) wheat was infected with its fungal pathogen *Zymoseptoria tritici* and mRNAs were isolated from leaves 1, 4, 9 and 14 dpi (infected=Z.tritici, mock innoculated=M). This data set was compared with mRNAs from buffer-infected plants and fungus growing in liquid culture.

Generally, we observe high variations among all expression values and neither strong expression nor differential response was observed.

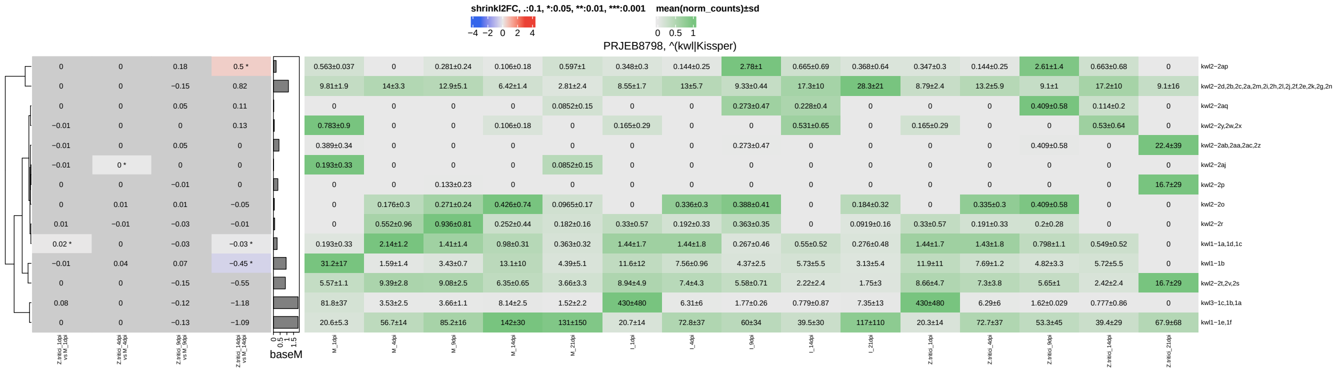

REFERENCES

Adhikari, S., Damodaran, S., and Subramanian, S. (2019). Lateral root and nodule transcriptomes of soybean. *Data* 4, 64

Almagro Armenteros, J. J., Tsirigos, K. D., Sønderby, C. K., Petersen, T. N., Winther, O., Brunak, S., et al. (2019). Signalp 5.0 improves signal peptide predictions using deep neural networks. *Nature biotechnology* 37, 420–423

[Dataset] Andrews, S. et al. (2010). Fastqc: a quality control tool for high throughput sequence data

Boscari, A., Del Giudice, J., Ferrarini, A., Venturini, L., Zaffini, A.-L., Delledonne, M., et al. (2013). Expression dynamics of the medicago truncatula transcriptome during the symbiotic interaction with sinorhizobium meliloti: which role for nitric oxide? *Plant physiology* 161, 425–439

Brouwer, S. M., Brus-Szkalej, M., Saripella, G. V., Liang, D., Liljeroth, E., and Grenville-Briggs, L. J. (2021). Transcriptome analysis of potato infected with the necrotrophic pathogen alternaria solani. *Plants* 10, 2212

Burkhardt, A. and Day, B. (2016). Transcriptome and small rnaome dynamics during a resistant and susceptible interaction between cucumber and downy mildew. *The Plant Genome* 9, plantgenome2015–08

Cheng, Z., Yu, X., Li, S., and Wu, Q. (2018). Genome-wide transcriptome analysis and identification of benzothiadiazole-induced genes and pathways potentially associated with defense response in banana. *Bmc Genomics* 19, 1–19

Consortium, U. (2019). Uniprot: a worldwide hub of protein knowledge. *Nucleic acids research* 47, D506–D515

DeLano, W. L. and Bromberg, S. (2004). Pymol user's guide. *DeLano Scientific LLC* 629

Doblas-Ibáñez, P., Deng, K., Vasquez, M. F., Giese, L., Cobine, P. A., Kolkman, J. M., et al. (2019). Dominant, heritable resistance to stewart's wilt in maize is associated with an enhanced vascular defense response to infection with pantoea stewartii. *Molecular Plant-Microbe Interactions* 32, 1581–1597

Eddy, S. R. (1998). Profile hidden markov models. *Bioinformatics (Oxford, England)* 14, 755–763

Fawke, S., Torode, T. A., Gogleva, A., Fich, E. A., Sørensen, I., Yunusov, T., et al. (2019). Glycerol-3-phosphate acyltransferase 6 controls filamentous pathogen interactions and cell wall properties of the tomato and nicotiana benthamiana leaf epidermis. *New Phytologist* 223, 1547–1559

Fiorilli, V., Vannini, C., Ortolani, F., Garcia-Seco, D., Chiapello, M., Novero, M., et al. (2018). Omics approaches revealed how arbuscular mycorrhizal symbiosis enhances yield and resistance to leaf pathogen in wheat. *Scientific Reports* 8, 1–18

Gamez, R. M., Rodríguez, F., Vidal, N. M., Ramirez, S., Vera Alvarez, R., Landsman, D., et al. (2019). Banana (musa acuminata) transcriptome profiling in response to rhizobacteria: Bacillus amyloliquefaciens bs006 and pseudomonas fluorescens ps006. *BMC genomics* 20, 1–20

Garcia-Ceron, D., Lowe, R. G., McKenna, J. A., Brain, L. M., Dawson, C. S., Clark, B., et al. (2021). Extracellular vesicles from fusarium graminearum contain protein effectors expressed during infection of corn. *Journal of Fungi* 7, 977

Grant, B. J., Rodrigues, A. P., ElSawy, K. M., McCammon, J. A., and Caves, L. S. (2006). Bio3d: an r package for the comparative analysis of protein structures. *Bioinformatics* 22, 2695–2696

Han, X., Altegoer, F., Steinchen, W., Binnebesel, L., Schuhmacher, J., Glatter, T., et al. (2019). A kiwellin disarms the metabolic activity of a secreted fungal virulence factor. *Nature* 565, 650–653

Hayashi, S., Reid, D. E., Lorenc, M. T., Stiller, J., Edwards, D., Gresshoff, P. M., et al. (2012). Transient nod factor-dependent gene expression in the nodulation-competent zone of soybean (glycine max [l.] merr.) roots. *Plant biotechnology journal* 10, 995–1010

Huang, H., Nguyen Thi Thu, T., He, X., Gravot, A., Bernillon, S., Ballini, E., et al. (2017). Increase of fungal pathogenicity and role of plant glutamine in nitrogen-induced susceptibility (nis) to rice blast. *Frontiers in plant science* 8, 265

Jumper, J., Evans, R., Pritzel, A., Green, T., Figurnov, M., Tunyasuvunakool, K., et al. (2020). Alphafold 2. *In Fourteenth Critical Assessment of Techniques for Protein Structure Prediction (Abstract Book*

Lanver, D., Müller, A. N., Happel, P., Schweizer, G., Haas, F. B., Franitza, M., et al. (2018). The biotrophic development of ustilago maydis studied by rna-seq analysis. *The Plant Cell* 30, 300–323

Lechner, M., Findeiß, S., Steiner, L., Marz, M., Stadler, P. F., and Prohaska, S. J. (2011). Proteinortho: detection of (co-) orthologs in large-scale analysis. *BMC bioinformatics* 12, 1–9

Li, M., Wang, R., Tian, H., and Gao, Y. (2018a). Transcriptome responses in wheat roots to colonization by the arbuscular mycorrhizal fungus rhizophagus irregularis. *Mycorrhiza* 28, 747–759

Li, M., Wang, R., Tian, H., and Gao, Y. (2018b). Transcriptome responses in wheat roots to colonization by the arbuscular mycorrhizal fungus rhizophagus irregularis. *Mycorrhiza* 28, 747–759

Michelotti, V., Lamontanara, A., Buriani, G., Orrù, L., Cellini, A., Donati, I., et al. (2018). Comparative transcriptome analysis of the interaction between actinidia chinensis var. chinensis and pseudomonas syringae pv. actinidiae in absence and presence of acibenzolar-s-methyl. *BMC genomics* 19, 1–22

Munusamy, U. and Zaidi, K. (2021). Elucidation of musa acuminata cv. berangan root infection by foc (tropical race 4) by rna sequencing and analysis. *Asian Journal of Plant Science & Research*

Otero-Blanca, A., Pérez-Llano, Y., Reboledo-Blanco, G., Lira-Ruan, V., Padilla-Chacon, D., Folch-Mallol, J. L., et al. (2021). Physcomitrium patens infection by colletotrichum gloeosporioides: Understanding the fungal–bryophyte interaction by microscopy, phenomics and rna sequencing. *Journal of Fungi* 7, 677

Rasoolizadeh, A., Labbé, C., Sonah, H., Deshmukh, R. K., Belzile, F., Menzies, J. G., et al. (2018). Silicon protects soybean plants against phytophthora sojae by interfering with effector-receptor expression. *BMC plant biology* 18, 1–13

Ratu, S. T. N., Teulet, A., Miwa, H., Masuda, S., Nguyen, H. P., Yasuda, M., et al. (2021). Rhizobia use a pathogenic-like effector to hijack leguminous nodulation signalling. *Scientific reports* 11, 1–15

Rollano-Peñaloza, O. M., Mollinedo, P. A., Widell, S., and Rasmusson, A. G. (2021). Transcriptomic analysis of quinoa reveals a group of germin-like proteins induced by trichoderma. *bioRxiv*

Rudd, J. J., Kanyuka, K., Hassani-Pak, K., Derbyshire, M., Andongabo, A., Devonshire, J., et al. (2015). Transcriptome and metabolite profiling of the infection cycle of zymoseptoria tritici on wheat reveals a biphasic interaction with plant immunity involving differential pathogen chromosomal contributions and a variation on the hemibiotrophic lifestyle definition. *Plant physiology* 167, 1158–1185

Sańko-Sawczenko, I., Łotocka, B., Mielecki, J., Rekosz-Burlaga, H., and Czarnocka, W. (2019). Transcriptomic changes in medicago truncatula and lotus japonicus root nodules during drought stress. *International Journal of Molecular Sciences* 20, 1204

Shen, M., Schneider, H., Xu, R., Cao, G., Zhang, H., Li, T., et al. (2020). Dark septate endophyte enhances maize cadmium (cd) tolerance by the remodeled host cell walls and the altered cd subcellular distribution. *Environmental and Experimental Botany* 172, 104000

Shine, M. B., Gao, Q.-m., Chowda-Reddy, R. V., Singh, A. K., Kachroo, P., and Kachroo, A. (2019). Glycerol-3-phosphate mediates rhizobia-induced systemic signaling in soybean. *Nature communications* 10, 1–13

Silvia Sebastiani, M., Bagnaresi, P., Sestili, S., Biselli, C., Zechini, A., Orrù, L., et al. (2017). Transcriptome analysis of the melon-fusarium oxysporum f. sp. melonis race 1.2 pathosystem in susceptible and resistant plants. *Frontiers in Plant Science* 8, 362

Singh, J., Aggarwal, R., Bashyal, B. M., Darshan, K., Parmar, P., Saharan, M., et al. (2021). Transcriptome reprogramming of tomato orchestrate the hormone signaling network of systemic resistance induced by chaetomium globosum. *Frontiers in plant science* 12

Tian, L., Chang, C., Ma, L., Nasir, F., Zhang, J., Li, W., et al. (2019). Comparative study of the mycorrhizal root transcriptomes of wild and cultivated rice in response to the pathogen magnaporthe oryzae. *Rice* 12, 1–19

Yuan, M., Huang, Y., Ge, W., Jia, Z., Song, S., Zhang, L., et al. (2019). Involvement of jasmonic acid, ethylene and salicylic acid signaling pathways behind the systemic resistance induced by trichoderma longibrachiatum h9 in cucumber. *BMC genomics* 20, 1–13

Zhang, W., Li, H., Wang, L., Xie, S., Zhang, Y., Kang, R., et al. (2022). A novel effector, cssp1, from bipolaris sorokiniana, is essential for colonization in wheat and is also involved in triggering host immunity. *Molecular Plant Pathology* 23, 218–236
