# Supplementary material for: Evolutionary reconstruction, nomenclature and functional meta-analysis of the Kiwellin protein family
Source: Front Plant Sci. 2022 Dec 22;13:1034708. doi: 10.3389/fpls.2022.1034708 (PMC9813671; doi:10.3389/fpls.2022.1034708)

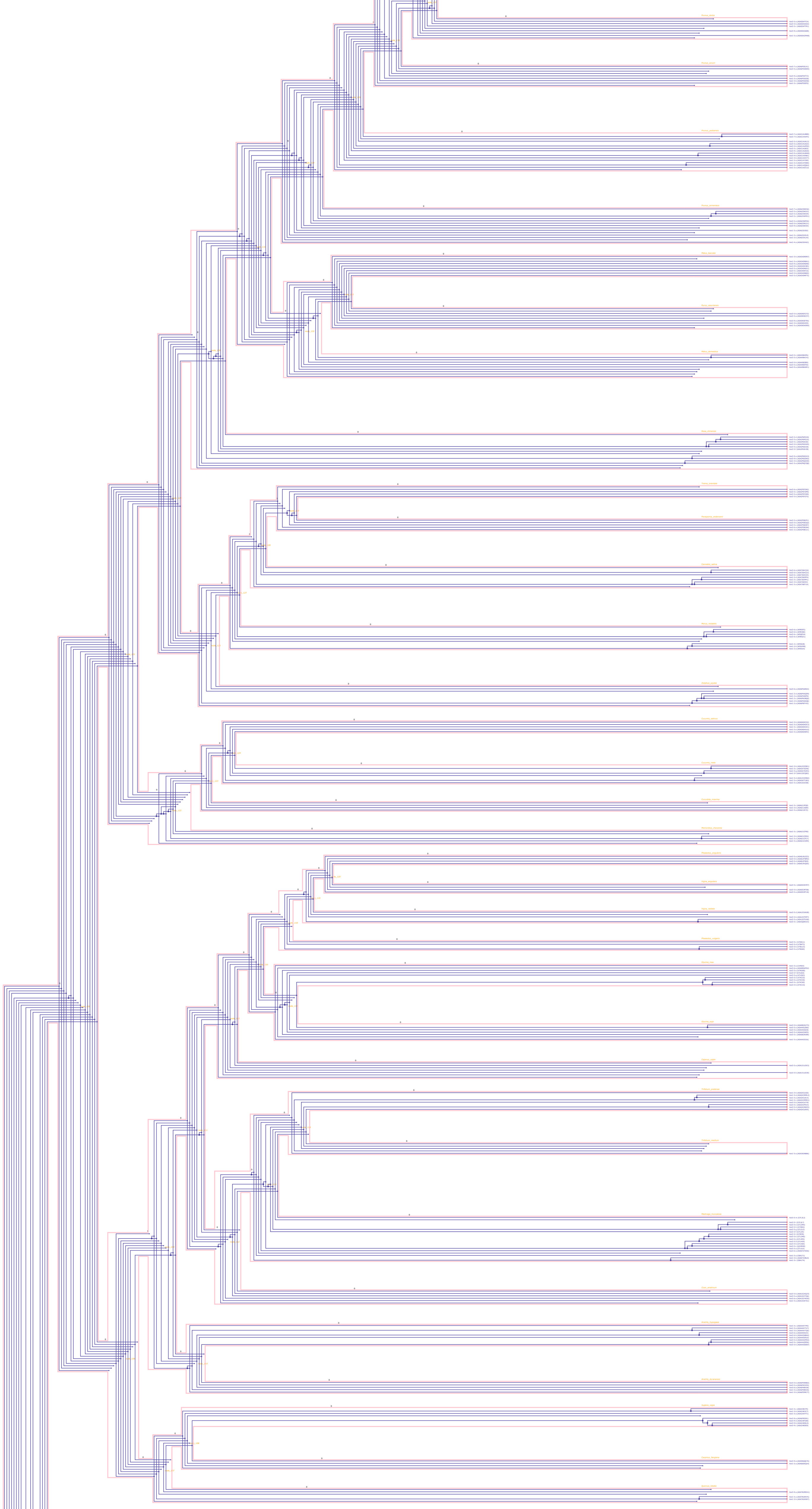
Prunus_dulcis
Kwl3-5-a (A0A5E4FTC4)
Kwl3-5-b (A0A5E4G5S4)
Kwl3-5-c (A0A5E4FTM1)
Kwl3-9-a (A0A5E4G608)
Kwl1-5-a (A0A5E4GPW8)
Prunus_avium
Kwl3-7-a (A0A6P5SLX1)
Kwl3-5-a (A0A6P5S8W0)
Kwl3-9-a (A0A6P5STY3)
Kwl1-5-a (A0A6P5SES8)
Kwl1-5-b (A0A6P5SD58)
Kwl1-5-c (A0A6P5SEP2)
Prunus_yedoensis
Kwl3-7-a (A0A314UBB9)
Kwl3-7-b (A0A314XXF5)
Kwl3-5-d (A0A314UA13)
Kwl3-5-a (A0A314UA22)
Kwl3-5-b (A0A314UFM2)
Kwl3-5-c (A0A314U8J5)
Kwl3-9-c (A0A314UAG0)
Kwl3-9-a (A0A314U8W0)
Kwl3-9-b (A0A314XN81)
Kwl1-5-b (A0A314ZH77)
Kwl1-5-a (A0A314YJN9)
Kwl1-5-d (A0A314YDB4)
Kwl1-5-c (A0A314ZQK4)
Kwl1-5-e (A0A314ZC12)
Prunus_armeniaca
Kwl3-7-a (A0A6J5WK56)
Kwl3-5-e (A0A6J5WJV3)
Kwl3-5-d (A0A6J5WIZ9)
Kwl3-5-c (A0A6J5WM51)
Kwl3-5-a (A0A6J5WPX6)
Kwl3-5-b (A0A6J5WL51)
Kwl3-9-a (A0A6J5WJ54)
Kwl1-5-a (A0A6J5X360)
Kwl1-5-c (A0A6J5XGL9)
Kwl1-5-b (A0A6J5XLX4)
Kwl1-4-a (A0A6J5XH63)
Malus_baccata
Kwl1-5-b (A0A540N9N7)
Kwl1-5-a (A0A540N8A1)
Kwl3-9-a (A0A540N6R8)
Kwl3-9-b (A0A540K385)
Kwl3-5-a (A0A540N6S1)
Kwl3-5-c (A0A540N7L6)
Kwl3-5-b (A0A540N865)
Kwl3-5-d (A0A540MFT5)
Pyrus_ussuriensis
Kwl3-5-b (A0A5N5F272)
Kwl3-5-a (A0A5N5EZ37)
Kwl3-9-a (A0A5N5EY90)
Kwl1-5-b (A0A5N5HII9)
Kwl1-5-a (A0A5N5HSR9)
Malus_domestica
Kwl3-5-c (A0A498KIP6)
Kwl3-5-d (A0A498KJY2)
Kwl3-5-b (A0A498JIB9)
Kwl3-5-a (A0A498JF05)
Kwl3-9-a (A0A498KNP1)
Rosa_chinensis
Kwl3-5-d (A0A2P6PG20)
Kwl3-5-e (A0A2P6PG10)
Kwl3-5-c (A0A2P6SCI0)
Kwl3-5-b (A0A2P6SH49)
Kwl3-5-a (A0A2P6SCI8)
Kwl3-5-f (A0A2P6SH38)
Kwl3-9-a (A0A2P6SGX3)
Kwl3-9-b (A0A2P6SDK0)
Kwl1-5-b (A0A2P6QH40)
Kwl1-5-a (A0A2P6Q7Q8)
Trema_orientale
Kwl3-6-a (A0A2P5F2N2)
Kwl3-5-c (A0A2P5F2M9)
Kwl3-5-a (A0A2P5F2N9)
Kwl3-5-b (A0A2P5F2T5)
Parasponia_andersonii
Kwl3-5-a (A0A2P5B2P1)
Kwl3-5-b (A0A2P5B2Q2)
Kwl3-5-c (A0A2P5B2N7)
Kwl3-6-a (A0A2P5B2N4)
Kwl1-2-a (A0A2P5BC21)
Cannabis_sativa
Kwl3-6-a (A0A7J6HC24)
Kwl3-6-b (A0A7J6HC23)
Kwl3-6-c (A0A7J6HC25)
Kwl1-5-d (A0A7J6DPF4)
Kwl1-5-c (A0A7J6HPP1)
Kwl1-5-b (A0A7J6EII2)
Kwl1-5-a (A0A7J6EY14)
Morus_notabilis
Kwl3-6-a (W9RSP2)
Kwl3-6-b (W9R384)
Kwl3-6-c (W9QFD4)
Kwl3-6-d (W9RSZ1)
Kwl1-2-c (W9SAJ8)
Kwl1-2-b (W9S2M8)
Kwl1-2-a (W9SAJ5)
Ziziphus_jujuba
Kwl3-6-a (A0A6P4ARK4)
Kwl1-2-d (A0A6P4AQ99)
Kwl1-2-e (A0A6P4AEP6)
Kwl1-2-c (A0A6P6GBQ0)
Kwl1-2-b (A0A6P4AEQ6)
Kwl1-2-a (A0A6P6FY43)
Cucumis_sativus
Kwl1-5-b (A0A0A0KS32)
Kwl1-5-d (A0A0A0KDC3)
Kwl1-5-c (A0A0A0KGH1)
Kwl1-5-e (A0A0A0KAU2)
Kwl1-5-a (A0A0A0KBF2)
Cucumis_melo
Kwl1-5-b (A0A1S3CNR1)
Kwl1-5-c (A0A5A7SZ96)
Kwl1-5-g (A0A5A7SVE5)
Kwl1-5-f (A0A1S3CQ82)
Kwl1-5-d (A0A1S3CNN2)
Kwl1-5-e (A0A5A7T1B3)
Kwl1-5-a (A0A1S3CIS8)
Cucurbita_maxima
Kwl1-5-c (A0A6J1JFQ0)
Kwl1-5-b (A0A6J1JAB9)
Kwl1-5-a (A0A6J1IE72)
Momordica_charantia
Kwl1-5-c (A0A6J1CFR6)
Kwl1-5-b (A0A6J1CER4)
Kwl1-5-d (A0A6J1CFC7)
Kwl1-5-a (A0A6J1CGR9)
Phaseolus_angularis
Kwl3-5-a (A0A0L9U3Z3)
Kwl3-5-b (A0A0L9TBM2)
Kwl3-5-d (A0A0L9TBJ0)
Kwl3-5-c (A0A0L9VQS5)
Vigna_angularis
Kwl3-5-c (A0A0S3SYM7)
Kwl3-5-b (A0A0S3RT68)
Kwl3-5-a (A0A0S3RT18)
Vigna_radiata
Kwl3-5-d (A0A1S3VAX8)
Kwl3-5-b (A0A1S3TSP7)
Kwl3-5-a (A0A1S3TUX8)
Kwl3-5-c (A0A3Q0EV23)
Phaseolus_vulgaris
Kwl3-5-c (V7AN11)
Kwl3-5-d (V7B4T3)
Kwl3-5-b (V7B1U3)
Kwl3-5-a (V7B260)
Glycine_max
Kwl1-5-a (I1M4I4)
Kwl3-6-a (A0A0R0KPS2)
Kwl3-5-e (K7MUB0)
Kwl3-5-f (K7LG64)
Kwl3-5-g (K7LG63)
Kwl3-5-d (K7KC32)
Kwl3-5-a (K7KC30)
Kwl3-5-c (K7KC40)
Kwl3-5-b (K7KC33)
Glycine_soja
Kwl3-5-b (A0A0B2Q1T5)
Kwl3-5-a (A0A445L6N0)
Kwl3-5-e (A0A445J6C8)
Kwl3-5-d (A0A445J6D5)
Kwl3-5-c (A0A0B2NV89)
Kwl1-5-a (A0A445ID26)
Cajanus_cajan
Kwl3-5-a (A0A151UCK3)
Kwl3-5-b (A0A151UCS9)
Trifolium_pratense
Kwl1-5-b (A0A2K3LIG0)
Kwl1-5-d (A0A2K3MML3)
Kwl1-5-a (A0A2K3L814)
Kwl1-5-c (A0A2K3MME1)
Kwl3-5-b (A0A2K3LF79)
Kwl3-5-c (A0A2K3P5V3)
Kwl3-5-d (A0A2K3MKP2)
Kwl3-5-a (A0A2K3LR95)
Trifolium_medium
Kwl1-5-a (A0A392NB86)
Medicago_truncatula
Kwl3-5-m (G7L3L2)
Kwl3-5-i (G7L3L7)
Kwl3-5-e (G7L3M4)
Kwl3-5-h (G7JSE2)
Kwl3-5-g (G7L3L3)
Kwl3-5-f (G7L3J5)
Kwl3-5-l (G7JRF8)
Kwl3-5-k (G7L3M0)
Kwl3-5-j (G7L3M5)
Kwl3-5-d (G7L459)
Kwl3-5-b (G7L460)
Kwl3-5-c (Q2HRD8)
Kwl3-5-a (G7L3L5)
Kwl3-6-a (A0A072TZD6)
Kwl1-5-a (G8A171)
Kwl1-5-b (A0A072VBL0)
Kwl1-5-c (G8A174)
Cicer_arietinum
Kwl3-5-b (A0A1S2XQ19)
Kwl3-5-a (A0A1S2Y7Q6)
Kwl3-6-a (A0A1S2YW26)
Kwl1-5-a (A0A1S3E7G1)
Arachis_hypogaea
Kwl1-5-c (A0A444Y7P0)
Kwl1-5-a (A0A444Y7V7)
Kwl1-5-b (A0A444Y7Q6)
Kwl3-6-a (A0A445EIZ2)
Kwl3-6-b (A0A445ABA4)
Kwl3-5-a (A0A445E9U3)
Kwl3-5-d (A0A444ZM53)
Kwl3-5-c (A0A444ZM56)
Kwl3-5-b (A0A445DDB7)
Arachis_duranensis
Kwl3-5-b (A0A6P5NNB2)
Kwl3-5-a (A0A6P4CGF6)
Kwl3-6-a (A0A6P4BX58)
Kwl1-5-a (A0A6P4BD26)
Kwl1-5-b (A0A6P5MK77)
Juglans_regia
Kwl1-5-c (A0A2I4EJ79)
Kwl1-5-b (A0A2I4EJC7)
Kwl1-5-a (A0A2I4HTT1)
Kwl3-9-a (A0A6P9EJB1)
Kwl3-9-d (A0A2I4FSA8)
Kwl3-9-b (A0A2I4E8U3)
Kwl3-9-c (A0A2I4EBS9)
Carpinus_fangiana
Kwl3-9-a (A0A5N6QK76)
Kwl1-5-a (A0A660KQV4)
Quercus_lobata
Kwl3-9-a (A0A7N2MDH1)
Kwl1-5-a (A0A7N2KN76)
Kwl1-5-b (A0A7N2KN97)

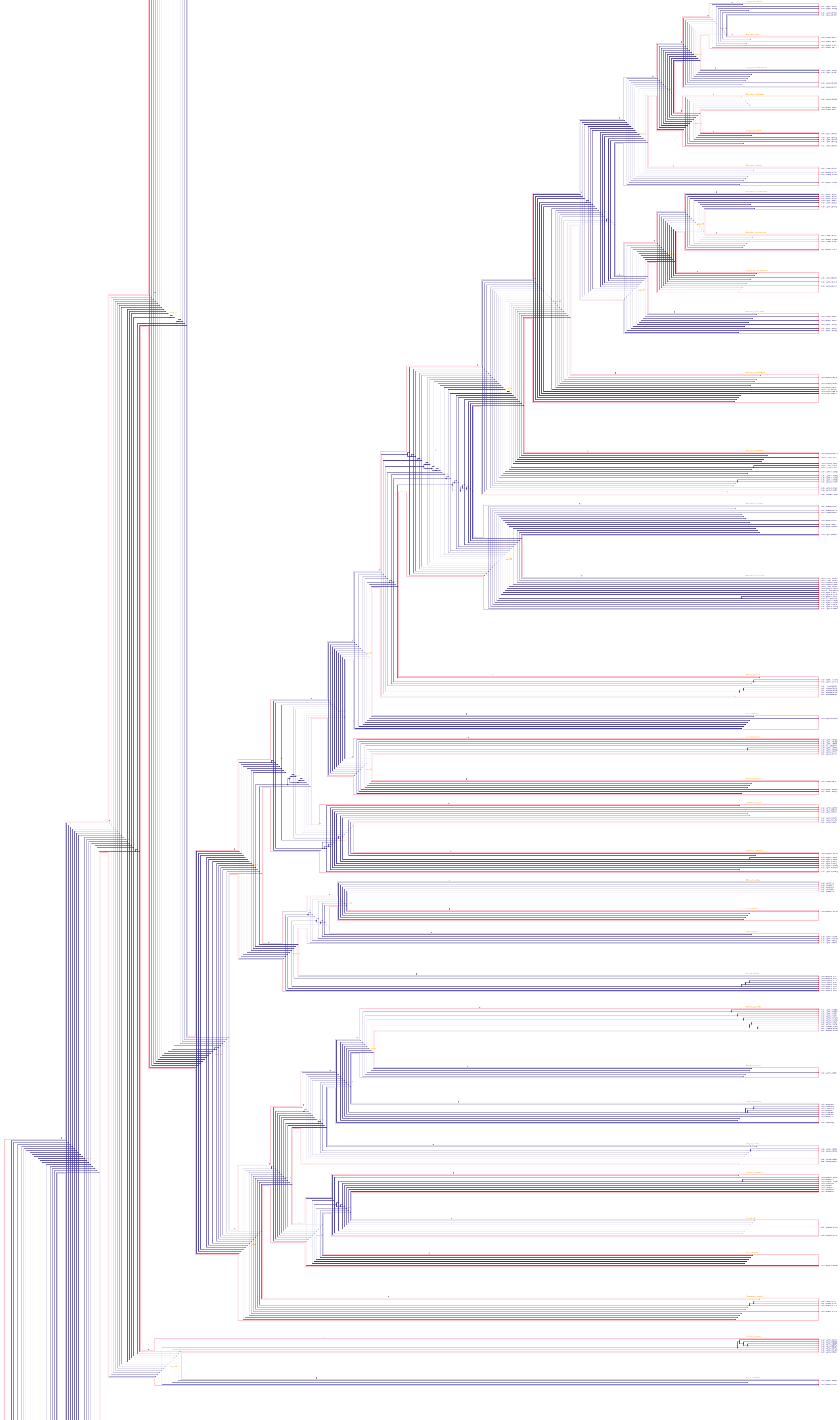
Gossypium_lobatum
Kwl3-2-a (A0A7J8M5K6)
Kwl3-9-b (A0A7J8NIP9)
Kwl3-9-a (A0A7J8ME30)
Kwl3-9-c (A0A7J8MEB1)
Gossypium_laxum
Kwl3-9-b (A0A7J9A110)
Kwl3-9-a (A0A7J9A149)
Kwl3-2-a (A0A7J8ZSX2)
Kwl1-2-a (A0A7J9A7T1)
Gossypium_armourianum
Kwl3-9-b (A0A7J9JJB6)
Kwl3-9-a (A0A7J9JJ30)
Kwl3-2-a (A0A7J9JCX8)
Kwl1-2-a (A0A7J9JPW1)
Gossypium_harknessii
Kwl3-2-a (A0A7J9GXG8)
Kwl3-9-a (A0A7J9H5R0)
Kwl3-9-b (A0A7J9H5T2)
Gossypium_aridum
Kwl3-9-c (A0A7J8XPU8)
Kwl3-9-a (A0A7J8YUJ2)
Kwl3-9-b (A0A7J8XN47)
Kwl3-2-a (A0A7J8XF50)

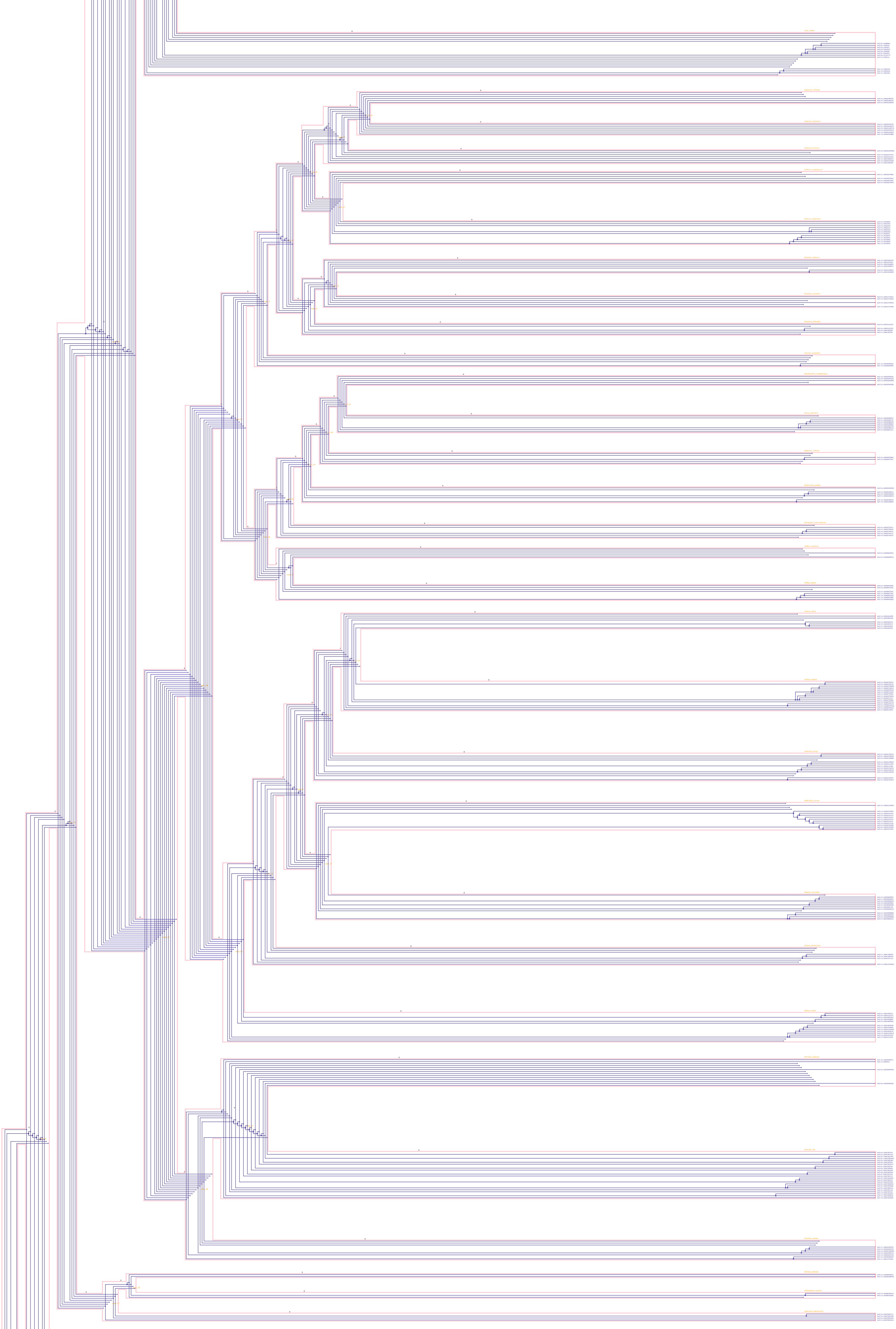

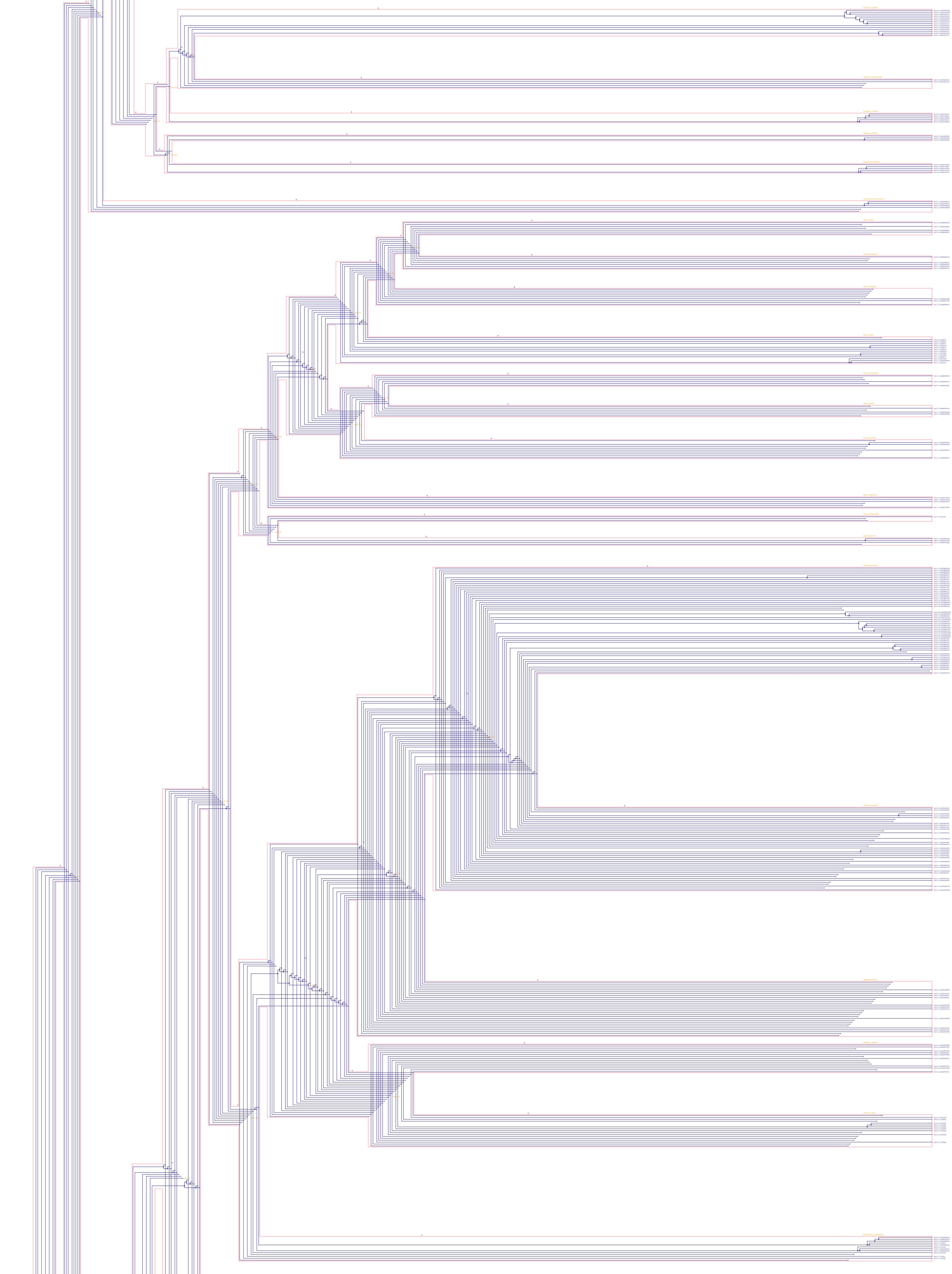
Aquilegia_coerulea
KwDI-5-n (A0A2G5DDZ0)
KwDI-5-m (A0A2G5DDY2)
KwDI-5-l (A0A2G5DET0)
KwDI-5-b (A0A2G5EU65)
KwDI-5-c (A0A2G5EU62)
KwDI-5-e (A0A2G5EU59)
KwDI-5-d (A0A2G5CXC3)
KwDI-5-f (A0A2G5CJ10)
KwDI-5-a (A0A2G5EU49)
KwDI-5-k (A0A2G5EU45)
KwDI-5-g (A0A2G5EU91)
KwDI-5-j (A0A2G5EU37)
KwDI-5-h (A0A2G5EUA0)
KwDI-5-i (A0A2G5CJ51)
Thalictrum_thalictroides
KwDI-5-b (A0A7J6VAV1)
KwDI-5-a (A0A7J6UYU9)
Kingdonia_uniflora
KwDI-5-b (A0A7J7NIV6)
KwDI-5-c (A0A7J7NJ60)
KwDI-5-d (A0A7J7P6W4)
KwDI-5-a (A0A7J7P6V3)
KwDI-5-e (A0A7J7P6W5)
Macleaya_cordata
KwDI-6-c (A0A200QNT8)
KwDI-6-b (A0A200PYN8)
KwDI-6-a (A0A200QXE9)
Papaver_somniferum
KwDI-6-a (A0A4Y7K6X8)
KwDI-6-c (A0A4Y7I826)
KwDI-6-b (A0A4Y7K783)
KwDI-6-e (A0A4Y7I6V5)
KwDI-6-d (A0A4Y7K9U1)

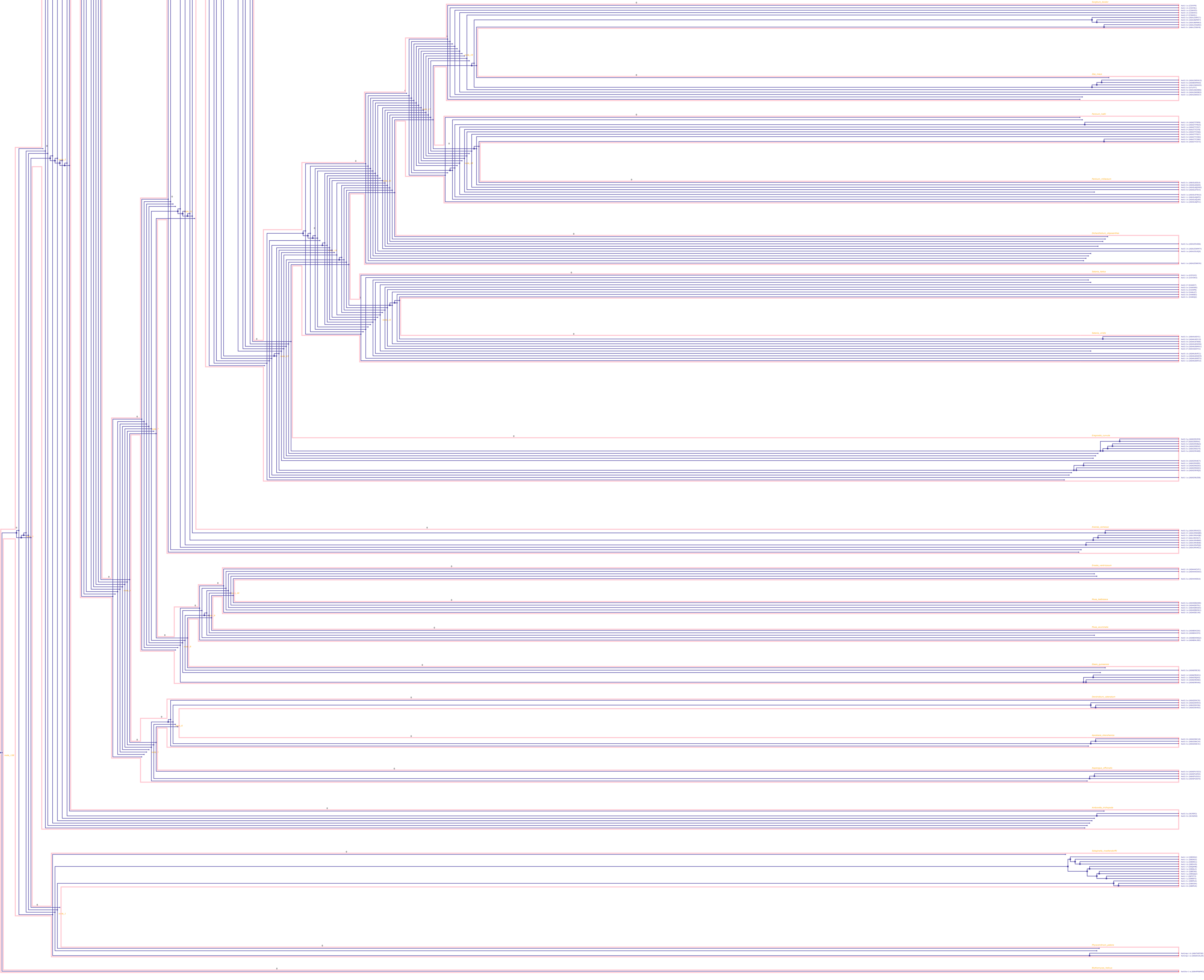

Supplement: Supplementary Data Sheet 1 — Additional figures and detailed algorithmic description (PDF). chapter 1, S1 Gene tree with and without truncated Kissper-Kiwellins. chapter 2, S2 Weblogo of consensus sequences. chapter 3, S3 Examples of kiwellin domain duplications and triplications. chapter 4 Detailed description of the kiwellin identification pipeline. S4 RMSDPMAS examples. S5 Range of descriptors used in find_kwl. S6 Kiwellin identification pipeline. chapter 5 Detailed RNA-seq results. S7 Workflow for re-evaluated RNA-seq data. [file Image_1.pdf]
